# Prevention of dementia using mobile phone applications (PRODEMOS) – a health-economic cost-utility analysis in people aged 55–75 years with low socio-economic status

**DOI:** 10.1016/j.tjpad.2026.100526

**Published:** 2026-02-27

**Authors:** Ron Handels, Marieke Hoevenaar-Blom, Manshu Song, Carol Brayne, Eric Moll van Charante, Fiona E. Matthews, Junfang Xu, Linus Jönsson, Nicola Coley, Rachael Brooks, Xuening Jian, Tingting Qin, Youxin Wang, Wei Wang, Edo Richard, Anders Wimo, PRODEMOS study group

**Affiliations:** a Alzheimer Centre Limburg, Faculty of Health Medicine and Life Sciences, School for Mental Health and Neuroscience, Department of Psychiatry and Neuropsychology, Maastricht University. Universiteitssingel 40, 6200MD, Maastricht, the Netherlands; b Division of Neurogeriatrics, Department of Neurobiology, Care Sciences and Society, Karolinska Institutet. Akademiska stråket 171 64 Solna, Sweden; c Department of General Practice, Amsterdam UMC, University of Amsterdam. De Boelelaan 1117, 1081 HV, Amsterdam, the Netherlands; d Department of Public and Occupational Health, Amsterdam UMC, University of Amsterdam. Meibergdreef 9, 1105AZ, Amsterdam, the Netherlands; e Beijing Key Laboratory of Clinical Epidemiology, School of Public Health, Capital Medical University. 10 Xitoutiao, Youanmenwai, Fengtai District, Beijing 100069, China; f Nutrition and Health Innovation Research Institute, School of Medical and Health Sciences, Edith Cowan University. 270 Joondalup Dr, Joondalup, Perth, WA 6027, Australia; g Department of Psychiatry, University of Cambridge. The Old Schools, Trinity Ln, Cambridge CB2 1TN, United Kingdom; h Institute for Clinical and Applied Health Research, University of Hull, Cottingham Rd, Hull HU6 7RX, England United Kingdom; i Institute of Social Medicine, School of Public Health, Zhejiang University School of Medicine, 866 Yuhangtang Rd, Hangzhou 310058, China; j Centre for Epidemiology and Research in Population health (CERPOP), INSERM-University of Toulouse UPS. 1 avenue Jean Poulhès - BP 84225 - 31432 Toulouse Cedex 4, France; k Department of Epidemiology and Public Health, Toulouse University Hospital. 2 Rue Charles Viguerie, 31300 Toulouse, France; l IHU HealthAge, Cité de la santé. Place Lange, 31059 Toulouse, France; m Chemistry and Chemical Engineering Guangdong Laboratory. 1 Xueyuan Road, Jinping District, Shantou 515041, Guangdong, China; n Clinical Research Centre, The First Affiliated Hospital of Shantou University Medical College. 57 Changping Rd, Jinping District, Shantou 515041, Guangdong China; o Department of Neurology, Donders Institute for Brain, Cognition and Behaviour, Radboud University Medical Centre, P.O. Box 9103, 6500 HD, Nijmegen, the Netherlands

**Keywords:** Health-economic evaluation, Cost-effectiveness analysis, Dementia, Primary prevention, Mobile health

## Abstract

**Introduction:**

We aimed to explore the potential incremental cost-effectiveness of the PRODEMOS coach-supported mobile health intervention for primary prevention of dementia versus standard of care provided to people aged 55–75 years with low socio-economic status (SES) in the United Kingdom (UK), and any SES in China.

**Methods:**

12–18-month PRODEMOS trial (ISRCTN15986016) efficacy outcomes on hypertension, obesity, hypercholesterolemia, physical inactivity and smoking were extrapolated to lifetime impact on dementia onset, myocardial infarction, stroke and death using a health-economic open-source simulation model.

**Results:**

Simulated outcomes showed dementia cases were avoided (UK = -206 (-658 to 281), China = -140 (-456 to 205) per 100,000 persons) and disease-free time was gained for dementia, myocardial infarction and stroke (mean months per person UK = 0.4, 0.0 and 0.0; China = 0.2, 0.0 and 0.0 respectively). Assuming a maximum intervention duration of 10 years with a 10 % annual non-adherence rate, the incremental net health benefit in the UK (-0.190) and China (-0.009) indicated a potential lack cost-effectiveness.

**Limitations:**

Our method was limited by strong assumptions regarding causality and sustained effectiveness, lack of some country-specific input estimates, and the lack of probabilistic analysis.

**Conclusion:**

The PRODEMOS coach-supported mobile health intervention for the primary prevention of dementia, aimed at people aged 55 to 75 years with low SES in the UK and those of any SES in China, may potentially lack cost-effectiveness in both countries. However, lack of data required strong assumptions regarding causality and sustained effectiveness, which limited policy recommendations.

## Highlights

• The PRODEMOS coach-supported mobile health intervention for the primary prevention of dementia, aimed at people aged 55 to 75 years with low SES in the UK and those of any SES in China, may potentially lack cost-effectiveness in both countries. However, the results were based on strong assumptions regarding causality and sustained effectiveness, which limits policy recommendations.

## 1 Introduction

The prevalence of dementia is expected to increase to over 150 million in 2050, mostly in low- and middle-income countries and populations with low socio-economic status (SES) in high-income countries [1,2]. Approximately 45 % of dementia cases are associated with modifiable (lifestyle) risk factors, such as hypertension, smoking, obesity and physical inactivity [3]. Mobile health interventions are considered a promising method to reach people who have limited access to preventive services [4], targeting risk factors in a personalized remote manner [5] and potentially preventing or delaying the onset of dementia. However, evidence on effectiveness and cost-effectiveness in low/middle income country and low SES populations are lacking.

A mobile health intervention was evaluated for effectiveness in the PRODEMOS randomized trial in the United Kingdom (UK) and China setting [6]. It targeted the reduction of dementia risk factors by exposing participants – aged 55–75 years with low SES in the UK and any SES (i.e., general population) in China, all considered at increased risk for dementia – to a coach-supported mobile health application. It captured changes in their risk factor status over a 12–18-month follow-up period, compared to a control arm receiving a non-interactive mobile health application without coach. The trial’s primary outcome – which includes modifiable risk factors hypertension, obesity, high cholesterol and physical inactivity – showed a small, but statistically significant reduction in modifiable risk factors in the intervention arm compared to the control arm. Their combined effect was captured in a dementia risk score named “Cardiovascular Risk Factors, Aging, and Incidence of Dementia” (CAIDE), which showed a change from baseline to the end of the trial of −0.16 with 95 %CI −0.29 to −0.03 [6].

For health-care systems it is important to assess the impact of new interventions on long-term health-economic outcomes to support deciding upon allocation of limited care resources. This requires extrapolation of change in risk factors beyond the trial 12–18-month time horizon to impact on dementia onset generally occurring in late-life and their associated health-economic outcomes. Various studies have estimated the (potential) health or health-economic impact of interventions for primary prevention of dementia using a decision-analytic model [[7], [8], [9], [10], [11], [12], [13], [14], [15]]. However, few studies included spill-over effects on cardiovascular disease (CVD) [8,12] as dementia and CVD have a similar set of modifiable risk factors [3,16]. This limits the cost-effectiveness estimates of interventions like PRODEMOS that aim to improve modifiable (lifestyle) risk factors.

The aim of this study was to explore the potential incremental cost-effectiveness of the PRODEMOS coach-supported mobile health intervention for the primary prevention of dementia versus standard of care provided to people aged 55 to 75 years with low SES in the UK, and any SES in China. The focus was on developing a model that captures the impact on both dementia and CVD.

## 2 Methods

The PRODEMOS trial (duration 12–18 months) was performed in the UK and China using the same intervention but with some differences in target population (detailed below). It was considered too short to capture an effect on dementia and CVD incidence. Therefore, the use of a health-economic simulation model plan was pre-defined [17] to extrapolate the short-term PRODEMOS trial results on modifiable risk factors to potential long-term effects on incidence and prevalence of dementia and CVD, related quality-adjusted life years (QALY) and costs, and their uncertainty.

We employed a societal perspective by including resource use in the health sector and by patient/family as both contain a large part of dementia care [18]. Uncertainty was addressed by univariate sensitivity analysis. A lifetime horizon was operationalized by simulating up to the age of 100. Future costs and effects were discounted at an annual rate of 3.5 % for the UK [19] and 5 % for China [20]. A willingness to pay threshold of £20,000 per gained QALY was used for the UK [19] and the country-level gross domestic product per capita for China [20,21], which was 81,419 Chinese yuan in 2021 [22].

### 2.1 Target setting and population

We followed the target population and setting of the PRODEMOS trial. This consisted of the primary care population in the UK and the general population recruited from both medical and community settings in China, reflecting the trial’s recruitment strategy [23]. The target population was people aged 55 to 75 years with two or more dementia risk factors, reflecting the trial’s in- and exclusion criteria (**supplementary material 1**). The PRODEMOS trial recruitment strategies differed between countries due to contextual and public-health priorities rather than assumptions of differential intervention effectiveness. In the UK, the PRODEMOS trial specifically targeted adults with low socioeconomic status (SES) given their higher burden of modifiable dementia and cardiovascular risk factors and their historical under-representation in digital prevention programs. In China, we did not specifically focus on low SES groups, as it is a middle-income country facing the same disproportionate burden of modifiable risk factors as people with low SES in high income countries.

The following trial baseline factors were expected to be associated with dementia and CVD incidence: age, sex, education, deprivation, type 2 diabetes, depression, history of dementia, history of myocardial infarction (MI), history of stroke, body mass index (BMI), systolic blood pressure (SBP), total cholesterol, physical inactivity and smoking (**supplementary material 2**). These factors were originally planned to be used to simulate the expected dementia and CVD incidence of the target population (**supplementary material 3**, specifically 3.1, 3.2 and 3.3). However, the already overestimated prevalence (see paragraph validation) was considered a reflection with greater face validity of the increased risk in the target population by expert opinion and was therefore used as base case.

### 2.2 Comparators

The model reflected an intervention strategy where the PRODEMOS coach-supported mobile health intervention was provided alongside standard of care and compared it to a strategy reflecting standard of care alone.

In summary, the PRODEMOS intervention consisted of an interactive smartphone application that facilitated coach-supported self-management of seven modifiable dementia risk factors (overweight, unhealthy diet, insufficient physical activity, smoking, hypertension, dyslipidemia and diabetes). Participants discussed their individual dementia risk profile with a coach and set personalized goals for lifestyle behavior change, supported by their coach, who was trained and experienced in motivational interviewing and behavior change techniques. Participants received automated reminders to enter lifestyle-related measurements for monitoring, tailored education modules and remote (messaging-based) support from the coach to facilitate sustainable behavior change [23,24]. The intervention required on average 30 and 17 h of coaching time per participant per year in the UK and China, respectively. Coaches were required to hold at least a bachelor’s degree in a health-related discipline (e.g., medical sciences, nutrition, nursing, allied health, health sciences, or public health) and to demonstrate competencies in motivational interviewing, lifestyle behaviour change, goal setting, and progress monitoring. In China, these competencies were acquired through approximately 40–60 h of structured training, supplemented by practical exercises. In the UK, training consisted of 16–24 h (depending on prior experience and skills), three half-day internships (with extension if required), and supervised practice. Ongoing supervision during the intervention period was provided in China through demonstrations and feedback during monthly intervision meetings, and in the UK through individual supervision supplemented by bimonthly intervision meetings. The anticipated caseload ranged from 48 to 85 participants per coach per year (**Supplementary material 3**, specifically Section 3.6.5).

The standard of care strategy was assumed to be reflected by the PRODEMOS trial control arm, which provided access to a smartphone application similar in appearance but only containing educational materials, without interactive features and coach-support. Participants in the control arm received concise feedback on their risk profile at baseline only in case of concerned risk factors. We note differences in standard of care between UK and China. These differences were reflected by country specific disease incidence, mortality and cost estimates.

### 2.3 Intervention effectiveness and waning

The PRODEMOS trial’s primary endpoint was the CAIDE dementia risk score, which includes four modifiable factors (SBP, BMI, cholesterol and physical activity) and three non-modifiable factors (age, sex and education) and combines them into a single score [25]. These CAIDE modifiable factors and the secondary outcome of smoking (five factors in total) were chosen to reflect the intervention effect as they were considered related to both dementia [25] and CVD [26]. **Supplementary material 2** provides their definitions, units and mapping.

These results were translated to a relative risk of developing dementia and CVD using risk models based on CAIDE for dementia and QRISK for cardiovascular disease. For example, hypertension has a relative risk of 2.1 in the CAIDE risk score and was reduced by 1.1 % in the PRODEMOS trial [6], which corresponds to a relative risk of 0.992. Similarly, systolic blood pressure has a relative risk of 1.005 per mmHg increase (female) and was reduced by 0.8 mmHg in the PRODEMOS trial, which corresponds to a relative risk of 0.997. The product of all relative risks was taken (**supplementary material 3**, specifically 3.4 for calculation details). Overall, this translated the PRODEMOS intervention effect on risk factors to a relative risk of 0.924 (95 % bootstrap interval 0.768–1.108) (relative risk reduction of 7.6 %) for dementia, and 0.982 (0.946–1.018) and 0.980 (0.942–1.018) for male and female respectively for both MI and stroke (relative risk reduction of 1.8 % and 2.0 %). This effect was applied both to UK and China to align with the PRODEMOS trial effectiveness analysis, as no significant difference in effect according to country was observed [6].

Evidence, including findings from existing systematic reviews, was deemed too limited. Therefore, for the base case, an ad-hoc arbitrary assumption was applied: the intervention was assumed to continue for a maximum of 10 years with an annual non-adherence rate of 10 % (i.e., people quitting the intervention). No subsequent intervention costs were applied for those who quit, and the intervention effect was reflected by raising the relative risk to the power of the adherence rate to the power of the time since model start (**supplementary material 4**).

### 2.4 Rationale for model design

At the start of the project in 2019, a scoping review described the limited ability of current dementia-specific prevention models to reflect plausible effects on CVD [27], raising the potential of general health models such as Population Ageing and Care Simulation model [28], Future Elderly Model [29] and Dynamic Aging Process [8]. However, these were considered too detailed to replicate, and availability of the UK and China specific costs and utility data attributable to numerous comorbidities appeared limited. Therefore, a multimorbidity model was developed focusing on high-impact brain and cardiovascular diseases – dementia, MI and stroke – associated with the risk factors targeted by the PRODEMOS intervention. Such an approach is similar to a model on coronary heart disease, stroke and type 2 diabetes incidence, and its relation to physical activity [30]. Our approach was developed in 2021 and considered face valid by modelers [RH, AW, CG, LJ], clinicians [ER, EMC] and epidemiologists [CB, NC, MHB, FEM]. Neither the target population nor persons with dementia or MI or stroke were involved in co-designing the model. The model was programmed in a spreadsheet. The base case scenario of the model was also programmed R and is available open-source on GitHub, version 1.0.0: [https://github.com/ronhandels/prodemos].

### 2.5 Model structure

**Fig. 1:**
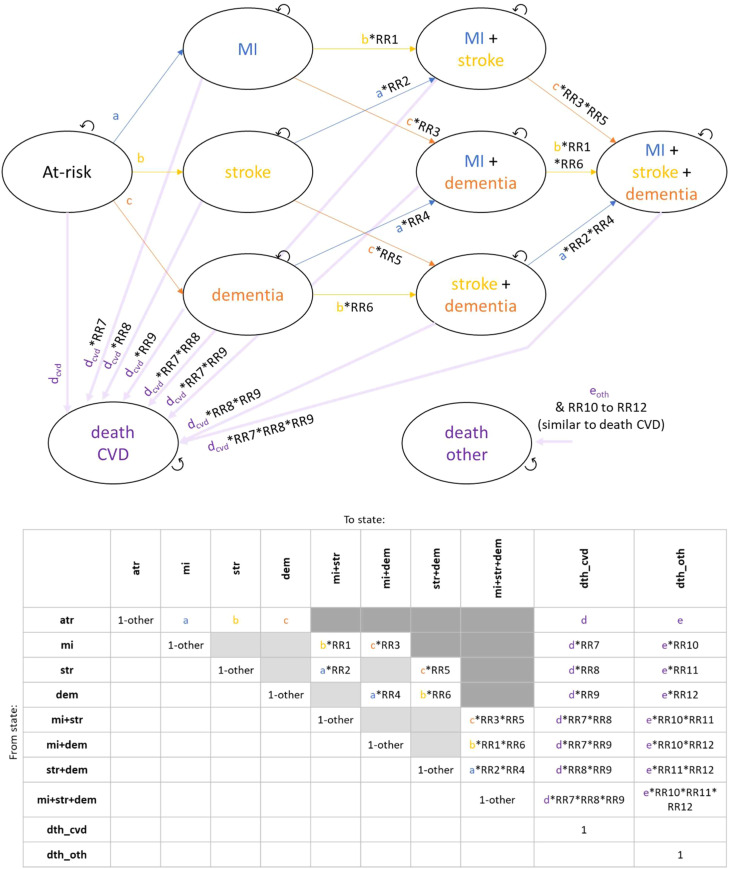
Visualization of the model; transitions to non-death conditional on survival (i.e., transitions to death were handled first). a,b,c,d,e linked to arrows represent rates, RR1 to RR12 represent relative risks.

Abbreviations: atr, at risk; cvd, cardiovascular disease; dem, dementia; dth, death; mi, myocardial infarction; oth, other; RR, relative risk; str, stroke.

A cohort state transition Markov model was developed with a 12-month cycle length. It contained states at-risk, history of dementia, history of MI, history of stroke and death, as well as all possible combinations of the 3 diseases to allow reflecting their interactive effect. In summary, the model was built up as follows: 1) disease onset from general population age, sex and disease-history specific incidence rates for dementia, MI and stroke from the second Cognitive Function and Ageing Study (CFAS-II) [31]; 2) age, sex and disease-history specific mortality from general population life tables and relative risks for disease history; 3) starting population reflecting age band, sex and disease-history prevalence as observed in the PRODEMOS trial, and increased risk of disease incidence related to the higher average risk factor status in the PRODEMOS baseline sample compared to the general population; 4) intervention effect reflected by the relative risk related to the change in risk factor status between the PRODEMOS intervention and control arm; 5) Markov model state trace; 6) person-years living with a history of the disease and incident events multiplied with quality of life based utility and cost estimates. The model details are presented in **supplementary material 3** and illustrated in Fig. 1.

### 2.6 Input estimates

Input estimates were obtained from existing reviews, previous (NICE technology appraisal) decision-analytic models, individual patient-level databases familiar to the authors, PRODEMOS trial data and expert opinion. We selected sources based on the extent to which they reflected the target setting and population, and alignment with other estimates. Costs were converted using exchange rates to pound sterling (£) or Chinese renminbi (¥) using exchange rates and converted to 2021 using consumer price indices. Table 1 provides a summary overview of the model input estimates and reflection of uncertainty.

**Table 1:** Overview of model inputs.

Item	Estimate UK	Source and comments UK	Estimate China	Source and comments China	Details
*DISEASE INCIDENCE*					
General population incidence rate of dementia onset, MI and stroke	GLM coefficients	CFASII GLM age, sex, int. age sex, hist. dementia, hist.MI, hist. stroke			S3.1
Dementia constant (coefficient)	−12.40		−13.91	[52]	
Dementia age (coefficient)	0.11		0.13	[52]	
Dementia sex (coefficient)	0.16		0.34	[52]	
Dementia history dementia (RR)	n/a		n/a		
Dementia history MI (RR)	Not included		Not included		
Dementia history stroke (RR)	1.71		Not included		
MI constant (coefficient)	−7.50		−9.62	[53]	
MI age (coefficient)	0.03		0.06	[53]	
MI sex (coefficient)	0.39		−0.34	[53]	
MI history dementia (RR)	Not included		Not included		
MI history MI (RR)	7.59		Not included		
MI history stroke (RR)	Not included		Not included		
Stroke constant (coefficient)	−6.84		−9.82	[54]	
Stroke age (coefficient)	0.02		0.08	[54]	
Stroke sex (coefficient)	0.20		−0.26	[54]	
Stroke history dementia (RR)	Not included		Not included		
Stroke history MI (RR)	2.11		Not included		
Stroke history stroke (RR)	4.51		Not included		
*DEATH INCIDENCE*					
General population mortality	Life table	^a^	Life table	^b^	S3.2.1
Proportion death related to CVD	Table	^a^	assumed same as UK		S3.2.1
RR of death by history of dementia, MI and stroke	GLM	CFASII			S3.2.2
RR death-CVD (history dementia)	5.82	CFASII	3.02	[55]	
RR death-CVD (history MI)	1.43	CFASII	4.3	assumed same as stroke	
RR death-CVD (history stroke)	1.27	CFASII	4.3	[54]	
RR death-other (history dementia)	5.82	CFASII	3.02	assumed same as death-CVD	
RR death-other (history MI)	1.43	CFASII	4.3	assumed same as death-CVD	
RR death-other (history stroke)	1.27	CFASII	4.3	assumed same as death-CVD	
Prevalence to adjust base rate mortality table					S3.2.3
Dementia	table	CFASII	table	[56]	
MI	table	CFASII	table	assumed same as stroke	
Stroke	table	CFASII	table	[54]	
*TARGET POPULATION*					
Proportion age band and sex	Table	PRODEMOS baseline subsample UK age bands (55–59, 60–64, 65–69, 70–74)	Table	PRODEMOS baseline subsample age bands (55–59, 60–64, 65–69, 70–74)	S3.3.1
History of MI and stroke (proportion by age band and sex)	Table	PRODEMOS baseline subsample UK	Table	PRODEMOS baseline subsample China	S3.3.1
*INTERVENTION EFFECT*					
RR dementia	0.924	[6]	0.924	[6]	S3.4
RR MI and stroke	*M* = 0.982 *F* = 0.980	[6]	*M* = 0.982 *F* = 0.980	[6]	S3.4
Duration intervention	10 years	assumption	10 years	assumption	S4
Non-adherence	10 % per year	assumption	10 % per year	assumption	S4
*UTILITIES*					
general population	0.9508566 + 0.0212126*male + 0.0002587*age −0.0000332*age^2	[57]	0.9091741 + 0.0050488*male + 0.0023842*age −0.0000319*age^2	[58]	S3.6
Ratio utility dementia (onset and history) and general population	0.876	[59,60]	0.764	[58]	S3.6
Ratio utility MI history versus general population	0.923	[57]	0.939	assumed same as UK	S3.6
Disutility MI event versus MI history	−0.025	[57]	−0.025	[58]	S3.6
Ratio utility stroke history versus general population	0.839	[57]	0.774	assumed same as UK	S3.6
Disutility stroke event versus stroke history	−0.048	[57]	−0.048	[58]	S3.6
*COSTS £ (UK) or ¥ (China)*					
Dementia health sector post-year 1	£7973	[[60], [61], [62], [63]]	¥20,973	[64]	S3.6
Dementia health sector additional in year 1	£0	Assumption	¥0	Assumption	S3.6
Dementia informal care post-year 1	£10,028	[[60], [61], [62], [63]]	¥65,826	[64]	S3.6
Dementia informal care additional in year 1	£0	Assumption	¥0	Assumption	S3.6
MI health sector post year 1	£276	[[65], [66], [67]]	¥2920	[68]	S3.6
MI health sector additional in year 1	£6554	[[65], [66], [67]]	¥29,196	[[68], [69], [70]]	S3.6
MI informal care post-year 1	£0	Assumption	¥0	Assumption	S3.6
MI informal care additional in year 1	£0	Assumption	¥0	Assumption	S3.6
Stroke health sector post-year 1	£4116	[71]	¥10,401	[68]	S3.6
Stroke health sector additional in year 1	£10,714	[71]	¥11,752	[[68], [69], [70]]	S3.6
Stroke informal care post-year 1	£6953	[71]	¥17,570	Assumption	S3.6
Stroke informal care additional in year 1	£0	[71]	¥0	Assumption	S3.6
Intervention costs platform per user per year	£5	Assumption	¥38	Provided by platform manufacturer	S3.6
Intervention costs coaching per user per year	£800	PRODEMOS trial data	¥510	PRODEMOS trial data	S3.6
Duration platform (years)	10 years	Assumption	10 years	Assumption	S3.6
*OTHER*					
Discount costs (rate)	0.035	[19]	0.05	[20]	
Discount effects (rate)	0.035	[19]	0.05	[20]	
Willingness to pay	£20,000	[19]	¥81,419	[20,21]	

Abbreviations: CVD, cardiovascular disease; GLM, general linear model; MI, myocardial infarction; n/a, not applicable; RR, relative risk; UK, United Kingdom. ^a^www.ons.gov.uk. ^b^ China Population and Employment Statistics Yearbook Committee. Section 2–44. National Death Population Situation by Age and Sex (November 1, 2018, to October 31, 2019), in China Population and Employment Statistics Yearbook 2020, September 2020, Beijing: China Statistics Press. Available at: https://www.zgtjnj.org/navipage-n3020013208000178.html [In Chinese].

### 2.7 Model assumptions

Two key assumptions underpin the analysis. First, a causal relationship between lifestyle change and risk modification was assumed. For example, the model assumes that reducing physical inactivity lowers dementia risk. However, the evidence supporting this assumption remains observational, as no randomized controlled lifestyle-intervention trials have yet demonstrated a significant reduction in dementia incidence, possibly due to short trial follow-up periods. Second, the model assumes that lifestyle improvements persist beyond the PRODEMOS trial follow-up period. This assumption was necessary due to limited evidence on long-term effectiveness, as discussed in the section on intervention effectiveness and waning. Uncertainty related to these assumptions were addressed through sensitivity analyses and further discussed.

### 2.8 Analytical methods

Cumulative incidence was calculated by summing lifetime disease and death events of the simulated cohort. Person-years were estimated by summing the time spent with each disease by the simulated cohort over lifetime. Lifetime mean QALYs and costs were calculated by multiplying utilities and costs corresponding to history and (recurrent) events of disease. Incremental net health benefit and incremental cost-effectiveness ratio were calculated using Eq. (1). QALYs and costs were discounted.

### 2.9 Validation

The internal validity of the simulation model was tested using extreme input parameters and the external validity by comparing the simulated prevalence using a non-at-increased-risk population to the observed prevalence from the partially dependent data from the CFAS-II study [31] (**supplementary material 5**). We visually assessed these results and judged the model as having moderate (i.e., simulated prevalence outside but not more than two times beyond the observed confidence interval) to good (i.e., simulated prevalence within observed confidence interval) external validity. We noted a possible simulated overestimation of dementia prevalence and underestimation of MI prevalence in males.

### 2.10 Heterogeneity and uncertainty

Sensitivity analyses were performed to address heterogeneity and uncertainty. Heterogeneity was reflected by simulating a starting age of 60 and 70, for men and women separately for their general difference in life expectancy. Sensitivity to input estimates or assumptions was addressed by univariate sensitivity analyses only in terms of time horizon, disease incidence, effect size, intervention costs, baseline risk, discounting to address the uncertainty of the model outcomes (**supplementary material 6**). In contrast to best practice recommendations Probabilistic sensitivity analysis was not performed as plausible distributions were lacking for the assumption on causality and the assumption on lifetime intervention adherence. As these had a high impact on model outcomes, omitting them in a probabilistic analysis would misrepresent uncertainty. The alternative method of expert elicitation on these assumptions fell outside the scope of this study.

Sensitivity to the time horizon was assessed by simulating up to the age of 90 and 80 years (instead of 100).

Sensitivity of the results to the method of simulating disease and mortality was assessed in 8 different ways. First, risk factor relative risks (used to translate the treatment effect into dementia onset, MI event and stroke event) were obtained by the Lifestyle for BRAin Health (LIBRA) [32] and QRISK3 [33] studies (instead of CAIDE and QRISK1). This resulted in a weaker relative risk related to the PRODEMOS intervention (0.94 instead of 0.92 for dementia onset and 0.98–0.97 instead of 0.98 for MI and stroke). Second, a starting cohort without a history of MI and stroke was used. Third, the first and second were combined. Fourth, the relative risk for dementia related to the PRODEMOS intervention was estimated using the CAIDE risk total score (instead of its individual coefficients). This resulted in a relative risk of 0.95 (instead of 0.92), although this excluded the effect of smoking. Fifth, a dementia-only model was employed. Sixth, alternative data sources and methods for disease incidence were used. Seventh, alternative sources for CVD-specific mortality risk were used. Eighth, a target population at increased risk was applied.

Sensitivity to the non-adherence (i.e., sustainability of the intervention effect), cost and duration were assessed in 8 different ways. First, using the effectiveness estimate in the trial’s adherent subgroup. Second, using the trial’s subgroup of those planning to change their lifestyle within 6 months. Third, using the effectiveness estimate in the trial’s country-specific subgroups. Fourth, assuming no coach support when repeating the intervention (phone application only). Fifth and sixth, assuming lifetime full adherence with and without coach support when repeating the intervention beyond the trial duration. Seventh and eighth, assuming lower (1/3) and higher (3 times) intervention costs.

Sensitivity to discount rate (1 % and 7 %) was assessed. Sensitivity to willingness to pay was assessed by adopting £30,000 per QALY in the UK and 3 times the gross domestic product (GDP) per QALY in China following guideline recommendations [[19], [20], [21]].

The possible Hawthorne effect (assumed represented by an outcome improvement in the PRODEMOS non-blind trial control arm) was relatively small (change in CAIDE risk score in trial control arm was 0.01 versus 0.16 in intervention arm) and therefore not addressed in the sensitivity analysis.

## 3 Results

The model simulated a cohort of 100,000 individuals reflecting the PRODEMOS target population at increased risk for dementia both for the standard of care and when offered the PRODEMOS intervention (Table 2). For the UK, in the standard of care strategy the mean survival in the targeted population was 20.7 years and the lifetime dementia risk was 45 % in our at increased risk target population. Compared to the standard of care the intervention prevented 206 (95 % bootstrap interval −658 to 281) dementia onset events, 55 (−151 to 32) MI events and 71 (−193 to 41) stroke events out of the 100,000 individuals. Additionally, the mean disease time per person in the intervention strategy was lower for dementia (−0.3 months), and was smaller than 0.1 month for MI and stroke. The mean disease-free time per individual was higher for dementia-free (+0.7 months), MI-free (+0.5 month) and stroke-free (+0.5 months).

**Table 2:** 30-year model outcomes in UK and China, mean per person unless stated otherwise; Quality-adjusted life years (QALY) and costs discounted; costs indexed to 2021; interval reflects the model outcomes corresponding to the 2.5th and 97.5th percentile of the relative risk for dementia, MI and stroke based on random (bootstrap) draws from the separate risk factors uncertainty distribution.

	UK (£)				China (¥)			
	standard of care	Inter-vention	difference	Difference (%)	standard of care	Inter-vention	difference	Difference (%)
**Cumulative incidence (per 100,000)**								
dementia onset events	44,623	44,417	−206 (−658, 281)	−0.5 %	31,870	31,730	−140 (−456, 205)	−0.4 %
MI events (including recurrent)	15,905	15,850	−55 (−151, 32)	−0.3 %	12,996	12,971	−25 (−69, 16)	−0.2 %
stroke events (including recurrent)	20,401	20,330	−71 (−193, 41)	−0.3 %	27,858	27,820	−38 (−100, 19)	−0.1 %
**Person-years (months for difference)**								
with dementia	1.8	1.8	−0.3 (−0.9, 0.4)	−1.3 %	1.7	1.7	−0.2 (−0.8, 0.3)	−1.2 %
with MI history	2.0	2.0	−0.0 (0.0, −0.0)	−0.1 %	1.2	1.2	−0.0 (−0.1, 0.0)	−0.2 %
with stroke history	2.3	2.3	−0.0 (−0.1, 0.0)	−0.2 %	2.8	2.7	−0.0 (−0.1, 0.0)	−0.1 %
without dementia	18.8	18.9	0.7 (2.3, −1.0)	0.3 %	18.7	18.8	0.5 (1.6, −0.7)	0.2 %
without MI history	18.7	18.7	0.5 (1.5, −0.6)	0.2 %	19.3	19.3	0.3 (0.9, −0.3)	0.1 %
without stroke history	18.4	18.4	0.5 (1.5, −0.6)	0.2 %	17.7	17.7	0.3 (0.9, −0.3)	0.1 %
Alive	20.7	20.7	0.4 (1.4, −0.6)	0.2 %	20.5	20.5	0.3 (0.8, −0.3)	0.1 %
QALYs	10.50	10.52	0.02 (0.05, −0.02)	0.2 %	10.84	10.86	0.01 (0.04, −0.01)	0.1 %
**Costs**								
health care	16,112	15,941	−172 (−534, 217)	−1.1 %	36,534	36,215	−320 (−1001, 419)	−0.9 %
informal care	19,802	19,595	−207 (−644, 265)	−1.0 %	73,512	72,604	−909 (−2875, 1245)	−1.2 %
platform	-	28	28 (28, 28)	n/a	-	201	201 (201, 201)	n/a
coaching	-	4477	4477 (4492, 4460)	n/a	-	2724	2724 (2730, 2718)	n/a
total	35,914	40,041	4127 (3343, 4970)	11.5 %	110,047	111,744	1697 (−945, 4582)	1.5 %
net health benefit	8.704	8.514	−0.190 (−0.115, −0.271)	−2.2 %	9.492	9.483	−0.009 (0.048, −0.071)	−0.1 %

For China, in the standard of care strategy the mean survival time was 20.5 years the lifetime dementia risk was 32 % in our target population. Compared to this standard of care the intervention prevented 140 (95 % bootstrap interval −456 to 205) cases of dementia and 25 (−69 to 16) MI events and 38 (−100 to 19) stroke events out of the 100,000 individuals. Additionally, the mean disease time per individual in the intervention strategy was lower for dementia (−0.3 months) and was smaller than 0.1 month for MI and stroke. The mean disease-free time per individual was higher for dementia-free (+0.5 months), MI-free (+0.3 months) and stroke-free (+0.3 months).

**Fig. 2:**
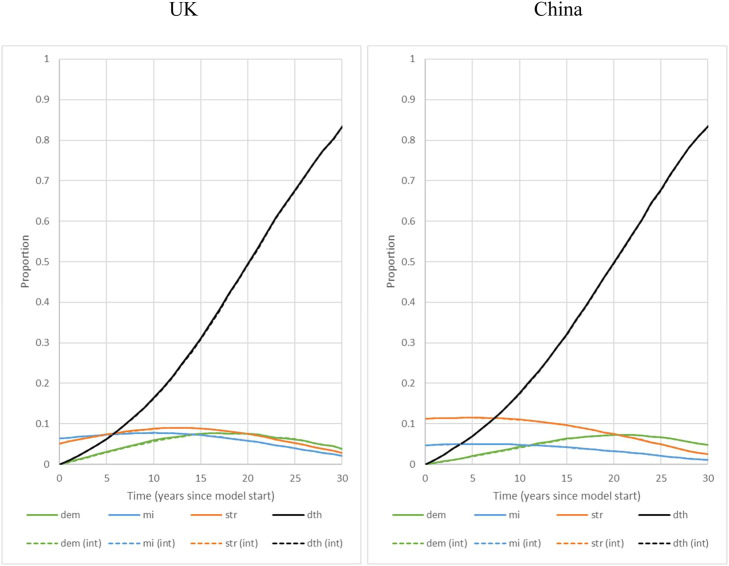
Proportion among the starting cohort with a history of dementia, MI and stroke, and death (vertical axis) by time since model start (horizontal axis), as predicted by the model, weighted for age and sex prevalence at model start, for the standard of care (solid line) and intervention strategy (dashed line) by country (left = UK, right = China). E.g., for the UK, after 15 years, approximately 8 % has developed dementia, 7 % has a history of MI, 9 % has a history of stroke and 31 % has died (the latter could include persons who died with a history of dementia, MI or stroke, explaining the decline in proportion with disease after a long time). Difference between standard of care (solid line) and intervention strategy (dashed line) almost overlap due to the difference being relatively small.

Abbreviations: dem, dementia; dth, death; int, intervention; mi, myocardial infarction; str, stroke.

Fig. 2 presents the model outcomes in terms of simulated disease prevalence and mortality over time showing the magnitude of intervention effect.

Mean per-person platform and coaching costs were £4505 in the UK and ¥2925 in China. Health care and informal care costs were reduced by 1.1 and 1.0 % in the UK and 0.9 and 1.2 % in China by the intervention strategy. QALYs increased by 0.2 % in the UK and 0.1 % in China by the intervention strategy. Given the willingness to pay per QALY this led to a negative incremental net health benefit in the UK (−0.190) and China (−0.009) (Table 2), reflecting the intervention may potentially lack cost-effectiveness.

### 3.1 Heterogeneity and uncertainty

Results of heterogeneity and uncertainty sensitivity analyses are presented in Table 3. Overall, model outcomes varied widely, reflecting high sensitivity to model input estimates and assumptions and therewith the uncertainty of our study results. This led to a range in incremental net health benefit related to uncertainty scenarios of −0.64 to 0.03 for the UK and −0.08 to 0.07 for China.

**Table 3:** 30-year model outcomes in UK and China from heterogeneity and sensitivity analyses, mean difference (intervention strategy minus standard of care strategy); Quality-adjusted life years (QALY) and costs discounted; costs indexed to 2021.

	Cumulative incidence (per 100,000)			Person-time with (months per person)				Person-time without (months per person)			Quality-adjusted life years (per person)	Cost (per person)	Net health benefit (per person)
	**Dementia**	**Myocardial infarction**	**Stroke**	**Dementia**	**Myocardial infarction**	**Stroke**	**Alive**	**Dementia**	**Myocardial infarction**	**Stroke**			
UK													
Base case	-206	-55	-71	-0.3	0.0	0.0	0.4	0.7	0.5	0.5	0.02	4,127	-0.19
**Heterogeneity**													
Age 60 male	-118	-44	-66	-0.2	0.0	-0.1	0.3	0.5	0.3	0.3	0.01	4,363	-0.21
Age 60 female	-105	-70	-88	-0.3	-0.1	-0.1	0.3	0.6	0.4	0.4	0.01	4,301	-0.20
Age 70 male	-304	-42	-54	-0.3	0.0	0.0	0.5	0.8	0.5	0.5	0.02	3,983	-0.18
Age 70 female	-280	-58	-70	-0.4	0.0	0.0	0.6	1.0	0.6	0.6	0.02	3,915	-0.17
**Time horizon**													
Up to age 90	-280	-67	-85	-0.3	0.0	-0.1	0.3	0.6	0.4	0.4	0.01	4,104	-0.19
Up to age 80	-327	-73	-95	-0.2	0.0	-0.1	0.1	0.3	0.1	0.1	0.01	4,043	-0.20
**Disease and death risk methodology**													
LIBRA & QRISK3	-150	-96	-124	-0.2	-0.1	-0.1	0.3	0.5	0.4	0.4	0.01	4,175	-0.20
No CVD history	-193	-46	-65	-0.3	-0.1	-0.1	0.4	0.7	0.5	0.5	0.02	4,125	-0.19
No CVD history, LIBRA & QRISK3	-140	-79	-110	-0.2	-0.1	-0.1	0.3	0.5	0.4	0.5	0.01	4,178	-0.20
CAIDE score mean change	-148	-66	-85	-0.2	0.0	-0.1	0.3	0.5	0.4	0.4	0.01	4,205	-0.20
Dementia-only	-192	0	0	-0.2	0.0	0.0	0.3	0.5	0.3	0.3	0.01	4,321	-0.20
Alternative disease incidence	-236	-26	-88	-0.4	0.0	0.0	0.6	1.0	0.6	0.6	0.02	3,939	-0.17
Campbell RR	-206	-53	-69	-0.3	0.0	0.0	0.5	0.7	0.5	0.5	0.02	4,132	-0.19
Target population at increased risk	-241	-14	-22	-0.7	0.1	0.0	0.9	1.6	0.9	0.9	0.04	3,267	-0.13
**Intervention effect, cost and duration**													
Adherent subgroup (dementia effect only using CAIDE score mean change)	-257	-45	-58	-0.4	0.0	0.0	0.5	0.9	0.6	0.6	0.02	4,057	-0.18
Planned lifestyle change (dementia effect only)	-310	-35	-45	-0.4	0.0	0.0	0.7	1.1	0.6	0.7	0.02	3,984	-0.18
UK-specific effect (dementia effect only using CAIDE score mean change)	-87	-78	-100	-0.1	-0.1	-0.1	0.2	0.3	0.3	0.3	0.01	4,290	-0.21
Intervention repeated without coaching	-206	-55	-71	-0.3	0.0	0.0	0.4	0.7	0.5	0.5	0.02	1,134	-0.04
Lifetime full adherence with coaching	-1812	-156	-192	-1.2	0.1	0.1	2.0	3.1	1.9	1.9	0.06	3,453	-0.12
Lifetime full adherence without coaching	-1812	-156	-192	-1.2	0.1	0.1	2.0	3.1	1.9	1.9	0.06	458	0.03
Costs x1/3	-206	-55	-71	-0.3	0.0	0.0	0.4	0.7	0.5	0.5	0.02	1,123	-0.04
Costs x3	-206	-55	-71	-0.3	0.0	0.0	0.4	0.7	0.5	0.5	0.02	13,137	-0.64
**Discount rate and willingness to pay**													
Discount 1%	-206	-55	-71	-0.3	0.0	0.0	0.4	0.7	0.5	0.5	0.02	4,464	-0.20
Discount 7%	-206	-55	-71	-0.3	0.0	0.0	0.4	0.7	0.5	0.5	0.01	3,737	-0.18
Willingness to pay £30,000	-206	-55	-71	-0.3	0.0	0.0	0.4	0.7	0.5	0.5	0.02	4,127	-0.12
													
	**Dementia**	**Myocardial infarction**	**Stroke**	**Dementia**	**Myocardial infarction**	**Stroke**	**Alive**	**Dementia**	**Myocardial infarction**	**Stroke**			
China													
Base case	-140	-25	-38	-0.2	0.0	0.0	0.3	0.5	0.3	0.3	0.01	1,697	-0.01
**Heterogeneity**													
Age 60 male	-79	-27	-39	-0.1	0.0	0.0	0.2	0.4	0.2	0.2	0.01	2,200	-0.02
Age 60 female	-84	-20	-29	-0.3	0.0	0.0	0.2	0.5	0.3	0.3	0.01	1,817	-0.01
Age 70 male	-256	-38	-64	-0.2	0.0	0.0	0.3	0.5	0.3	0.3	0.01	1,459	0.00
Age 70 female	-283	-25	-38	-0.4	0.0	0.0	0.4	0.8	0.4	0.4	0.02	666	0.01
**Time horizon**													
Up to age 90	-172	-32	-56	-0.3	0.0	0.0	0.2	0.5	0.2	0.3	0.01	1,665	-0.01
Up to age 80	-217	-43	-80	-0.2	0.0	-0.1	0.1	0.3	0.1	0.1	0.01	1,832	-0.02
**Disease and death risk methodology**													
LIBRA & QRISK3	-86	-42	-71	-0.2	0.0	-0.1	0.3	0.4	0.3	0.3	0.01	1,984	-0.01
No CVD history	-129	-23	-33	-0.3	0.0	-0.1	0.3	0.6	0.3	0.3	0.01	1,596	-0.01
No CVD history, LIBRA & QRISK3	-75	-41	-68	-0.2	-0.1	-0.1	0.3	0.5	0.3	0.4	0.01	1,915	-0.01
CAIDE score mean change	-94	-29	-48	-0.2	0.0	0.0	0.2	0.4	0.2	0.3	0.01	2,017	-0.02
Dementia-only	-148	0	0	-0.3	0.0	0.0	0.2	0.5	0.2	0.2	0.01	1,735	-0.01
Alternative disease incidence	-174	-21	-15	-0.4	0.0	0.0	0.3	0.7	0.3	0.3	0.01	1,223	0.00
Campbell RR	-140	-25	-38	-0.2	0.0	0.0	0.3	0.5	0.3	0.3	0.01	1,696	-0.01
Target population at increased risk	-189	-20	-27	-0.4	0.0	0.0	0.3	0.7	0.3	0.3	0.02	1,071	0.00
**Intervention effect, cost and duration**													
Adherent subgroup (dementia effect only using CAIDE score mean change)	-182	-21	-29	-0.3	0.0	0.0	0.3	0.6	0.3	0.3	0.01	1,412	0.00
Planned lifestyle change (dementia effect only)	-226	-17	-19	-0.4	0.0	0.0	0.3	0.7	0.4	0.4	0.02	1,116	0.00
China-specific effect (dementia effect only using CAIDE score mean change)	-128	-26	-40	-0.2	0.0	0.0	0.3	0.5	0.3	0.3	0.01	1,782	-0.01
Intervention repeated without coaching	-140	-25	-38	-0.2	0.0	0.0	0.3	0.5	0.3	0.3	0.01	-86	0.01
Lifetime full adherence with coaching	-1334	-103	-177	-1.1	0.0	0.0	1.3	2.4	1.3	1.3	0.04	-765	0.05
Lifetime full adherence without coaching	-1334	-103	-177	-1.1	0.0	0.0	1.3	2.4	1.3	1.3	0.04	-2,549	0.07
Costs x1/3	-140	-25	-38	-0.2	0.0	0.0	0.3	0.5	0.3	0.3	0.01	-253	0.01
Costs x3	-140	-25	-38	-0.2	0.0	0.0	0.3	0.5	0.3	0.3	0.01	7,547	-0.08
**Discount rate and willingness to pay**													
Discount 1%	-140	-25	-38	-0.2	0.0	0.0	0.3	0.5	0.3	0.3	0.02	1,634	0.00
Discount 7%	-140	-25	-38	-0.2	0.0	0.0	0.3	0.5	0.3	0.3	0.01	1,701	-0.01
Willingness to pay 3 times GDP	-140	-25	-38	-0.2	0.0	0.0	0.3	0.5	0.3	0.3	0.01	1,697	0.00

Abbreviations: CVD, cardiovascular disease; GDP, gross domestic product; RR, relative risk.

The use of LIBRA- instead of CAIDE-based relative risks for dementia showed smaller prevented onset of dementia and prevented person-time with dementia. The model outcomes were better in the subpopulation adherent to the intervention and those planning to make a lifestyle change within 6 months. The model outcomes were highly sensitive to the arbitrary assumption of full lifetime adherence.

In both the UK and China most scenarios potentially lacked cost-effectiveness (i.e., incremental net health benefit lower than zero).

## 4 Discussion

This health-economic study extrapolated the 12–18-months effects on risk factors observed in the PRODEMOS randomized trial (on a coach-supported mobile health intervention) to estimate the intervention’s potential effect on lifetime risk of developing dementia, MI and stroke, as well as the associated impact on QALYs and care costs, in a simulated cohort of 100,000 individuals aged 55 to 75 years with low SES in the UK, and any SES in China. Assuming causality between risk factors and disease onset, and a 10 % annual decline in adherence beyond the trial follow-up period of 12–18 months, the intervention may potentially lack cost-effectiveness in the UK and China. These results appeared to be mainly driven by the relatively small intervention effect on lifestyle changes, higher coaching time and salary costs in the UK and relatively low QALY gains in China.

The simulation model first translated the change in risk factors observed from the PRODEMOS trial to a relative risk of dementia, MI and stroke between the intervention and standard of care strategy. Assuming adherence beyond the trial follow-up period, it resulted in the prevention of dementia onset and MI and stroke events. Prevented disease corresponded to lower mortality risk, which rendered increased life expectancy. This generated a QALY gain, but also additional dementia onset, MI and stroke events in the life years saved and their corresponding care costs, as well as increased time living with disease. This explained the increased time with MI and stroke in some of the scenarios reported in Table 3.

### 4.1 Comparison to literature

Seven (systematic) reviews on dementia or Alzheimer’s disease health-economic evaluations [27,[34], [35], [36], [37], [38], [39]] identified nine simulation model studies assessing the primary prevention of dementia [[7], [8], [9], [10], [11], [12], [13], [14], [15]] through various interventions, targeted risk factors, effect magnitude and outcomes measures. Compared to models simulating effects of a prevention intervention on dementia and mortality [[7], [8], [9],^11^,^12^,^14^,^15^] our results similarly showed a reduction of time living with dementia, a higher life expectancy and QALYs gained. We identified additional studies without cost outcomes, which supported these results [40,41].

Among the identified studies, three evaluated a multi-domain intervention. Mukadam et al. [11] estimated the cost-effectiveness of a combined intervention involving antihypertensives treatment, smoking reduction and provision of hearing aids. In contrast to our findings, they reported a gain in QALY and reduction in costs and concluded that the intervention is worth implementing based on its effect on dementia alone, without considering effects on other conditions. These differences were likely driven by their assumption that intervention effects persisted until end of life and that their model starting at the start of the intervention only to those with the corresponding risk factors (e.g., reducing smoking only in those who smoke). Wimo et al. [14] estimated the cost-effectiveness of the Finnish Geriatric Intervention Study to Prevent Cognitive Impairment and Disability (FINGER), which had a similar effect on the CAIDE risk score as our PRODEMOS intervention. Compared to our UK results they reported about 8 times more prevented dementia cases, about two times as much QALYs gained, and cost savings rather than increased costs. These differences were likely driven by their assumption of 100 % intervention adherence over lifetime (compared to our 10 % annual non-adherence rate in our analysis) and substantially lower lifetime intervention costs of 5490 Swedish Krona (SEK) (approximately €500) compared to our £4505 (approximately €5360). Kato et al. [15] estimated the cost-effectiveness of combined physical and cognitive exercise among healthy adults based on observational evidence of its association with dementia. Compared to our results they reported gains in QALYs and a reduction in costs, although reporting was insufficiently clear to identify the potential drivers of these differences. From a methodological perspective, Youn et al. [42] highlighted the importance of modeling multiple diseases simultaneously particularly for assessing combined effects on utility but less for correlations between diseases, which was confirmed by our sensitivity analyses (**supplementary 6**, specifically table S6.1). This observation is further supported by Breeze et al. [12] who reported a relatively small effect of preventing CVD and diabetes on downstream effects on dementia. Lastly, similar to Breeze et al. [12] we found more cases of prevented dementia onset than prevented CVD events, although the numbers were small.

Compared to the Prevention of dementia by intensive vascular care (preDIVA) trial (with a relatively long follow-up of 7 years including dementia outcomes) reporting cumulative incidence of dementia (7 %) and MI (4 %), our UK standard of care strategy simulation showed 11 % dementia and 5 % MI cumulative incidence (using general population dementia incidence, preDIVA starting age and sex distribution, and the same 7-year time window). This could indicate an overestimation of dementia incidence by our model. Our model simulated less strokes prevented (−0.3 %) compared to FINGER (−1.5 %) 10-year registry follow-up [43], which could indicate an underestimation of cardiovascular benefits, although this was discussed as not being confirmed by other dementia lifestyle interventions [43].

Other multi-domain interventions [44] showed a difference in change from baseline between intervention and control arm in CAIDE score of −0.16 (Multidomain Alzheimer Preventive Trial (MAPT) [45]), ‑0.19 (preDIVA [46]), ‑0.11 (Healthy ageing through internet counselling in the elderly (HATICE) [47]), and −0.16 (FINGER [48]), as compared to −0.16 in the PRODEMOS study [6]. Post-hoc we performed a headroom analysis to estimate the annual intervention costs at which it would be considered cost-effective (i.e., at £20,000 and ¥81,419 per QALY) assuming the intervention is provided for a maximum of 10 years with 10 % annual non-adherence rate in the PRODEMOS target population and omitting an effect on MI and stroke. This resulted in a maximum annual intervention cost of £300-£514 and ¥916-¥1571 per year to be considered cost-effective, which limits long-term coach support.

### 4.2 Strengths and limitations

The model simulated effects beyond dementia on MI and stroke, it was externally validated within this study and is open-source available via https://github.com/ronhandels/prodemos, adding to methodology development and transparency.

The model outcomes showed a larger effect on dementia compared to MI and stroke. This could be explained by some CAIDE-based relative risks related to dementia being higher compared to the QRISK1-based relative risks related to CVD. A 1-point change in BMI corresponded to a relative risk of 0.98 for CVD, while it corresponded to a relative risk of 0.94 for dementia (similar to Breeze et al. [12]), reflecting a 2.9 times higher effect on dementia. Similarly, for hypertension it was 4.2 times higher (**supplementary material 6**, specifically 6.3). This could be a result of the CAIDE risk score based on a relatively small number of dementia cases (*n* = 61) [25] resulting in a relatively wide confidence interval or a result of being unadjusted for other factors such as smoking, leading to double counting when assuming causality. In comparison, a review by Livingston et al. [49] reported lower relative risks for hypertension (1.7 versus 2.1 based on CAIDE) and obesity (1.6 versus 2.17 based on CAIDE) and a review for the LIBRA risk model [32] reported a relative risk of 1.6 for both hypertension and obesity. In addition, the relative risk of hypertension on dementia from CAIDE was unadjusted for a history of stroke while with stroke also being a risk factor for dementia it likely resulted in double counting. Nevertheless, dementia has a higher incidence in high age compared to CVD (**supplementary 5**, specifically Figure S5.1) therewith having a large room for prevention.

Generalizability to care as usual is limited as the PRODEMOS trial included a convenience sample in China and had a relatively low response rate in the UK. Furthermore, standard of care (operationalized as disease incidence) differed between countries and was higher in China, leading to a higher room for disease prevention. In addition, lower labor costs in China led to lower coaching costs. Input estimates for our model were based on a variety of definitions for (recurrent) MI, stroke and dementia and settings (general population and clinical setting, and with and without low SES) when combining evidence on their incidence, relative risk, costs and utilities. In addition, western CAIDE and QRISK disease risk models were used for the Chinese setting. Its direction and magnitude are difficult to judge.

Costs in life years saved related to other diseases were not incorporated. Informal care costs related to stroke were not adjusted for being attributed only to stroke. Both overestimated the cost-effectiveness. However, the effect of reduced risk factors on other diseases (e.g., smoking on cancer) was not reflected, which underestimated the cost-effectiveness. Also, costs of new amyloid-targeting treatment for Alzheimer’s disease were not included, underestimating the costs of a subgroup of mild cognitive impairment and mild dementia eligible for treatment.

Probabilistic sensitivity analysis was not performed as plausible distributions were lacking for the assumption on causality and the assumption on lifetime intervention adherence. The omission of probabilistic analysis limits the representation of uncertainty and is therefore recommended to be addressed in future research.

### 4.3 Implications for practice, policy and research

The lack of evidence on true causality between risk factors and disease as well as absence of data on longer term sustained impact and adherence of interventions forced a variety of assumptions in our health-economic study. This led to a wide range of cost-effectiveness estimates. The clear gap between available and required evidence to obtain a robust cost-effectiveness estimate hindered recommendations on adopting the PRODEMOS intervention into standard of care. This suggests a need to address such gaps in evidence to avoid the uncertainty inherent in health-economic models.

Our study identified specific situations in which the PRODEMOS intervention may potentially be cost-effective. This involved scenarios with ad-hoc assumptions of long-term adherence with less frequent follow-up coaching and in subgroups with intended lifestyle changes. From a health-economic perspective to address the uncertainty related to the strong assumptions, this would require a long-term randomized study with repeated observation of adherence. For such extrapolation we also recommend improving dementia risk models; ideally using a multivariate survival model adjusting for demographic and other risk factors to prevent confounding, fitted to continuous outcomes (e.g., to distinguish a small from a large change crossing the hypertension threshold), include interactions with age (mid versus late life) and exposure duration. QALY and cost projections [50,51] could inform on optimal timing of intervention benefits. Our model is open-source and its simple model structure eases reproducibility and further development in future research.

## 5 Conclusion

The PRODEMOS coach-supported mobile health intervention for the primary prevention of dementia, aimed at people aged 55 to 75 years with low SES in the UK and those of any SES in China, may potentially lack cost-effectiveness in both countries. However, the results were based on strong assumptions regarding causality and sustained effectiveness, which limits policy recommendations.

## 6 Equations

Eq. (1): Economic evaluation outcomes.

(1) $$ICER=\frac{C_{i}-C_{c}}{E_{i}-E_{c}}$$

(2) $$iNHB=\left(E_{i}-E_{c}\right)-\frac{\left(C_{i}-C_{c}\right)}{\lambda }$$

ICER = incremental cost-effectiveness ratio
iNHB = incremental net health benefit
*C* = total cumulative costs
*E* = total cumulative effects (QALYs)
*i* = intervention strategy
*c* = standard of care strategy
λ = willingness to pay

## Ethical considerations

The PRODEMOS trial is sponsored in the UK by the University of Cambridge and is granted ethical approval by the London – Brighton & Sussex Research Ethics Committee (REC Reference: 20/LO/01440). In China the trial is approved by the Medical Ethics Committees of Capital Medical University (CMU), Beijing Tiantan Hospital affiliated to CMU, Beijing Geriatric Hospital, the Chinese People’s Liberation Army General Hospital, Taishan Medical University, and Xuanwu Hospital affiliated to CMU.

## Consent to participate

Not applicable

## Consent for publication

Not applicable

## Funding statement

This project has received funding from the European Union’s Horizon 2020 research and innovation programme under grant agreement No 779238 and the National Key R&D Programme of China (2017YFE0118800).

The funder had no role in the identification, design, conduct and reporting of the analysis.

## Declaration of the use of generative AI and AI-assisted technologies in scientific writing and in figures, images and artwork

During the preparation of this work the authors used Le Chat from Mistral AI in order to summarize information from earlier publications. After using this tool/service, the authors validated, reviewed and edited the content as needed and take full responsibility for the content of the published article.

## Data availability

The model and its corresponding data for an adjusted base case analysis are available via https://github.com/ronhandels/prodemos. Additional data are available upon reasonable request.

## CRediT authorship contribution statement

**Ron Handels:** Writing – review & editing, Writing – original draft, Validation, Methodology, Formal analysis, Conceptualization. **Marieke Hoevenaar-Blom:** Writing – review & editing, Validation, Formal analysis. **Manshu Song:** Writing – review & editing, Validation, Data curation. **Carol Brayne:** Writing – review & editing, Validation. **Eric Moll van Charante:** Writing – review & editing, Validation, Conceptualization. **Fiona E. Matthews:** Writing – review & editing, Validation. **Junfang Xu:** Writing – review & editing, Validation, Data curation. **Linus Jönsson:** Writing – review & editing, Validation, Methodology. **Nicola Coley:** Writing – review & editing, Validation. **Rachael Brooks:** Writing – review & editing. **Xuening Jian:** Writing – review & editing, Data curation. **Tingting Qin:** Writing – review & editing, Data curation. **Youxin Wang:** Writing – review & editing, Data curation. **Wei Wang:** Writing – review & editing, Validation. **Edo Richard:** Writing – review & editing, Validation, Conceptualization. **Anders Wimo:** Writing – review & editing, Validation, Methodology, Conceptualization.

## Declaration of interests

The authors declare the following financial interests/personal relationships which may be considered as potential competing interests:

Ron Handels reports a relationship with Lilly Nederland that includes: consulting or advisory. Ron Handels reports a relationship with Institute for Medical Technology Assessment that includes: consulting or advisory. If there are other authors, they declare that they have no known competing financial interests or personal relationships that could have appeared to influence the work reported in this paper.

## Appendix Supplementary materials

Image, application 1

Image, application 2
