# Supplementary material for: Prevention of dementia using mobile phone applications (PRODEMOS) – a health-economic cost-utility analysis in people aged 55–75 years with low socio-economic status
Source: J Prev Alzheimers Dis. 2026 Feb 27;13(5):100526. doi: 10.1016/j.tjpad.2026.100526 (PMC12966664; doi:10.1016/j.tjpad.2026.100526)
Supplement: Supplementary file 1 [file mmc1.pdf]

Supplementary material

Article title: Prevention of dementia using mobile phone applications (PRODEMOS) – a health-economic cost-utility analysis in people aged 55-75 years with low socio-economic status.

The Journal of Prevention of Alzheimer's Disease

Author names: Handels R, Hoevenaar-Blom M, Song M, Brayne C, Moll van Charante E, Matthews FE, Xu J, Jönsson L, Coley N, Brooks R, Jian X, Qin T, Wang Y, Wang W, Richard E, Wimo A, PRODEMOS study group.

Affiliation corresponding author: Maastricht University 6200MD, Maastricht, The Netherlands.

1 Supplementary material 1: PRODEMOS trial in- and exclusion criteria and trial details

Inclusion criteria:

- Age ≥ 55 years ≤ 75 years
- Living in a postal code area ranked as equal to or less than the lowest 3rd decile of IMD (applied only to participants in the UK) (deprivation)
- Good proficiency of the national language (English in UK, Mandarin in China)
- Possession of a smartphone
- ≥ Two Dementia risk factors:
  - Insufficient physical activity (self-reported intermediate or vigorous activity of < 150 minutes per week)
  - Active smoking (self-reported use of any sort of tobacco in any quantity)
  - Depression:
    - Current diagnosis by specialist or GP or;
    - History of treatment for depression (i.e., drug therapy or psychotherapy)
  - Manifest cardiovascular disease, as diagnosed by specialist or GP
  - Diabetes mellitus:
    - Diagnosed by specialist or GP or;
    - Use of insulin or other blood glucose-lowering medication
  - Hypertension:
    - Diagnosed by specialist or GP or;
    - Use of blood pressure-lowering medication or;
    - Mean of baseline blood pressure measurements of ≥ 140 (systolic) or ≥ 90 (diastolic)
  - Overweight:
    - BMI ≥ 30 (UK), ≥ 28 (China) or;
    - Baseline waist circumference ≥ 102 cm (men in UK), 90 cm (men in China), 88 cm (women in UK), 85 cm (women in China)
  - Dyslipidemia:
    - Diagnosed by specialist or GP or;
    - Use of lipid-lowering medication or;
    - Baseline total cholesterol ≥ 5.0 mmol/L (applied only to participants in the UK)

Exclusion criteria:

- Manifest dementia, as diagnosed by specialist or GP
- MMSE < 24 (participants with ISCED level of > 1), MMSE <21 (participants with ISCED level of 1)
- Any condition expected to limit 18-months follow-up, including metastasized malignancy or other terminal illnesses
- Smartphone illiteracy, defined as not being able to send a message from a smartphone
- Visual impairment interfering with operation of a smartphone
- Participating in another RCT on behavior change

- Present severe alcohol or illicit drug abuse

Copied from Eggink et al. [2021 https://doi.org/10.1136/bmjopen-2021-049762] with permission from the authors.

Trial details:

- Effectiveness and implementation [Moll van Charante, 2024]
- Trial design [Eggink, 2021]
- Registry including pre-defined analyses plan for effectiveness, implementation and cost-effectiveness [https://doi.org/10.1186/ISRCTN15986016]

1.1 References

- Eggink et al. (2021) Prevention of dementia using mobile phone applications (PRODEMOS): protocol for an international randomised controlled trial. BMJ Open 2021;11:e049762. doi:10.1136/bmjopen-2021-049762
- Moll van Charante EP, Hoevenaar-Blom MP, Song M, Andrieu S, Barnes L, Birck C, Brooks R, Coley N, Eggink E, Georges J, Hafdi M, van Gool WA, Handels R, Hou H, Lyu J, Niu Y, Song L, Wang W, Wang Y, Wimo A, Yu Y, Zhang J, Zhang W, Brayne C, Wang W, Richard E; PRODEMOS study group. Prevention of dementia using mobile phone applications (PRODEMOS): a multinational, randomised, controlled effectiveness-implementation trial. Lancet Healthy Longev. 2024 Jun;5(6):e431-e442. doi: 10.1016/S2666-7568(24)00068-0. Epub 2024 May 16. PMID: 38763155.

2 Supplementary material 2: Prevalence, relative risks and mapping of risk factors

TABLE S2-1: PREVALENCE AND RELATIVE RISK OF FACTORS USED TO MODIFY THE RISK FOR DEMENTIA AND CVD.

Factor	RR for dementia (UK and China)	RR for MI and stroke (UK and China)	Prevalence or mean & SD in UK (from CFASII or alternative source reflecting the general population as targeted by CFASII study)	Prevalence or mean & SD in China
Education (years)	Source: CAIDE [Kivipelto, 2006]. Estimate: **1 = ≥10 (reference). 2.33 = 7-9. 3.24 = 0-6.** Adjusted for: all CAIDE model factors (age, sex, education, hypertension, obesity, hypercholesterolaemia, physical inactivity). Comment: based on OR (2.485 and 3.599) transformed to RR [Gidwani, 2020] Equation S1 with prevalence = 61/(1348+61) = 0.043 obtained from [Kivipelto, 2006: table 1].	Not available in CVD risk equation [Damen, 2016].	Source: CFASII database. Estimate: **0.71 = ≥10. 0.28 = 7-9. 0.01 = 0-6.** Comment: proportion; sampling weight applied.	Source: [Wang,2017: table 1]. Estimate: **10.3% + 0.1% = >10 years (assumed category for 'college or higher'). 54.1% + 0.2% = 7-9 years (assumed category for 'middle school). 35.1% + 0.2% = 0-6 years (assumed category for 'primary school or lower').** Comment: proportion; classification judged in terms of years according to the CAIDE classification. Missing data were distributed over the categories to ensure percentages add up to 100.
Deprivation (Townsend score)	Source: CFASII [Bennett, 2018; table 4.6]. Estimate: **1 = least deprived (reference). 1.4 = mid-level deprived. 1.5 = most deprived.** Adjusted for: age, sex. Comment: incidence rate ratio; deprivation levels based on Townsend deprivation index on post code split into tertiles.	Source: QRISK1 [Hippisley-Cox, 2007]. Estimate: **1.017 for men. 1.035 for women.** Adjusted for: all QRISK 1 factors (age, family history, Townsend deprivation index, cholesterol, BMI, smoking, SBP, anti-hypertension treatment). Comment: HR for postcode-related Townsend score of output area. Output area level 2001 census data evaluated as a continuous variable.	Source: definition. Estimate: Proportion: **0.333 = least deprived. 0.333 = mid-level deprived. 0.334 = most deprived. Mean = 0.** Comment: proportion; tertiles by definition 1/3 each, and mean of continuous value by definition mean = 0 and SD = 4 [Yousaf, 2017]. Tertile cut points are -2.26 and 0.29 (established before sampling weight was applied); score ranges between −6 (most affluent) and 11 (most deprived).	n/a (low socio-economic status represented by population, see details in manuscript)
Type 2 diabetes mellitus	Source: CFASII [Bennett, 2018; table 4.7]. Estimate:	Source: QRISK2 [Hippisley-Cox, 2008]. Estimate: **1.6 for men.**	Source: CFASII database. Estimate: **0.14.**	Source: [Wang, 2021] Estimate: **0.124 = proportion.** Comment: none.

	**1.0.**<br>Adjusted for: age and sex.<br>Comment: RR; self-reported diabetes).	**2.0 for women.**<br>Adjusted for: all QRISK2 model factors (age, family history, Townsend deprivation index, cholesterol, BMI, smoking, SBP, anti-hypertension treatment).<br>Comment: HR for type 2 diabetes at age 65; estimates read from figure 1 because reported estimates in table 5 (2.54 for women, 2.20 for men) included interaction with age, which was not implemented for simplification.	Comment: proportion; self-reported type 2 diabetes; sampling weight applied.	
Depression	Source: CFASII [Bennett, 2018; table 4.7].<br>Estimate:<br>**1.1.**<br>Adjusted for: age and sex.<br>Comment: RR, self-reported depression.	Not available in CVD risk equation [Damen, 2016].	Source: CFASII database.<br>Estimate:<br>**0.067.**<br>Comment: proportion; depression based on CFASII algorithm including self-reported symptoms of depression; sampling weight applied.	Source: [Lu, 2021: table 2].<br>Estimate:<br>**0.038 = proportion**.<br>Comment: prevalence of any depressive disorder over 12-month period in age ≥65.
SBP / hypertension	Source: CAIDE [Kivipelto, 2006].<br>Estimate:<br>**2.10.**<br>Adjusted for: all CAIDE model factors (age, sex, education, hypertension, obesity, hypercholesterolaemia, physical inactivity).<br>Comment: based on OR (2.206 for >140 mm HG) transformed to RR [Gidwani, 2020] Equation S1 with prevalence = 61/(1348+61) = 0.043 obtained from [Kivipelto, 2006: table 1].	Source: QRISK1 [Hippisley-Cox, 2007].<br>Estimate:<br>**1.004 for men.**<br>**1.005 for women.**<br>Adjusted for: all QRISK1 model factors (age, family history, Townsend deprivation index, cholesterol, BMI, smoking, SBP, anti-hypertension treatment).<br>Comment: HR per 1-unit increase in systolic blood pressure mm Hg.	Source: HSE [Falaschetti, 2014: table 1].<br>Estimate:<br>**135 = mean.**<br>**17.6 = SD.**<br>**0.39 = corresponding proportion.**<br>Comment: mmHg; weighted from age specific estimates; proportion above 140 obtained from value of 140 from density function of normal distribution based on mean and SD (which was 0.39).	Source: [Zhang, 2021].<br>Estimate:<br>**127.7 = mean.**<br>**18.8 = SD.**<br>**0.275 = proportion.**<br>Comment: none.
BMI / obesity	Source: CAIDE [Kivipelto, 2006].<br>Estimate:<br>**2.17.**<br>Adjusted for: all CAIDE model factors (age, sex, education, hypertension, obesity, hypercholesterolaemia, physical inactivity).<br>Comment: based on OR (2.296 for >30 kg/m^2)	Source: QRISK1 [Hippisley-Cox, 2007].<br>Estimate:<br>**1.022 for men.**<br>**1.015 for women.**<br>Adjusted for: all QRISK1 model factors (age, family history, Townsend deprivation index, cholesterol, BMI, smoking, SBP, anti-	Source: [HSE: Overweight and obesity in adults and children data tables, HSE18-Adult-Child-Obesity-tab.xlsx > table 3].<br>Estimate:<br>**28.2 = mean.**<br>**4.6 = SD.**<br>**0.35 = corresponding proportion**	Source: [Chen, 2023].<br>Estimate:<br>**24.1 = mean.**<br>**14.1 = proportion.**<br>Comment: obesity defined as ≥28 kg/m^2.

	transformed to RR [Gidwani, 2020] Equation S1 with prevalence = 61/(1348+61) = 0.043 obtained from [Kivipelto, 2006: table 1].	hypertension treatment). Comment: HR for body mass index kg/m2.	Comment: kg/m^2; weighted from age- and sex specific estimates; proportion above 30 obtained from value of 30 from density function of normal distribution based on mean and SD (which was 0.35).	
Cholesterol total / hyper-choleste-rolaemia	Source: CAIDE [Kivipelto, 2006]. Estimate: **1.81.** Adjusted for: all CAIDE model factors (age, sex, education, hypertension, obesity, hypercholesterolaemia, physical inactivity). Comment: based on OR (1.879 for total cholesterol >6.5 mmol/L) transformed to RR [Gidwani, 2020] Equation S1 with prevalence = 61/(1348+61) = 0.043 based on [Kivipelto, 2006: table 1].	n/a (not available for total cholesterol; only ratio HDL/total)	Source: HSE [Hippisley-Cox, 2007: figure]. Estimate: **6 = mean.** **1.2 = SD.** **0.34 = corresponding proportion.** Comment: read from graph at age 65-74; mean from women and men; SD assumed similar to CAIDE [Kivipelto, 2006: table 1] being 1.2 (weighted mean from those who did not develop dementia and those who developed dementia); proportion above 6.5 obtained from value of 6.5 from density function of normal distribution based on mean and SD (which was 0.34).	Source: [Xia, 2023]. Estimate: **0.083 = proportion.** Comment: none.
Physical inactivity	Source: CAIDE [Kivipelto, 2006]. Estimate: **1.64.** Adjusted for: all CAIDE model factors (age, sex, education, hypertension, obesity, hypercholesterolaemia, physical inactivity). Comment: based on OR (1.693 for inactive defined as those who engaged in physical activity at least twice a week, lasting at least 20–30 min each time, and causing sweating and breathlessness, were regarded as active, and others as inactive) transformed to RR [Gidwani, 2020] Equation S1 with prevalence = 61/(1348+61) = 0.043 based on [Kivipelto, 2006: table 1].	Not implemented	Source: CFASII database. Estimate: **0.25.** Comment: proportion, inactive defined as not taking part in any moderate or vigorous physical activity; sampling weight applied.	Source: [Zhang, 2023: table 2]. Estimate: **0.2185 = proportion.** Comment: mean of age group 50-64 (18.2%) and ≥65 (25.5%) in 2018; definition is moderate to vigorous physical activity < 150min/w.

Smoking	Source: CFASII [Bennett, 2018; table 4.8]. Estimate: **1 = never (reference). 1.1 = past. 1.7 = current**. Adjusted for: age and sex. Comment: RR.	Source: QRISK1 [Hippisley-Cox, 2007]. Estimate: **1.417 for men. 1.530 for women.** Adjusted for: all QRISK1 model factors (age, family history, Townsend deprivation index, cholesterol, BMI, smoking, SBP, anti-hypertension treatment). Comment: HR for smoking status being current smoker.	Source: CFASII database. Estimate: **0.38 = never. 0.51 = past. 0.11 = current.** Comment: proportion; sampling weight applied.	Source: [Wang, 2017: table 3] Estimate: **0.496 = current.** Comment: proportion; smoking: 47.63% was current smoker, 48.34% was not, 4.01% was missing [Wang, 2017: table 3]. Adjusted for missing, the proportion current smoker is 47.63/(47.63+48.34)=0.496

Equation S1: Calculate relative risk from odd's ratio (obtained from Gidwani et al. [2020]).

$$RR = \frac{OR}{\left(1 - p + (p * OR)\right)}$$

$RR = relative\ risk$

$OR = odd's\ ratio$

$p = prevalence$

2.1 References

- Bennett (2018) Dementia risk in the population over time: potential for primary prevention and intervention. PhD thesis.
- Chen K, Shen Z, Gu W, Lyu Z, Qi X, Mu Y, Ning Y; Meinian Investigator Group. Prevalence of obesity and associated complications in China: A cross-sectional, real-world study in 15.8 million adults. Diabetes Obes Metab. 2023 Nov;25(11):3390-3399. doi: 10.1111/dom.15238. Epub 2023 Aug 17. PMID: 37589256.
- Damen JA, Hooft L, Schuit E, Debray TP, Collins GS, Tzoulaki I, Lassale CM, Siontis GC, Chiocchia V, Roberts C, Schlüssel MM, Gerry S, Black JA, Heus P, van der Schouw YT, Peelen LM, Moons KG. Prediction models for cardiovascular disease risk in the general population: systematic review. BMJ. 2016 May 16;353:i2416. doi: 10.1136/bmj.i2416. PMID: 27184143; PMCID: PMC4868251.
- Falaschetti E, Mindell J, Knott C, Poulter N. Hypertension management in England: a serial cross-sectional study from 1994 to 2011. Lancet. 2014 May 31;383(9932):1912-9. doi: 10.1016/S0140-6736(14)60688-7. PMID: 24881995.
- Gidwani R, Russell LB. Estimating Transition Probabilities from Published Evidence: A Tutorial for Decision Modelers. Pharmacoeconomics. 2020 Nov;38(11):1153-1164. doi: 10.1007/s40273-020-00937-z. Erratum in: Pharmacoeconomics. 2020 Sep 8;: PMID: 32797380; PMCID: PMC7426391.
- Hippisley-Cox J, Coupland C, Brindle P. Development and validation of QRISK3 risk prediction algorithms to estimate future risk of cardiovascular disease: prospective cohort study. BMJ. 2017 May 23;357:j2099. doi: 10.1136/bmj.j2099.
- HSE. Health Survey England. https://digital.nhs.uk/
- Kivipelto M, Ngandu T, Laatikainen T, Winblad B, Soininen H, Tuomilehto J. Risk score for the prediction of dementia risk in 20 years among middle aged people: a longitudinal, population-based study. Lancet Neurol. 2006 Sep;5(9):735-41. doi: 10.1016/S1474-4422(06)70537-3. PMID: 16914401.
- Lu J, Xu X, Huang Y, Li T, Ma C, Xu G, Yin H, Xu X, Ma Y, Wang L, Huang Z, Yan Y, Wang B, Xiao S, Zhou L, Li L, Zhang Y, Chen H, Zhang T, Yan J, Ding H, Yu Y, Kou C, Shen Z, Jiang L, Wang Z, Sun X, Xu Y, He Y, Guo W, Jiang L, Li S, Pan W, Wu Y, Li G, Jia F, Shi J, Shen Z, Zhang N. Prevalence of depressive disorders and treatment in China: a cross-sectional epidemiological study. Lancet Psychiatry. 2021 Nov;8(11):981-990. doi: 10.1016/S2215-0366(21)00251-0. Epub 2021 Sep 21. PMID: 34559991.
- Wang W, Jiang B, Sun H, Ru X, Sun D, Wang L, Wang L, Jiang Y, Li Y, Wang Y, Chen Z, Wu S, Zhang Y, Wang D, Wang Y, Feigin VL; NESS-China Investigators. Prevalence, Incidence, and Mortality of Stroke in China: Results from a Nationwide Population-Based Survey of 480 687 Adults. Circulation. 2017 Feb 21;135(8):759-771. doi: 10.1161/CIRCULATIONAHA.116.025250. Epub 2017 Jan 4. PMID: 28052979.
- Wang L, Peng W, Zhao Z, Zhang M, Shi Z, Song Z, Zhang X, Li C, Huang Z, Sun X, Wang L, Zhou M, Wu J, Wang Y. Prevalence and Treatment of Diabetes in China, 2013-2018. JAMA. 2021 Dec 28;326(24):2498-2506. doi: 10.1001/jama.2021.22208. Erratum in: JAMA. 2022 Mar 15;327(11):1093. PMID: 34962526; PMCID: PMC8715349.

- Xia Q, Chen Y, Yu Z, Huang Z, Yang Y, Mao A, Qiu W. Prevalence, awareness, treatment, and control of dyslipidemia in Chinese adults: a systematic review and meta-analysis. Front Cardiovasc Med. 2023 Jul 5;10:1186330. doi: 10.3389/fcvm.2023.1186330. PMID: 37476570; PMCID: PMC10354280.
- Yousaf S, Bonsall A (2011) UK Townsend Deprivation Scores from 2011 census data. https://s3-eu-west-1.amazonaws.com/statistics.digitalresources.jisc.ac.uk/dkan/files/Townsend_Deprivation_Scores/UK%20Townsend%20Deprivation%20Scores%20from%202011%20census%20data.pdf
- Zhang M, Wu J, Zhang X, Hu CH, Zhao ZP, Li C, Huang ZJ, Zhou MG, Wang LM. [Prevalence and control of hypertension in adults in China, 2018]. Zhonghua Liu Xing Bing Xue Za Zhi. 2021 Oct 10;42(10):1780-1789. Chinese. doi: 10.3760/cma.j.cn112338-20210508-00379. PMID: 34814612.
- Zhang M, Ma Y, Xie X, Sun M, Huang Z, Zhao Z, Zhang X, Li C, Gao X, Wu J, Wang L, Zhou M, Wen D. Trends in insufficient physical activity among adults in China 2010-18: a population-based study. Int J Behav Nutr Phys Act. 2023 Jul 17;20(1):87. doi: 10.1186/s12966-023-01470-w. PMID: 37460936; PMCID: PMC10351178.

3 Supplementary material 3: Model analytics

The model was built up as follows:

1. General population incidence for each disease (dementia, MI and stroke) specific for age, sex and disease-history (dementia, MI and stroke) was predicted from a fitted regression model on the general population-based CFASII data for UK and literature for China (see supplementary material 3.1). Available rates from the model by Campbell et al. [2015] were not used as they were unspecific for disease history and would require calibration to make them conditional for history of other diseases.
2. General population age, sex and disease-history specific mortality rate was obtained by multiplying a UK- and China-specific life table (see supplementary material 3.2.1) with the relative risk obtained from a fitted regression model on general population-based CFASII data (UK) or literature (China) (see supplementary material 3.2.2). The mortality rate was adjusted to reflect those without a history of the 3 diseases to prevent double counting the disease-related mortality included in the life table (see supplementary material 3.2.3).
3. The starting population was reflected by combinations of age band and sex, and their corresponding prevalence of history of MI and history of stroke in the PRODEMOS baseline sample (see supplementary material 3.3.1). In addition, disease rates were adjusted to reflect the PRODEMOS target population at increased risk of dementia (see supplementary material 3.3.2). The RR of developing the diseases in the PRODEMOS target population versus its reference (i.e., general population) was estimated by raising the RRs from published sources (mainly CAIDE and QRISK) to the difference in risk factors prevalence or mean between the target (i.e., PRODEMOS target population) and its reference (i.e., general population).
4. The intervention effect (lower risk factor status) was implemented as a relative risk compared to standard of care for developing each of the 3 diseases (see supplementary material 3.4), using the same method as for reflecting the PRODEMOS target population in the previous step. The same RRs were raised to the difference in risk factors between the target population (i.e., intervention strategy) and its reference population (i.e., standard of care strategy), adjusted for effect waning.
5. All rates were converted to transition probabilities (see supplementary material 3.5). Mortality was applied first, after which transitions between diseases took place (i.e. disease transitions were conditional on survival). The Markov model was run multiple times per combination of starting age and sex, and results were weighted proportionally to their prevalence in the PRODEMOS study at baseline, separately for UK and China.
6. Person-years living with a history of the disease and (recurrent) events were multiplied with quality of life based utility and cost estimates (see supplementary material 3.6) to obtain lifetime quality-adjusted life years (QALY) and total costs.

3.1 General population disease incidence rate by age, sex and history of disease

For the UK the general population incidence rates for dementia onset event, myocardial infarction event and stroke event were estimated using data from the second Cognitive Function and Ageing Study (CFAS-II).

This population-based study aimed to investigate dementia and cognitive decline. It recruited among the general population a sample of persons aged 65 or older (2008-2011) and obtained a 2-year follow-up assessment (2011-2013), in the geographical areas Cambridgeshire, Newcastle and Nottingham of the UK. The dementia syndrome (any type) was established based on an algorithmic approach called GSM-AGECAT, which was validated against internationally accepted diagnostic criteria [Matthews, 2016]. Heart attack was assessed in an interview asking whether a participant had or suffered from a heart attack and who diagnosed this (no doctor, GP, specialist) "ever" on baseline or "since the baseline visit" on follow-up. The same was asked for stroke, for which the interviewee was also instructed to "record only episodes that lasted for 24 hours or longer with partial paralysis in left or right arm and/or leg, blindness in eye/s, or speech disturbance". In Matthews et al. [2016] inverse probability weighting methods were used that adjusted for nonresponse, and prevalence estimates were standardized by the UK age and sex distribution at the time of the first interviews; which was not applied in our analyses.

Data availability, selection and imputation:

- Participants who were lost to follow-up (20% refused and 4% other reason) were omitted from analysis, an attrition weight was used to adjust for this.
- On the remaining data (see Table S3-1), participants who had missing data on status of history of dementia (<1%, <1%), heart attack (3%, 3%) or stroke (3%, 3%) at baseline and follow-up for the selection of persons who survived respectively were assumed to not have experienced the disease event. These were manually imputed as no event.
- For the analysis on dementia onset event, those with a history of dementia at baseline were omitted from analysis as dementia was considered an irreversible status rather than a (possibly recurrent) event. Before manual imputation, 3 participants had dementia at baseline and not at follow-up; after manual imputation this was 4.
- For the analysis the data of age groups 90 year or older were omitted as they were considered too few for a face valid model fit.
- For the analysis the data of those who were dead at 2-year follow-up were omitted.

Table S3-1: CFAS-II data (after omitting lost to follow-up) on death, and on follow-up status of dementia, heart attack since baseline and stroke since baseline for those alive at follow-up.

Age band	Total	Died	Dementia (at baseline)	Dementia missing	Heart attack	Heart attack status missing	Stroke	Stroke status missing
Male								
65-69	767	29	8 (0)	0	8	1	10	1
70-74	720	47	22 (10)	1	8	7	11	8
75-79	583	52	29 (12)	0	9	11	12	13
80-84	421	74	33 (16)	0	9	12	6	13
85-89	215	58	18 (6)	8	1	17	5	17
90-94	58	26	4 (2)	0	1	3	0	3
95-99	11	8	0 (0)	0	0	1	0	1
100+	1	0	0 (0)	0	0	0	0	0
Female								
65-69	763	23	9 (2)	0	6	3	9	5
70-74	724	28	12 (3)	0	7	8	11	9
75-79	635	44	32 (14)	0	12	24	10	26
80-84	542	86	50 (13)	0	7	22	10	26
85-89	327	85	33 (16)	14	6	31	9	32
90-94	126	56	18 (8)	0	2	11	2	11
95-99	35	25	6 (3)	0	0	2	0	2
100+	2	2	0 (0)	0	0	0	0	0

Three general linear models (GLM) were fit, to:

- dementia onset event during 2-year follow-up
- heart attack event during 2-year follow-up
- stroke event during 2-year follow-up

The following subsample was used:

- alive at follow-up
- age 65-89 at baseline
- no dementia at baseline (applied only to the model with dementia onset as dependent variable)

The following independent variables were included in the model using stepwise backward using p-value 0.05 to keep (except for those forced entry):

- baseline age (continuous) (forced entry)
- baseline sex (forced entry)
- baseline interaction between age and sex
- baseline history of dementia (only for the models with heart attack and stroke event as dependent variable)
- baseline history of heart attack
- baseline history of stroke

The following model was used:

- family Poisson
- link log

- Exposure was assumed 2 years (full follow-up period) for those who did not experience the event and 1 year for those who did experience the event (i.e., assumed half-way during the follow-up period)

The following weights were applied:

- Weights to adjust for sampling to represent the general population
- Weights to adjust for attrition from baseline to 2-year follow-up in those alive at follow-up
- For details, see Matthews et al. [2013]

See Table S3-2 for the results.

TABLE S3-2: RESULTS OF THE WEIGHTED REGRESSION ANALYSIS FOR INCIDENCE OF DEMENTIA ONSET EVENT, HEART ATTACK EVENT AND STROKE EVENT (TOP TO BOTTOM) DURING 2-YEAR FOLLOW-UP IN CFAS-II DATA.

```
.        glm dementia_w2 c.age_w1 sex_f                     stroke_w1 if audit_w2==1 & age_w
> 1<=89 & dementia_w1!=1 [pweight=static_weight_v3*attrition_weight_w2], fam(poisson) link(log
> ) exposure(exposure_dem) nolog vce(robust)

Generalized linear models                          No. of obs      =      5,079
Optimization     : ML                              Residual df     =      5,075
                                                   Scale parameter =          1
Deviance         =  3336.599043                    (1/df) Deviance =   .6574579
Pearson          =  21973.31153                    (1/df) Pearson  =   4.329717

Variance function: V(u) = u                        [Poisson]
Link function    : g(u) = ln(u)                    [Log]

                                                   AIC             =   .8374632
Log pseudolikelihood = -2122.737884                BIC             =  -39967.71

------------------------------------------------------------------------------
             |               Robust
 dementia_w2 |      Coef.   Std. Err.      z    P>|z|     [95% Conf. Interval]
-------------+----------------------------------------------------------------
      age_w1 |   .1076994   .0117963     9.13   0.000     .0845791    .1308197
       sex_f |   .1582646   .1681928     0.94   0.347    -.1713871    .4879164
   stroke_w1 |   .5373188   .2477953     2.17   0.030     .0516489    1.022989
       _cons |  -12.39739   .9246474   -13.41   0.000    -14.20966   -10.58511
ln(exposu~m) |          1  (exposure)
------------------------------------------------------------------------------
```

```
.         glm mi_w2 c.age_w1 sex_f                            mi_w1           if audit_w2==1 &
>  age_w1<=89 [pweight=static_weight_v3*attrition_weight_w2], fam(poisson) link(log) exposure(
> exposure_chd) nolog vce(robust)

Generalized linear models                         No. of obs      =      5,171
Optimization     : ML                             Residual df     =      5,167
                                                  Scale parameter =          1
Deviance         =  1682.345965                   (1/df) Deviance =   .3255943
Pearson          =  24584.48211                   (1/df) Pearson  =    4.75798

Variance function: V(u) = u                       [Poisson]
Link function    : g(u) = ln(u)                   [Log]

                                                  AIC             =   .3998583
Log pseudolikelihood = -1029.833713               BIC             =  -42499.75

------------------------------------------------------------------------------
             |               Robust
       mi_w2 |      Coef.   Std. Err.      z    P>|z|     [95% Conf. Interval]
-------------+----------------------------------------------------------------
      age_w1 |   .0253612   .0181277     1.40   0.162    -.0101684    .0608907
       sex_f |   .3890458   .2418751     1.61   0.108    -.0850206    .8631122
       mi_w1 |   2.027214    .247567     8.19   0.000     1.541992    2.512437
       _cons |  -7.498702   1.393916    -5.38   0.000    -10.23073   -4.766676
ln(exposu~d) |          1  (exposure)
------------------------------------------------------------------------------
```

```
.         glm stroke_w2 c.age_w1 sex_f                            mi_w1 stroke_w1 if audit_w2=
> =1 & age_w1<=89 [pweight=static_weight_v3*attrition_weight_w2], fam(poisson) link(log) expos
> ure(exposure_str) nolog vce(robust)

Generalized linear models                         No. of obs      =      5,171
Optimization     : ML                             Residual df     =      5,166
                                                  Scale parameter =          1
Deviance         =  2096.550194                   (1/df) Deviance =   .4058363
Pearson          =  25115.60913                   (1/df) Pearson  =   4.861713

Variance function: V(u) = u                       [Poisson]
Link function    : g(u) = ln(u)                   [Log]

                                                  AIC             =   .4997925
Log pseudolikelihood = -1287.213497               BIC             =  -42076.99

------------------------------------------------------------------------------
             |               Robust
   stroke_w2 |      Coef.   Std. Err.      z    P>|z|     [95% Conf. Interval]
-------------+----------------------------------------------------------------
      age_w1 |   .0229375   .0173827     1.32   0.187    -.0111319     .057007
       sex_f |   .1996352   .2357979     0.85   0.397    -.2625202    .6617906
       mi_w1 |    .748028   .3060613     2.44   0.015      .148159    1.347897
   stroke_w1 |   1.507333   .2472286     6.10   0.000     1.022774    1.991892
       _cons |  -6.838972   1.299249    -5.26   0.000    -9.385453   -4.292491
ln(exposu~r) |          1  (exposure)
------------------------------------------------------------------------------
```

age_w1 = age at baseline; sex_f = female sex; dementia_w1 = history of dementia onset at baseline; mi_w1 = history of MI at baseline; stroke_w1 = history of stroke at baseline. # represents interaction term; dementia_w2 = dementia at 2-year follow-up; mi_w2 = MI at 2-year follow-up; stroke_w2 = stroke at 2-year follow-up.

The face validity of the model was judged in 2 ways.

First, the unweighted observed rate was compared to the unweighted and weighted predicted rate using a simplified model without heart attack and stroke as predictors, using the same method for calculating exposure time for observed rate as indicated earlier. The confidence interval of the observed rate was estimated using Equation S2. It was considered valid based on visual inspection of the plot, see Figure S3-1.

EQUATION S2: LOWER AND UPPER BOUND OF THE 95% CONFIDENCE INTERVAL OF AN OBSERVED RATE.

$$lower\ bound = e^{ln(r)-1.96SE} \quad (1)$$

$$upper\ bound = e^{ln(r)+1.96SE} \quad (2)$$

$$SE = \sqrt{\frac{1-r}{n}} \quad (3)$$

r = rate

SE = standard error

n = number of events

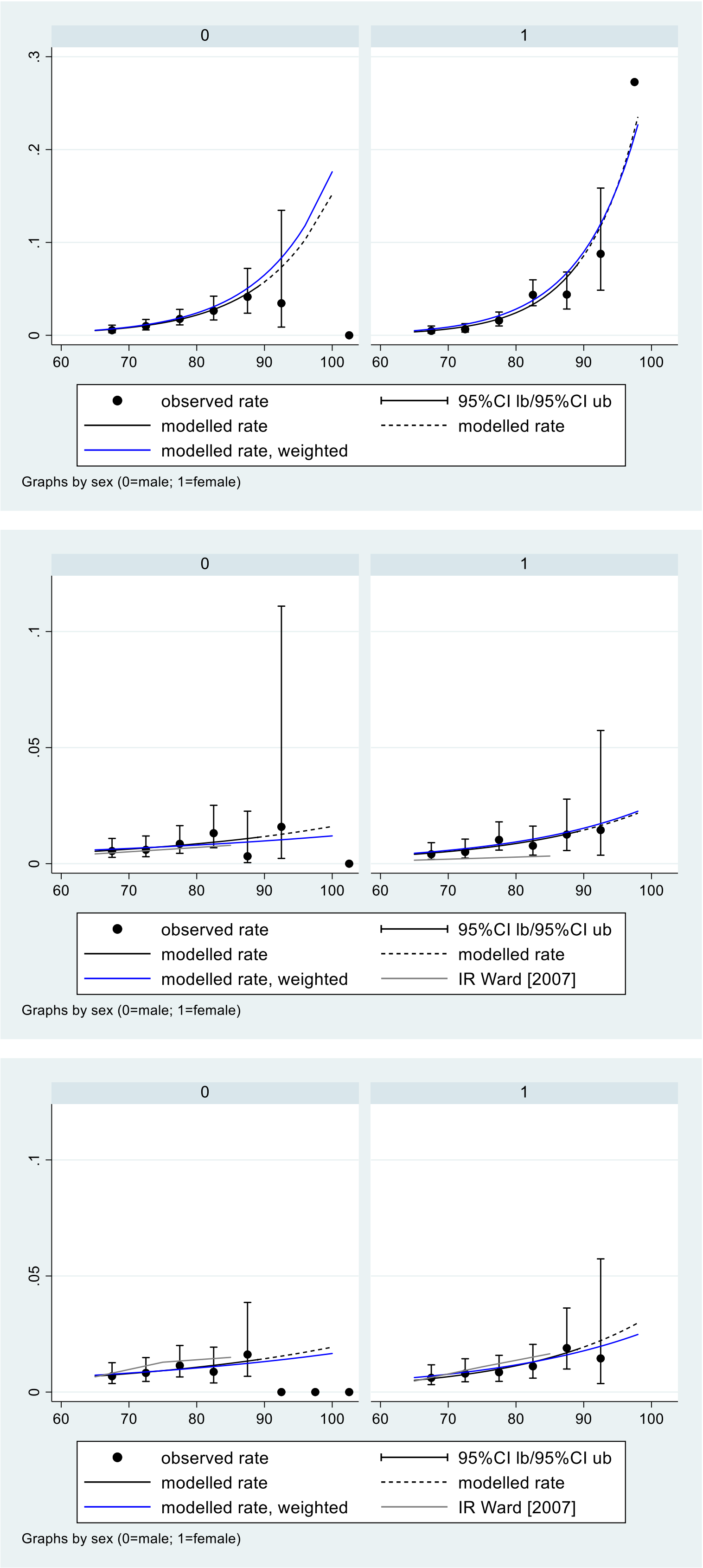

FIGURE S3-1: OBSERVED AND PREDICTED INCIDENCE RATE FOR DEMENTIA ONSET EVENT, HEART ATTACK EVENT AND STROKE EVENT (TOP TO BOTTOM). DASHED = PREDICTION OUTSIDE AGE RANGE OF THE DATA USED TO FIT THE MODEL.

Second, the unweighted observed rate (and confidence interval) was compared to the mean unweighted predicted rate, by deciles of the predicted rate. It was considered valid based on visual inspection of the plot, see Figure S3-2.

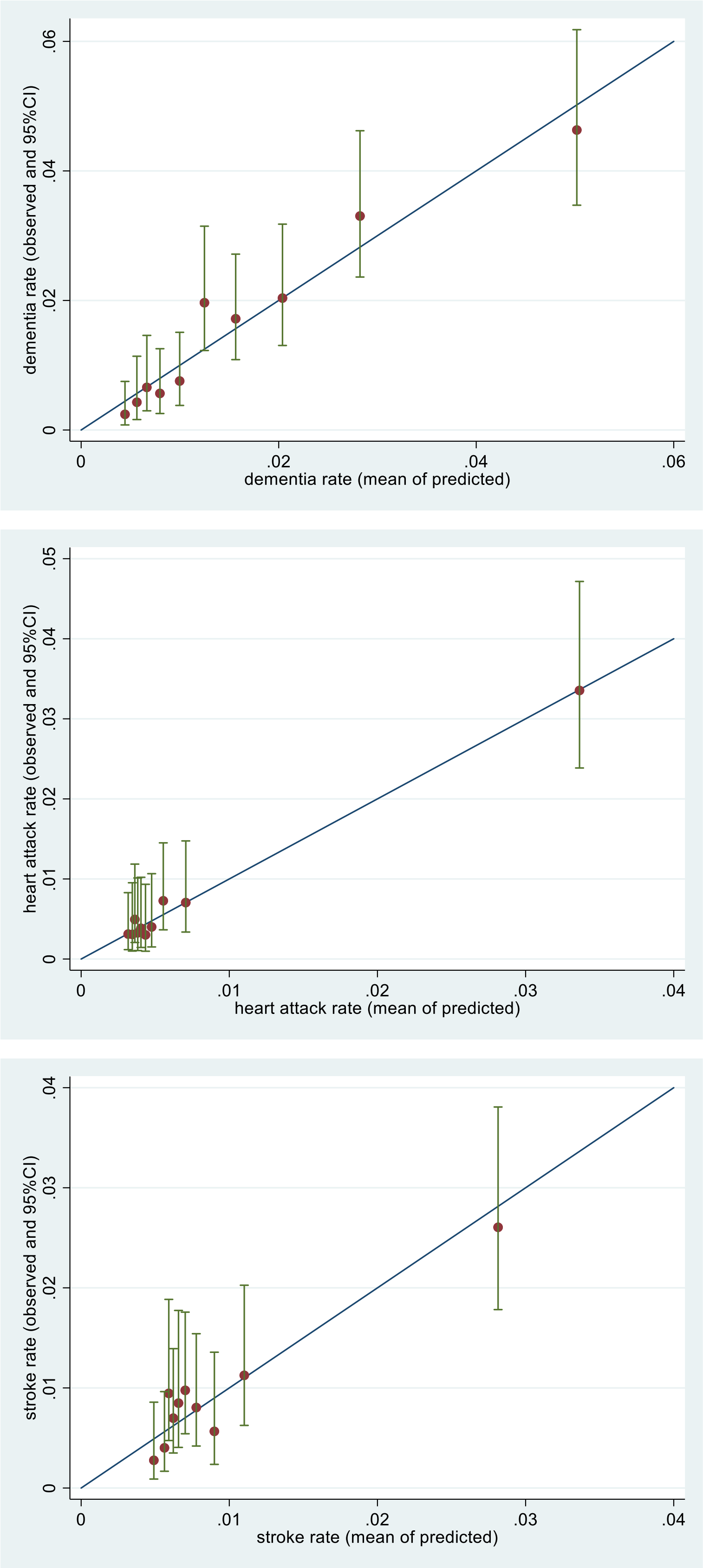

FIGURE S3-2: VALIDATION PLOTS FOR DEMENTIA ONSET EVENT, HEART ATTACK EVENT AND STROKE EVENT (TOP TO BOTTOM). ESTIMATES AT DECILES OF PREDICTED RATE.

We note a limitation of the CFAS-II data in terms of capturing events between baseline and 2-year follow-up for those who died [Brayne, 2006; Brayne, 2017]. For this reasons data after the age of 90 is likely uncertain.

For the health-economic simulation, the incidence rate for those without a history of the other diseases was calculated by applying the exponential function to the linear prediction of the age, sex, age-sex interaction and constant (Equation S3). The incidence rate for those with a history was calculated by multiplying the rate without any disease history to the relative risk related to the disease (i.e., exponential of the GLM coefficient).

Equation S3: Estimate rate from Poisson regression coefficients.

$$r = e^{x_j \beta}$$

r = rate

$x_j\beta$ = linear prediction (sum of each predictor's status and coefficient)

For China the general population incidence rates for dementia onset event, myocardial infarction event and stroke event were obtained from published estimates based on a review of literature. Studies were selected that reflected the whole country and provided age- (and sex-)specific estimates.

For dementia, data were selected from Yuan et al. [2016]. As these data were separate by age category and sex without reported category-specific person-years (see Table S3-3), they were combined as follows to obtain age- and sex-specific estimates. First, person-years were back-calculated from the reported incidence rate, new cases and total persons traced within each age category. Second, the person-years were split by sex by multiplying them by the overall sex prevalence. Third, each age-specific incidence rate was split using Equation S4, using the overall prevalence of each sex. Fourth, the now obtained estimated age- and sex-specific incidence rate was converted to number of events and person-years. Fifth, a Poisson model was fit to these data to obtain coefficients for base rate, age (continuous) and sex, reported in Table S3-4. Although the use of overall sex prevalence for each age category is limited, we believe this is a sufficient approximation of age- and sex-specific estimates.

TABLE S3-3: INCIDENCE RATE OF DEMENTIA PER 1000 PERSON-YEARS (CHINA).

	New cases	Total traced	IR	95%CI lb	95%CI ub
Age					
55-59	15	2417	1.87	1.09	3.01
60-64	28	2999	2.86	1.94	4.07
65-69	33	2937	3.56	2.49	4.93
70-74	63	2185	9.63	7.47	12.24
75-79	75	1364	19.69	15.60	24.54
80-84	50	656	29.19	21.91	38.15
85+	47	332	64.30	47.82	84.72
Gender					
Male	117	5999	6.34	5.27	7.57
Female	194	6882	9.05	7.84	10.39

CI, confidence interval; IR, incidence rate; lb, lower bound; ub, upper bound.

TABLE S3-4: RESULTS OF THE POISSON REGRESSION MODEL FOR AGE- AND SEX-SPECIFIC INCIDENCE RATE OF DEMENTIA IN CHINA.

```
. poisson events age sex_female, exposure(py)

Iteration 0:   log likelihood = -37.010895
Iteration 1:   log likelihood = -37.003866
Iteration 2:   log likelihood = -37.003866

Poisson regression                              Number of obs     =         14
                                                LR chi2(2)        =     345.26
                                                Prob > chi2       =     0.0000
Log likelihood = -37.003866                     Pseudo R2         =     0.8235

------------------------------------------------------------------------------
      events |      Coef.   Std. Err.      z    P>|z|     [95% Conf. Interval]
-------------+----------------------------------------------------------------
         age |   .1255945   .0068925    18.22   0.000     .1120855    .1391035
  sex_female |   .3335382   .1165535     2.86   0.004     .1050976    .5619788
       _cons |  -13.91064   .5243198   -26.53   0.000    -14.93829   -12.88299
      ln(py) |          1  (exposure)
------------------------------------------------------------------------------
```

For myocardial infarction estimates were obtained from Chang et al. [2021] table 1. The median estimate was transformed to mean using the online r package ‘estmeansd’ [https://play158.shinyapps.io/estmeansd/] and gamma distribution, which provided the best fit. A Poisson model was fitted to the reported data on age- and sex-specific events and people at risk (see Table S3-5) to obtain coefficients for base rate, age (continuous) and sex, reported in Table S3-6.

TABLE S3-5: INCIDENCE RATE OF MYOCARDIAL INFARCTION PER 100,000 PERSON-YEARS (MEDIAN CONVERTED TO MEAN) IN CHINA.

Age	Median per 100,000						Male		Female	
	male	IQR lb	IQR ub	female	IQR lb	IQR ub	mean	sd	mean	sd
35-49	148.8	108.6	183.4	12.3	7.2	16	151.8	56.2	12.7	6.7
50-64	372.6	297.6	447.6	83.1	57.3	105.8	380.3	112.5	85.7	36.8
65-79	645.2	506	831.5	445.9	315.8	574.5	680.9	244.5	464.2	195.6
80+	1301	970	1718	1192	881.4	1687	1331.1	555.3	1256.6	598.1

TABLE S3-6: RESULTS OF THE POISSON REGRESSION MODEL FOR AGE- AND SEX-SPECIFIC INCIDENCE RATE OF MYOCARDIAL INFARCTION IN CHINA.

```
. poisson events age sex_female, exposure(py)
note: you are responsible for interpretation of noncount dep. variable

Iteration 0:   log likelihood = -162.40842
Iteration 1:   log likelihood = -161.64392
Iteration 2:   log likelihood = -161.64362
Iteration 3:   log likelihood = -161.64362

Poisson regression                              Number of obs     =          8
                                                LR chi2(2)        =    3286.42
                                                Prob > chi2       =     0.0000
Log likelihood = -161.64362                     Pseudo R2         =     0.9104

------------------------------------------------------------------------------
      events |      Coef.   Std. Err.      z    P>|z|     [95% Conf. Interval]
-------------+----------------------------------------------------------------
         age |   .0637865    .001333    47.85   0.000      .061174    .0663991
  sex_female |  -.3353801   .0307044   -10.92   0.000    -.3955596   -.2752006
       _cons |  -9.618015   .1047718   -91.80   0.000    -9.823364   -9.412666
      ln(py) |          1  (exposure)
------------------------------------------------------------------------------
```

For stroke estimates were selected from Wang et al. [2017]. A Poisson model was fitted to the reported data on age- and sex-specific events and people at risk (see Table S3-7) to obtain coefficients for base rate, age (continuous) and sex, reported in Table S3-8.

TABLE S3-7: INCIDENCE RATE OF STROKE PER 100,000 PERSON-YEARS IN CHINA.

Age	Men			Women		
	No. of strokes	People at risk	IR	No. of strokes	People at risk	IR
20-29	2	2417	4.4	1	47553	2.1
30-39	12	2999	26.4	8	44462	18
40-49	78	2937	139.1	51	55022	92.7
50-59	207	2185	528.5	136	40017	339.9
60-69	264	1364	908.6	224	30352	738
70-79	234	656	1486.9	202	16561	1219.7
80+	106	332	2216.6	118	5906	1998

CI, confidence interval; IR, incidence rate.

TABLE S3-8: RESULTS OF THE POISSON REGRESSION MODEL FOR AGE- AND SEX-SPECIFIC INCIDENCE RATE OF STROKE IN CHINA.

```
. poisson events age sex_female, exposure(py)

Iteration 0:   log likelihood = -130.18361
Iteration 1:   log likelihood = -129.63263
Iteration 2:   log likelihood = -129.63245
Iteration 3:   log likelihood = -129.63245

Poisson regression                              Number of obs     =         14
                                                LR chi2(2)        =    2435.97
                                                Prob > chi2       =     0.0000
Log likelihood = -129.63245                     Pseudo R2         =     0.9038
```

events	Coef.	Std. Err.	z	P>|z|	[95% Conf.	Interval]
age	.075669	.0016721	45.25	0.000	.0723917	.0789463
sex_female	-.2648551	.0496072	-5.34	0.000	-.3620835	-.1676267
_cons	-9.824275	.1150976	-85.36	0.000	-10.04986	-9.598688
ln(py)	1	(exposure)				

For reasons of simplification the interaction between dementia and stroke was not modelled.

3.2 Mortality rate

3.2.1 Life table

For the UK, the general population mortality rates were reflected by estimates published by the Office for National Statistics (ONS) [www.ons.gov.uk]. It represents the United Kingdom population on past and projected mortality rates (qx) created in 2018. The period mortality rate outcome was used to construct a period life table for the year 2018. See Table S3-9 for a copy of this data.

TABLE S3-9: AGE- AND SEX-SPECIFIC MORTALITY RATE PER 100,000 PERSONS PER YEAR IN THE UK (COPIED FROM OFFICE FOR NATIONAL STATISTICS).

Age	Female	Male
50	212.68	339.18
51	234.12	361.32
52	253.10	396.54
53	276.43	431.61
54	285.96	449.72
55	323.77	486.23
56	360.66	529.29
57	395.38	602.90
58	440.93	659.03
59	473.63	689.41
60	516.33	756.14
61	556.64	849.06
62	625.93	920.24
63	679.72	1040.98
64	729.33	1070.45
65	828.01	1217.68
66	866.48	1339.35
67	937.25	1434.12
68	1054.58	1602.20
69	1115.45	1769.53
70	1283.97	1881.89
71	1372.31	2063.60
72	1534.47	2197.84
73	1713.58	2588.58
74	1970.49	2832.24
75	2190.17	3170.03
76	2409.32	3501.79
77	2794.76	3973.86
78	3123.40	4371.05
79	3485.61	4834.32
80	3869.28	5429.43
81	4473.24	6112.46
82	4908.56	6680.44
83	5718.73	7578.43
84	6367.80	8497.54
85	7308.68	9459.85
86	8376.70	10911.50
87	9582.75	11990.89
88	10890.24	13527.55
89	12093.10	15294.33
90	13865.82	16420.76
91	15493.10	17970.68
92	17089.66	20006.29

93	18913.19	21556.93
94	20861.70	23745.70
95	22922.63	25439.60
96	24880.45	28362.40
97	27783.49	30278.86
98	28714.49	31909.41
99	28625.43	33204.13
100	33828.50	36706.92

The proportion of the mortality related to CVD was obtained from the Office for National Statistics (ONS) NOMIS [www.ons.gov.uk]. It represents the proportion underlying cause by age and sex, compiled from information supplied when deaths are certified and registered as part of civil registration, a legal requirement. See Table S3-10 for a copy of this data. Selection of cerebrovascular diseases reflected stroke (i.e., not TIA), and contained I60 Subarachnoid haemorrhage, I61 Intracerebral haemorrhage, I62 Other nontraumatic intracranial haemorrhage and I63 Cerebral infarction. For simplification, the total proportion CVD was used in the model rather than the specific MI and stroke estimates.

TABLE S3-10: PERCENTAGE OF ALL DEATHS BY UNDERLYING CAUSE WITHIN AGE AND SEX BAND IN THE UK.

Age band	Acute myocardial infarction (I21)		Selection of cerebrovascular diseases (I60, I61, I62, I63)		Total	
	Male	Female	Male	Female	Male	Female
50-54	7.0	2.1	2.6	3.1	9.6	5.2
55-59	7.3	2.8	2.4	2.6	9.7	5.4
60-64	6.5	3.1	2.1	2.6	8.6	5.7
65-69	5.7	3.2	1.9	2.8	7.6	6
70-74	5.4	3.1	1.9	2.8	7.3	5.9
75-79	4.6	3.4	2.1	2.7	6.7	6.1
80-84	4.4	3.2	2.2	2.7	6.6	5.9
85-89	3.8	2.8	2.1	2.4	5.9	5.2
90+	2.9	2.1	1.7	1.7	4.6	3.8

For China, the general population mortality rates represented the age- and sex-specific mortality rate from 01-11-2018 to 31-10-2019. See Table S3-11 for a copy of these data. 90+ estimate was used for age 90-100. [China Population and Employment Statistics Yearbook Committee, 2020] For the proportion CVD/non-CVD the UK estimate was used due to lack of identifying estimates.

TABLE S3-11: AGE- AND SEX-SPECIFIC MORTALITY RATE PER 1,000 PERSONS PER YEAR IN CHINA.

Age	Male	Female
50	4.97	1.93
51	3.52	1.31
52	4.67	2.78
53	4.01	1.69
54	6.53	1.37
55	6.72	2.19
56	6.09	3.15
57	4.06	1.79
58	6.63	3.08
59	9.36	3.40
60	10.78	3.46
61	12.36	4.80
62	11.27	5.61
63	12.79	7.86
64	11.74	7.40
65	15.53	6.84
66	15.42	9.93
67	16.72	7.15
68	16.92	12.28
69	24.42	12.60
70	23.25	15.71
71	26.10	12.98
72	33.97	18.60
73	30.92	20.19
74	30.19	26.27
75	40.41	18.48
76	45.13	27.51
77	48.61	32.88
78	45.97	27.63
79	44.78	34.35
80	89.44	51.37
81	70.02	48.89
82	95.38	68.80
83	93.19	65.11
84	111.42	79.37
85	122.29	86.95
86	114.11	68.26
87	149.45	94.72
88	184.03	124.37
89	152.35	126.23
90+	236.42	173.43

3.2.2 Relative risk of death by history of dementia, MI and stroke

For the UK, the relative risk of dementia onset event, MI event and stroke event, by dementia history, MI history and stroke history were obtained from the 3 fitted regression functions in the previous step, by including their baseline history status as predictors.

The relative risk of death by history of dementia, heart attack and stroke were estimated using data from the CFAS-II on death status of the cohort participants after 2-year follow-up. Date and cause of death for CFAS-II use the national death registration system.

Data availability, selection and imputation: identical to earlier for analysis on general population incidence rates for dementia onset event, MI event and stroke event.

- Lost to follow-up: see earlier.
- Imputation: see earlier.
- Selection of age group: see earlier.

A GLM was fit to death status at 2-year follow-up.

The following subsample was used:

- Age 65-89 at baseline

The following predictors were included in the model (forced entry):

- Baseline history of dementia
- Baseline history of heart attack
- Baseline history of stroke

The following covariates were included in the model to obtain adjusted estimates:

- Baseline age (continuous, centred at age of 75)
- Baseline sex

The following model was used:

- Family Poisson
- Link log
- Exposure was assumed 2 years (full follow-up period) for those who did not experience the event and 1 year for those who did experience the event (i.e., assumed half-way during the follow-up period)

The following weights were applied:

- Weights to adjust for sampling to represent the general population (not for attrition as death status was obtained from registry)
- For details, see Matthews et al. [2013]

See Table S3-12 for the results.

TABLE S3-12: RESULTS OF THE WEIGHTED REGRESSION ANALYSIS FOR MORTALITY IN TERMS OF DEATH EVENT DURING 2-YEAR FOLLOW-UP IN CFAS-II DATA.

```
. glm dead ///
>         ib0.dementia_w1         ///
>         ib0.mi_w1
> ///
>         ib0.stroke_w1
>                                         ///
>         c.age_w1_cen i.sex_f ///
>         if age_w1<=89 [pweight=static_weight_v3], fam(poisson) link(log) exposure(exposure_dth)
> nolog vce(robust) eform

Generalized linear models                         No. of obs      =      5,697
Optimization     : ML                             Residual df     =      5,691
                                                  Scale parameter =          1
Deviance         =  5157.283918                   (1/df) Deviance =   .9062175
Pearson          =  17538.69993                   (1/df) Pearson  =   3.081831

Variance function: V(u) = u                       [Poisson]
Link function    : g(u) = ln(u)                   [Log]

                                                  AIC             =   1.279794
Log pseudolikelihood = -3639.492562               BIC             =  -44056.75

------------------------------------------------------------------------------
             |               Robust
        dead |        IRR   Std. Err.      z    P>|z|     [95% Conf. Interval]
-------------+----------------------------------------------------------------
1.dementia_w1 |   5.823019   .6647491    15.43   0.000     4.655604    7.283169
      1.mi_w1 |   1.429407   .1810695     2.82   0.005     1.115142    1.832236
  1.stroke_w1 |   1.272421   .1850341     1.66   0.098     .9568635    1.692044
   age_w1_cen |   1.098866   .0081612    12.69   0.000     1.082986    1.114978
      1.sex_f |   .8731088   .0775183    -1.53   0.126     .7336605    1.039062
        _cons |   .0355501   .0026308   -45.09   0.000     .0307503     .041099
ln(exposur~h) |          1  (exposure)
------------------------------------------------------------------------------
Note: _cons estimates baseline incidence rate.
```

age_w1_cen = age at baseline centred at 75; sex_f = female sex; dementia_w1 = history of dementia onset at baseline; mi_w1 = history of MI at baseline; stroke_w1 = history of stroke at baseline. # represents interaction term.

The face validity of the model was judged as follows. The observed unweighted rate (and confidence interval) was compared to the mean unweighted predicted rate, by deciles of the predicted rate. It was considered valid based on visual inspection of the plot, see Figure S3-3.

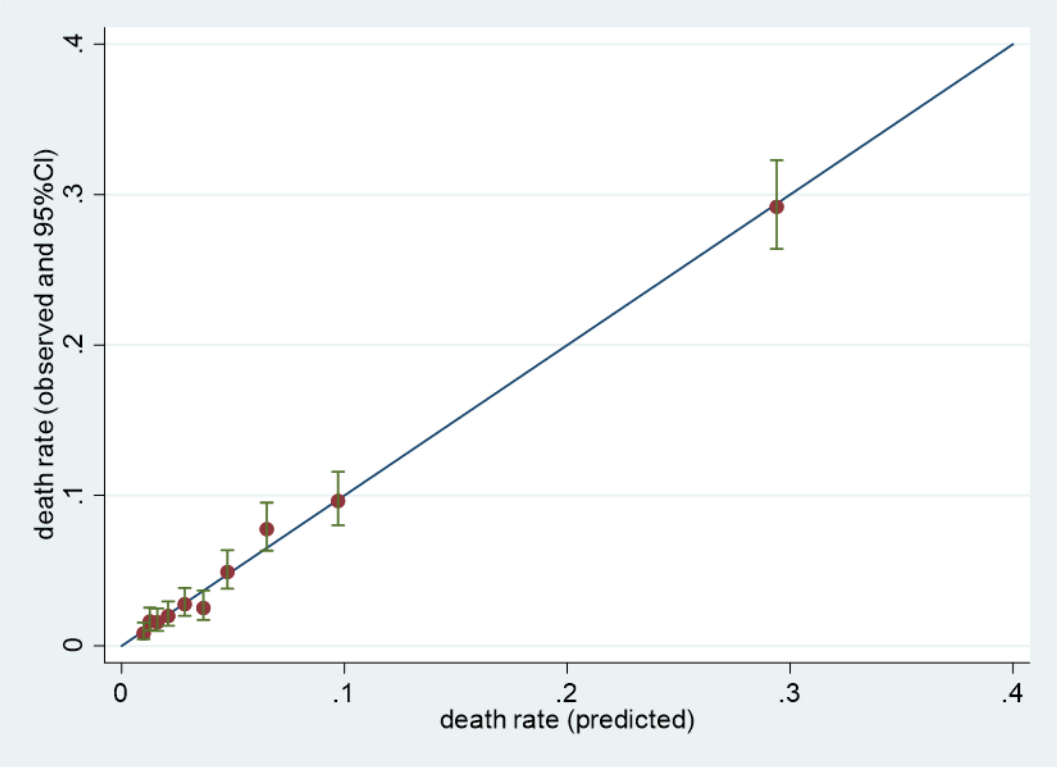

FIGURE S3-3: VALIDATION PLOT FOR DEATH EVENT. ESTIMATES AT DECILES OF PREDICTED RATE.

For the health-economic model the age- and sex-specific rate in the at-risk state was reflected by the life table rate. The age- and sex-specific rate in the disease states (dementia, MI and stroke) was calculated by multiplying the life table rate with the IRR from the GLM equation for the specific disease. For states reflecting multiple disease the corresponding IRRs were multiplied.

For China, the relative risk of death by dementia was 3.02 (95%CI: 2.13-4.28) in urban and 3.59 (95%CI: 2.47-5.21) in rural area [Prince, 2012]. This was averaged by taking the mean of both estimates. The relative risk of stroke was calculated by dividing the stroke mortality rate [Wang, 2017] by the life table rate for each age band and sex, and taking the mean of these estimates, being a rate ratio of 4.3. The relative risk for myocardial infarction was assumed the same as for stroke.

3.2.3 Split mortality rate to prevent 'double counting'

The model-predicted control strategy age- and sex-specific mortality prevalence was compared to the observed age- and sex-specific mortality prevalence from a general population life table. An unadjusted starting population was used (i.e., reflecting the general population using the base case estimates from CFASII, not adjusted to reflect the PRODEMOS target population at increased risk), with a fixed starting age of 65. The life table mortality prevalence was obtained from the UK Office for National Statistics (ONS) 2017-2019 [www.ons.gov.uk] representing the period mortality.

Figure S3-4 shows the model-predicted prevalence alive and the life table proportion alive without adjustment. Visual assessment of the graphs shows a moderate underestimation of the prevalence alive in both sexes (i.e., overestimation of mortality).

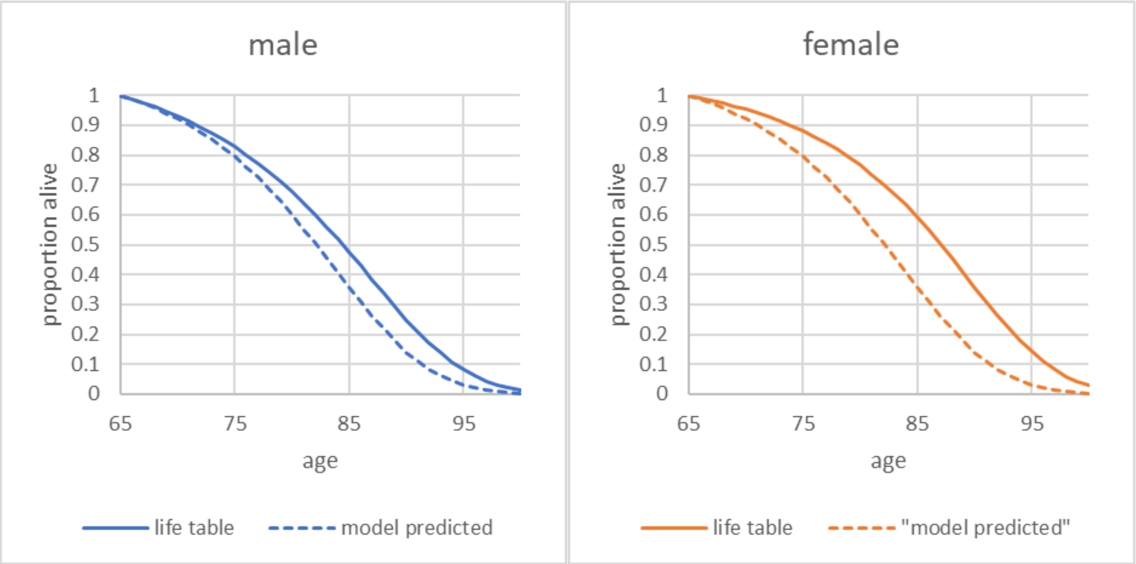

FIGURE S3-4: MODEL-PREDICTED MORTALITY PREVALENCE ALIVE AND THE LIFE TABLE PROPORTION ALIVE BEFORE ADJUSTMENT.

We believe this is because the general population life table contains mortality related to dementia, MI and stroke. As the model multiplies the general population mortality rate with the RR related to dementia, MI and stroke, mortality is overestimated (i.e., it is 'double counted'). Therefore, the life table rate was split to adjusted for this overestimation.

A rate can be split to obtain the rate specific for those unexposed (i.e., without dementia history). This is because a rate can be seen as a weighted average of subgroup rates with a risk factor's prevalence providing the subgroup weights (see Equation S4-(1)), similar to weighted probabilities described by Gidwani and Russell in paragraph 4.2 [Gidwani, 2020].

This equation was rewritten to obtain the relative risk of unexposed versus the average exposure level (i.e., dementia prevalence) (see Equation S4-(6)). This particular relative risk is applied as the adjustment factor for the mortality rate to obtain the mortality rate in those unexposed to the external risk factor (i.e., without dementia history).

Now, the adjusted base rate can be multiplied with the relative risk of an external factor (e.g., exposed versus unexposed to dementia history) as the reference category of the external factor's relative risk (being unexposed to dementia history) now corresponds to the base rate (being adjusted to reflect specifically those unexposed to dementia history).

This was done separately for each disease (dementia, MI and stroke), and separately for mortality due to CVD and mortality due to other.

EQUATION S4: ADJUSTMENT FACTOR (EXPRESSED AS RELATIVE RISK) BASED ON INCIDENCE RATE, RELATIVE RISK AND PREVALENCE.

$$ir_{gp} = ir_{a0}p_{a0} + ir_{a1}p_{a1} \ (1)$$

$$ir_{a1} = ir_{a0}RR_{a1\_0} \ (2)$$

Fill in (2) in (1):

$$ir_{gp} = ir_{a0}p_{a0} + ir_{a0}RR_{a1\_0}p_{a1} \ (3)$$

Rewrite:

$$ir_{gp} = ir_{a0}\left(p_{a0} + RR_{a1\_0}p_{a1}\right) \ (4)$$

$$ir_{a0} = \frac{ir_{gp}}{p_{a0}+RR_{a1\_0}p_{a1}} \ (5)$$

Rewrite to obtain adjustment factor for risk factor a with 2 categories (a0, a1) (expressed as relative risk ir_{a0} relative to ir_{gp}):

$$RR_{a0\_gp} = \frac{ir_{a0}}{ir_{gp}} = \frac{1}{p_{a0}+RR_{a1\_0}p_{a1}} \ (6)$$

Age- and sex-specific prevalence of dementia, MI and stroke were obtained from CFASII data. Age (65-100) was categorized in bands of 5 years. Mean observed prevalence in each age band was used. For RR of death related to each disease, the base case estimate was used.

Figure S3-5 shows the model-predicted mortality prevalence alive and the life table proportion alive after adjustment. We judge the deviation in prevalence alive as small for male and moderate for female. We judge this deviation is acceptable for our model purpose.

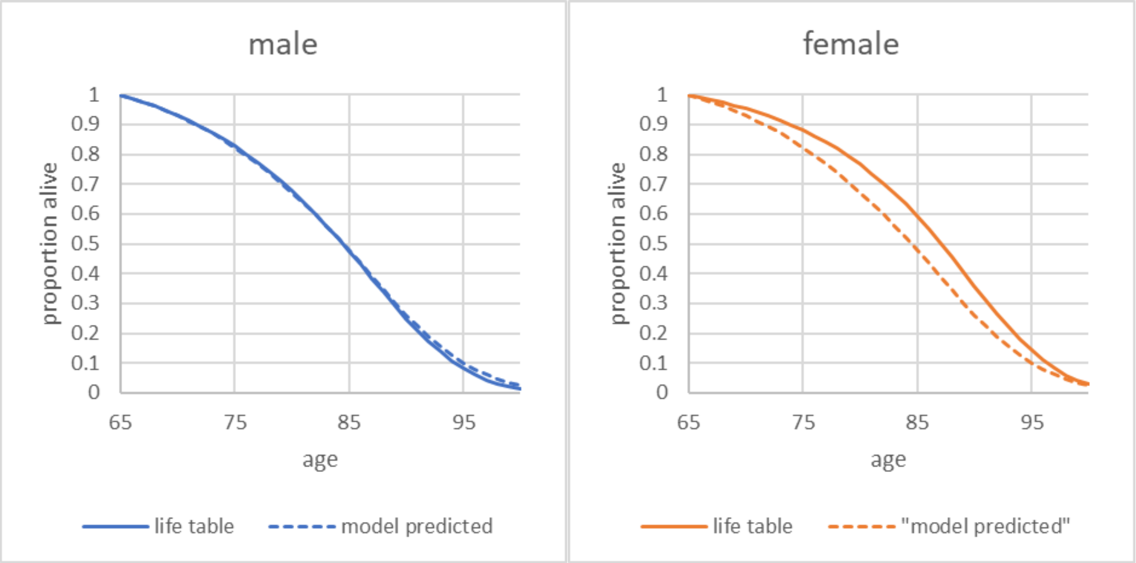

FIGURE S3-5: MODEL-PREDICTED PREVALENCE ALIVE AND THE LIFE TABLE PROPORTION ALIVE, BOTH AFTER ADJUSTING THE MORTALITY RATE TO PREVENT DOUBLE COUNTING THE MORTALITY RELATED TO DEMENTIA, MYOCARDIAL INFARCTION AND STROKE.

The same method was applied to adjust the Chinese mortality rate for dementia, MI and stroke, using dementia prevalence [Zhang, 2005: table 3 both Alzheimer and vascular], stroke prevalence [Wang, 2017: table 2] and assuming the prevalence for MI is the same as stroke for reasons of not having identified MI prevalence estimates by age and sex.

3.3 Starting population

3.3.1 Starting age, sex and disease distribution

The starting population was reflected by the PRODEMOS total baseline sample distribution of age band, sex, history of MI and history of stroke. Age band was reflected by the mid-point age of the band. The model was run for each combination to allow reflecting heterogeneity in the starting population. Model outcomes were weighted by the prevalence (i.e., proportion of total sample). We believe reflecting this heterogeneity is important as we expect the health-economic outcomes at the sample mean to be different from the mean of the health-economic outcomes of each subsample. For example, life expectancy is longer in younger age, creating a larger window for events in life years saved.

See Table S3-13 for the sample distribution in UK and Table S3-14 for China.

TABLE S3-13: DISTRIBUTION OF PRODEMOS BASELINE SAMPLE CHARACTERISTICS IN UK.

Age band mid-point	sex	Proportion of total sample	Proportion with history of MI	Proportion with history of stroke
57	0	0.09	0	0.06
57	1	0.14	0	0.02
62	0	0.09	0.13	0.06
62	1	0.13	0.05	0.01
67	0	0.12	0.10	0.08
67	1	0.14	0.03	0.05
72	0	0.11	0.13	0.04
72	1	0.17	0.08	0.09

TABLE S3-14: DISTRIBUTION OF PRODEMOS BASELINE SAMPLE CHARACTERISTICS IN CHINA.

Age band mid-point	sex	Proportion of total sample	Proportion with history of MI	Proportion with history of stroke
57	0	0.15	0.01	0.09
57	1	0.24	0.01	0.04
62	0	0.10	0.10	0.12
62	1	0.13	0.03	0.14
67	0	0.09	0.11	0.22
67	1	0.15	0.04	0.12
72	0	0.06	0.15	0.13
72	1	0.08	0.04	0.14

3.3.2 Disease incidence

An unadjusted disease incidence rate corresponds to the average risk factor status of the population it is estimated in (i.e., the prevalence of exposes/unexposed). The incidence rate in another target population with a different prevalence of exposure can be calculated in combination with the relative risk related to the risk factor. This can be done by raising the relative risk to the power of the difference between exposure level between the target and the reference population (given all reflect the same unit).

For example, for the UK model the incidence rate was adjusted for age, sex and history of disease (see GLM model described in supplementary material 3.1) but not for risk factor obesity/BMI, and thus reflects the incidence at the average obesity/BMI status of the population the rate was measured in (i.e., the reference population: the general population as aimed by the CFASII cohort study). The PRODEMOS trial UK subsample showed higher prevalence of obesity (0.57) and mean of 31.8, as compared to 35% and 28.2 in the reference population, see Table S3-15. The dementia incidence rate in the PRODEMOS target population can be calculated using the relative risk of 2.17 for obesity as 2.17^(0.57-0.35)=1.19. The MI incidence rate for men in the PRODEMOS target population can be calculated using the relative risk of 1.022 per 1 BMI point as 1.022^(31.8-28.2)=1.08. The dementia risk in the PRODEMOS target population was thus 1.19 times higher as in the reference general population, and 1.08 for MI. Table S3-15 provides an overview of the RRs and corresponding prevalence or mean in the reference general as well as in the PRODEMOS target population (for sources see supplementary material 2 and PRODEMOS trial outcomes [Moll van Charante, 2024: appendix 11]), and the relative risk to convert the reference to the target population, which was the product of all individual relative risks. For dementia the conversion RR was 4.285, for MI and stroke in males 1.156 and for MI and stroke in females 1.188.

Table S3-15: RR to convert reference to target population in UK.

Factor	RR	Prevalence (p) or mean (mn) general population	Prevalence or mean PRODEMOS baseline sample	RR to convert reference to target population (adjustment factor)
Dementia				
Education mid (p)	2.33	0.28	0.495	1.20
Education low (p)	3.24	0.01	0.130	1.15
Deprivation mid (p)	1.4	0.33	0.420	1.03
Deprivation low (p)	1.5	0.33	0.558	1.10
Depression (p)	1.1	0.067	0.465	1.04
Hypertension (p)	2.1	0.39	0.81	1.37
Obesity (p)	2.17	0.35	0.57	1.19
Hypercholesterolaemia (p)	1.81	0.34	0.796	1.31
Physical inactivity (p)	1.64	0.25	0.7075	1.25
Smoking past (p)	1.1	0.51	0.502	1.00
Smoking current (p)	1.7	0.11	0.09	0.99
<u>Product</u>				<u>4.285</u>
CVD male				
deprivation (index)	1.017	0	2.2	2.2
Diabetes (p)	1.6	0.14	0.20	1.029
Systolic blood pressure (mmHg)	1.004	135	137	1.008
BMI (kg/m2)	1.022	28.2	31.8	1.081
Current smoking (p)	1.417	0.11	0.09	0.993
<u>Product</u>				<u>1.156</u>
CVD female				
deprivation (index)	1.035	0	2.2	2.2
Diabetes (p)	2	0.14	0.20	1.042
Systolic blood pressure (mmHg)	1.005	135	137	1.010
BMI (kg/m2)	1.015	28.2	31.8	1.055
Current smoking (p)	1.530	0.11	0.09	0.992
<u>Product</u>				<u>1.188</u>

Abbreviations: CVD, cardiovascular disease (reflecting myocardial infarction and stroke); p, proportion; RR, relative risk.

For China, the prevalence or mean in the general population differed, see supplementary material 2. In addition, the prevalence or mean in the PRODEMOS baseline sample was country specific. See Table S3-16 for details.

TABLE S3-16: RR TO CONVERT REFERENCE TO TARGET POPULATION IN CHINA.

Factor	RR (same as UK)	Prevalence or mean general population	Prevalence or mean PRODEMOS baseline sample	RR to convert reference to target population (adjustment factor)
Dementia				
Education mid (p)	2.33	0.543	0.267	0.79
Education low (p)	3.24	0.353	0.333	0.98
Deprivation mid (p)	1.4	n/a	n/a	n/a
Deprivation low (p)	1.5	n/a	n/a	n/a
Depression (p)	1.1	0.038	0.055	1.00
Hypertension (p)	2.1	0.275	0.579	1.25
Obesity (p)	2.17	0.141	0.232	1.07
Hypercholesterolaemia (p)	1.81	0.083	0.796	1.53
Physical inactivity (p)	1.64	0.219	0.7075	1.27
Smoking past (p)	1.1	n/a	n/a	n/a
Smoking current (p)	1.7	0.496	0.14	0.83
Product				1.677
CVD male				
deprivation (index)	n/a	n/a	n/a	n/a
Diabetes (p)	1.6	0.124	0.284	1.078
Systolic blood pressure (mmHg)	1.004	127.7	128	1.001
BMI (kg/m^2)	1.022	24.1	25.9	1.109
Current smoking (p)	1.417	0.496	0.14	0.883
Product				1.057
CVD female				
deprivation (index)	n/a	n/a	n/a	n/a
Diabetes (p)	2	0.124	0.284	1.078
Systolic blood pressure (mmHg)	1.005	127.7	128	1.001
BMI (kg/m^2)	1.015	24.1	25.9	1.109
Current smoking (p)	1.530	0.496	0.14	0.860
Product				1.032

Abbreviations: CVD, cardiovascular disease (reflecting myocardial infarction and stroke); p, proportion; RR, relative risk.

3.4 Implementation of intervention effect

The PRODEMOS trial showed a difference in mean change from baseline (95% confidence interval) to 12-18 months follow-up between the control and intervention arm [Moll van Charante, 2024]

- for systolic blood pressure of -0.8 (-2.1 to 0.5) mmHg and hypertension (systolic blood pressure >140mmHg) of -1.1% (-5.2 to 3.0),
- for BMI of -0.21 (-0.46 to 0.05) kg/m^2 and obesity (UK: BMI >30 kg/m^2, CN: >28 kg/m^2) of -2.3% (-5.4 to 0.7),
- for hypercholesterolemia (total cholesterol >6.5 mmol/L) of -1.0% (-3.4 to 1.3),
- for physical inactivity (<2.5hour/week moderate-high intense activity) of -6.7% (-11.2 to -2.1), and
- for smoking of -3.1% (-4.8 to -1.5).

These estimates are based on the complete case according to intention-to-treat sample (82.7%). This was implemented into the model as a relative risk compared to the natural incidence rates for developing each of the 3 diseases. The same method as for reflecting the

PRODEMOS target population was used (see supplementary material 3.3.2). The same RRs were raised to the difference in risk factors between the target population (i.e., intervention strategy with reduced risk factor status) and its reference population (i.e., control strategy without reduced risk factor status). Table S3-17 provides an overview of the RRs and corresponding prevalence or mean in the reference control as well as in the target intervention population (for sources on RRs see supplementary material 2), and the relative risk to convert the reference to the target population, which was the product of all individual relative risks. This was considered valid as the RRs were obtained from the CAIDE risk score for dementia and QRISK score for myocardial infarction and stroke, both based on a multivariable analysis adjusting each factor for one another. For dementia the treatment effect RR was 0.924, for MI and stroke in males 0.982 and for MI and stroke in females 0.980. A 95% bootstrap interval was calculated by taking the 2.5th and 97.5th percentile of the relative risk for dementia, MI and stroke based on 10,000 random (bootstrap) draws from the separate risk factors uncertainty distribution (i.e., 95% confidence interval). See supplementary material 4 for extrapolation of the effect related to adherence.

The same treatment effect was applied both to UK and China to align with the PRODEMOS trial effectiveness analysis, as no significant difference in effect according to country was observed [Moll van Charante, 2024].

TABLE S3-17: RR TO CONVERT REFERENCE TO TARGET POPULATION (BOTH UK AND CHINA).

Factor	RR	Difference in mean change from baseline	RR to convert reference to target population (adjustment factor)
Dementia (CAIDE)			
Hypertension (p)	2.1	-0.011	0.992
Obesity (p)	2.17	-0.023	0.982
Hypercholesterolaemia (p)	1.81	-0.01	0.994
Physical inactivity (p)	1.64	-0.067	0.967
Smoking past (p)	1.1	0.031	1.003
Smoking current (p)	1.7	-0.031	0.984
Product (95% bootstrap interval)			0.924 (0.768 - 1.108)
CVD male (QRISK)			
Systolic blood pressure (mmHg)	1.004	-0.8	0.997
BMI (kg/m2)	1.022	-0.21	0.995
Current smoking (p)	1.417	-0.031	0.989
Product (95% bootstrap interval)			0.982 (0.946 - 1.018)
CVD female (QRISK)			
Systolic blood pressure (mmHg)	1.005	-0.8	0.996
BMI (kg/m2)	1.015	-0.21	0.997
Current smoking (p)	1.53	-0.031	0.987
Product (95% bootstrap interval)			0.980 (0.942 - 1.018)

3.5 Transform between rate and transition probability.

All rates were converted to transition probabilities using Equation S5 based on principles and equations described elsewhere [Fleurence, 2007; Gidwani, 2020]. Mortality was applied first, after which transitions between diseases took place.

EQUATION S5: RATE TO PROBABILITY AND VICE VERSA.

$$p = 1 - e^{-r} \ (1)$$

$$r = -\ln(1-p)\mathrm{RR}\frac{t_{new}}{t_{old}} \ (2)$$

p = probability

r = rate

RR = relative risk

t_{new} *= targeted time period for the rate (e.g., 0.25 year, representing 3 months)*

t_{old} *= original time period of the probability (e.g., 2 years, representing the CFAS-II time interval)*

3.6 Utilities and costs

3.6.1 Utilities UK

We reviewed NICE technology appraisals on cardiovascular risk management for utility estimates relevant to our target population and setting, specific for UK. Utility estimates for dementia were selected from a systematic review.

As recommended by Ara et al. [2010] we used general population age- and sex-specific utilities as baseline. They were obtained by Ara et al. [2010], which was an update of the source used in previous technology assessments for CVD prevention (drug) interventions [Ara, 2008; Campbell, 2015; Ward, 2007] based on the data of the Health Survey for England 2003 and 2006. See Equation S6 to estimate the age- and sex-specific values [Ara, 2010]. We assumed this was representative for the PRODEMOS target population.

EQUATION S6: UTILITY IN GENERAL POPULATION

$$U_{gp} = 0.9508566 + 0.0212126\mathrm{male} - 0.0002587\mathrm{age} - 0.0000332\mathrm{age}^2$$

U = utility

gp = general population

Furthermore, as supported by Ara et al. [2010] we applied a multiplicative approach to combine utilities from multiple history of conditions (i.e., multiplying the ratio between condition-specific utility and baseline utility (corresponding to the mean age and sex of the condition-specific utility), for each condition). We applied a disutility for MI and stroke disease events.

The utility corresponding to dementia was obtained by selecting studies from a systematic literature review [Landeiro, 2020] representing a UK non-hospital setting covering all dementia stages with relatively large sample size, which was 1 study [Wimo, 2013]. This cohort study recruited community-dwelling persons from specialist secondary care clinics (i.e., memory clinics). Estimates were 0.68 (95%CI: 0.65-0.72) for mild, 0.65 (95%CI: 0.61-0.69) for moderate and 0.48 (95%CI: 0.43-0.53) for moderately severe/severe at mean age of 78.5. These estimates were weighted for the proportion living in each severity state [Prince, 2014], being 0.554 in mild, 0.321 in moderate and 0.125 in severe. This resulted in a mean weighted utility of 0.65 (0.61 and 0.69 when using severity-specific lower and upper bound utility values respectively). The ratio compared to the general population utility at the corresponding age (78.5) of 0.737 was 0.88.

The utility corresponding to a heart attack in the past 12 months without history of other CVD conditions (mean=0.721, SE=0.045, at mean age 65.4) and with history of just heart attack with no event taken place in the past 12 months (mean=0.742, SE=0.020, at mean age of 65.1) were obtained from Ara et al. [2010] based on the data of the Health Survey for England 2003 and 2006. The ratio compared to the general population utility at the corresponding age (65.1) of 0.804 was 0.92. Disutility for MI event was calculated as the utility of the event (same method as above) minus the utility of a history of the disease (being -0.025).

The utility corresponding to a stroke in the past 12 months without history of other CVD conditions (mean=0.626, SE=0.038, at mean age of 67.9) and history of just stroke with no event taken place in the past 12 months (mean=0.668, SE=0.018, at mean age of 66.8) were obtained from the same source as for heart attack. The ratio compared to the general population utility at the corresponding age (66.8) of 0.796 was 0.84. Disutility for stroke event was calculated as the utility of the event (same method as above) minus the utility of a history of the disease (-0.048).

The utility of CVD event in the past 12 months or without event with a history of the disease was modelled in different ways. The utility related to 'no event with history of disease' was modelled using the Markov state trace (i.e., multiply the proportion in state with the utility related to 'no event with history of disease'). The utility related to 'event in past 12 months' was modelled using the transitions from other states, omitting the transitions to the same state (i.e., multiply the proportion transitions from other states with the utility related to 'event in past 12 months). The utility related to 'no event with history of disease' was subtracted from the utility related to 'event in past 12 months' to prevent double counting.

3.6.2 Utilities China

We performed a scoping review for utility estimates relevant to our target population and setting, specific for China.

We followed the same method as recommended by Ara et al. [2010] to use general population age- and sex-specific estimates as baseline and a multiplicative approach to combine utilities from multiple history of conditions, and a disutility for stroke disease events.

We believed the estimates by Xie et al. [2022] fit our target population and setting and contained estimates for dementia, MI and stroke within the same study (limiting differences due to methodological variation). EQ-5D-5L estimates were obtained from this valuation study conducted in 2019 in a representative sample of the Chinese general population, in terms of age, gender, education, and area of residence (urban/rural). A Chinese value set was developed using a time trade-of approach in a sample of the general population living in an urban setting. We fitted the same regression function as for the UK general population estimates to the mean estimates reported in table 2 from Xie et al. [2022], using the number of observations as frequency weight, which resulted in the estimates reported in Table S3-18.

TABLE S3-18: REGRESSION MODEL FIT TO MEAN UTILITY ESTIMATES REPORTED IN TABLE 2 FROM XIE ET AL. [2022].

```
. regress utility i.sex2 age_mean age_mean2 [fweight=n]
```

Source	SS	df	MS
Model	.57784714	3	.192615713
Residual	.625883861	3,393	.000184463
Total	1.203731	3,396	.000354456

Number of obs	=	3,397
F(3, 3393)	=	1044.20
Prob > F	=	0.0000
R-squared	=	0.4800
Adj R-squared	=	0.4796
Root MSE	=	.01358

utility	Coef.	Std. Err.	t	P>|t|	[95% Conf.	Interval]
sex2						
1. male	.0050488	.0004677	10.80	0.000	.0041319	.0059657
age_mean	.0023842	.0000813	29.31	0.000	.0022247	.0025437
age_mean2	-.0000319	8.58e-07	-37.12	0.000	-.0000335	-.0000302
_cons	.9091741	.0018164	500.54	0.000	.9056128	.9127355

The utility corresponding to dementia (defined as 'memory-related disease') was estimated 0.689 (95%CI: 0.478 to 0.900) in the same study as the general population estimates [Xie, 2022]. For the multiplicative approach we assumed this reflected a mean age of 78.5, similar to the UK estimate for dementia utility. The ratio compared to the general population utility at the corresponding age (78.5) of 0.902 was 0.76.

The utility corresponding to MI history (defined as heart disease) was estimated 0.875 (95%CI: 0.875 to 0.187) in the same study as the general population estimates [Xie, 2022]. The ratio compared to the general population utility at the corresponding age (65.1 assumed same age as UK utility estimate) of 0.932 was 0.94. The disutility corresponding to MI 1 year post event was assumed the same as UK, being -0.025.

The utility corresponding to stroke history was estimated 0.719 (95%CI: 0.560 to 0.879) in the same study as the general population estimates [Xie, 2022]. The ratio compared to the general population utility at the corresponding age (66.8 assumed same age as UK utility estimate) of 0.929 was 0.77. The disutility corresponding to stroke 1 year post event was assumed the same as UK, being -0.048.

The same method as for the UK utility was applied to model utility related to 'event in past 12 months' and 'no event with history of disease'.

3.6.3 Costs UK

We reviewed NICE technology appraisals on cardiovascular risk management for cost estimates relevant to our target population and setting, specific for UK. Cost estimates for dementia were selected from a systematic review.

A cost estimate for dementia was selected from the preliminary results of an unpublished systematic review [Landeiro, 2018]. The estimate from Wubker et al. [2015] was used, obtained from participants recruited for a cohort study in home (and considered at risk of institutionalization) and nursing care organizations with mean age 81-87. We believed this fitted best our target population and setting. The estimates for health sector (formal medical and social care) and informal care were weighted for the proportion living in each severity state [Prince, 2014], being 0.554 in mild, 0.321 in moderate and 0.125 in severe, and the proportion living in a nursing home setting [Prince, 2014], being 131,230 out of 311,730 among the 38.7% who are living in residential care or nursing home (among the total population of dementia living in the community and residential care or nursing home). The attributable fraction was estimated by multiplying it with the mean of a published attributable factor related to community-based (45%) and nursing home-based care (64%) from a systematic literature review [Velandia, 2022]. This resulted in an estimate of €7,508 (4,423 to 10,597 when using lower and upper bound cost values respectively) for health sector and 9,444 (5,954 to 12,933) for informal care costs per year attributed to dementia. In summary, health care sector costs were €7,508 (2010) for post-year 1 and €7,508 (2010) for year 1; informal care costs were €9,444 (2010) in post-year 1 and €9,444 (2010) in year 1.

The costs for MI were obtained from Palmer et al. [2006]. This source was selected as it was used in decision models [Ward, 2007; Ara, 2009] used to generate evidence for NICE clinical guideline on cardiovascular disease risk reduction. The study by Palmer et al. [2006] estimated the MI-related costs based on aggregating the hospital-based resource use data from the 1998 Nottingham Heart Attack Register to an annual average health state cost, including revascularisation for a proportion of patients, which were reported £3,966 (SD=£1,722; reflecting costing year 1999) in the first year. Evidence for NICE clinical guidelines [Ward, 2005; Ara, 2008] assumed also the same costs for non-stable angina in the first year (£477; reflecting year 2006 from Ara et al. [2008]), which was assumed "as stable angina costs plus 60% of patients on clopidogrel". The costs for stable angina (£201; reflecting year 2006 from Ara et al. [2008]) was assumed "3 times 15 minutes GP contact plus medication costs". Evidence for NICE clinical guidelines [Ward, 2005; Ara, 2008] assumed the costs related to post-year 1 MI were the same as stable angina. Because no literature was retrieved on informal care costs after MI in UK we assumed these were absent. In summary, health care sector costs were £201 (2006) for post-year 1 and £4,443 (1999) for year 1; informal care costs were £0 in post-year 1 and £0 in year 1.

A systematic review [Strilciuc, 2021] identified 2 studies on the costs of stroke [Youman, 2003; Saka, 2009]. The study by Youman et al. [2003] was selected as this was also used in decision models [Ward, 2007; Ara, 2009] used to generate evidence for NICE clinical guideline on cardiovascular disease risk reduction. The study by Youman et al. [2003] was based on a randomized trial including 457 acute-stroke patients recruited from a population-based stroke register with observed resource use over 1 year follow-up (with 3-month

interval) in terms of hospital and other health services, social services and informal care resources valued at the UK minimum wage. Incremental costs were estimated by subtracting the costs reported in a period before the stroke for 2001/2002 price index year. The acute inpatient costs (i.e., in the first 3 months after stroke, after which 99% was no longer in hospital) was weighted by stroke severity and calculated GBP £7,965. The 3-month costs of ongoing care after discharge were weighted by stroke severity and discharge location (home/institution) and calculated GBP £698 excluding informal care and GBP £1,877 including informal care. The costs of stroke in the first year was assumed a combination of acute and after discharge and inpatient only, the costs in subsequent year consisted of costs after discharge only. In summary, health care sector costs were £2,792 (2002) for post-year 1 and £10,059 (2002) for year 1; informal care costs were £4,717 (2002) post-year 1 and £4,717 (2002) for year 1.

All costs were converted to Great British Pound (GBP) sterling using exchange rates [www.ons.gov.uk] and to consumer price index year 2021 [www.ons.gov.uk]. Costs were converted to 2021 using consumer price indices [www.ons.gov.uk, https://fred.stlouisfed.org/series/CHNCPIALLMINMEI].

The same method as for the UK utility was applied to model costs related to 'year 1' and 'post-year 1'.

3.6.4 Costs China

We performed a scoping review for cost estimates relevant to our target population and setting, specific for China.

A cost estimate for dementia was obtained from Yan et al. [2019]. Participants aged 60 years or older were invited for a cross-sectional study in 30 provincial, municipal and autonomous regions in eastern, central and western geographic areas in 2015-2016. Costs included direct medical (outpatient, hospitalization, out-of-pocket), direct non-medical (transportation, accommodation, meals when visiting a physician; nourishment and health-care equipment in the patient's daily life; formal care fees at the nursing home, care facility, or at home), indirect costs (work loss, reduction informal caregivers' income), intangible costs (treatment mental suffering of caregivers and unexpected injuries). Among 3046 participants with mean age of 75.27 and MMSE of 13.75 the average costs per patient per year was $19,144 (CNY 122,523). Attributable costs due to dementia were estimated by the costs in the subgroup of those without any comorbidities, which was $2,965 (SD=4,786, n=905) for direct medical and $9,306 (SD=32,955, n=905) for indirect costs, which could be considered the costs of dementia unrelated to/adjusted for comorbidities. 95% confidence interval was estimated using SD, sample size and formulas SE = SD / sqrt(n) and 95%CI = ± 1.96SE. In summary, health care sector costs were $2,965 (2015) for post-year 1 and $2,965 (2015) for year 1; informal care costs were $9,306 (2015) for post-year 1 and $9,306 (2015) for year 1.

A cost estimate for MI was copied from Zhou et al. [2020] who performed a health-economic evaluation on anti-hypertension treatment. For costs of coronary artery disease in the first year they used $4,374.54 (range for sensitivity analysis $1,285.49 to $1,2916.95), referring to the China's health and family planning statistical yearbook. For costs in the subsequent years they used $397.69 (range for sensitivity analysis $130.47 to $1174.26) referring to a health-

economic evaluation [Chen, 2017], who referred to Yuan et al. [2013], article in Chinese language. These costs are assumed health care sector as informal care is not mentioned. In summary, health care sector costs were $398 (2017) for post-year 1 and $4,375 (2017) for year 1; informal care costs were $0 post-year 1 and $0 for year 1.

A cost estimate for stroke was obtained from the same source as MI [Zhou, 2020]. For costs in the first year they used $3,017.58 (range for sensitivity analysis $954.07 to $8,977.11), referring to the China's health and family planning statistical yearbook. For costs in the subsequent years they used $1,416.73 (range for sensitivity analysis $299.24 to $4,250.19) referring to a health-economic evaluation [Chen, 2017], who referred to Yuanmei et al. [2010], article in Chinese language. These costs are assumed care sector as informal care is not mentioned. We did not find an estimate for informal care costs and therefore applied the same proportion as the UK estimate (1.7 ratio between informal and health sector care costs in post-year 1). In summary, health care sector costs were $1,417 (2017) for post-year 1 and $3,018 (2017) for year 1; informal care costs were 1.7*$1,417 (2017) for post-year 1 and 1.7*$1,417 (2017) for year 1.

All costs were converted to Chinese Yuan (CNY) using exchange rates [https://www.exchangerates.org.uk] and to consumer price index year 2021 [fred.stlouisfed.org].

The same method as for the UK utility was applied to model costs related to 'year 1' and 'post-year 1'.

3.6.5 Costs intervention

The costs of the intervention were estimated in terms of the mobile platform costs and coaching personal costs. No out-of-pocket costs was assumed.

The mobile platform costs when implemented in practice was provided by the platform manufacturers from China, who indicated €4.8 per end-user (single end-user excluding coaches or supportive staff) per year all-inclusive. It was assumed a similar amount of £5 for UK.

For China the costs related to coaching activities (goal setting, motivational interviewing, administration, etc.) was based on the average hours spend per individual as reported by the research team. This was 25.5 hours for China per individual per 18 months (17 hours per 12 months). The price per hour coaching time was set at 30.00, as indicated by the research team for an entry-level health-care professional. This resulted in 510 Chinese Yuan per user per year.

For the UK the costs related to coaching activities were based on a count of the full-time equivalent (FTE) related to the research and the average proportion of time spend on coaching activities as reported by the research team. This was 3 persons working for 0.80 FTE and indicated 75% time spend on coaching activities for UK for all individuals that took place over a total trial time period of 4 years. Furthermore, in total 300 individuals participated in the intervention arm of the trial in UK over an 18-month trial period and we assumed there were we assumed 40 working hours per week and 52 - 5.6 paid holiday weeks per year [www.gov.uk]. This corresponded to 29.7 hours per user per year and, with 21.75

BPN salary per hour in 2010 [Wimo, 2013] (26.94 in 2021), it corresponds to 800 BPN per user per year.

To serve 1,000 persons 21 and 12 coaches are required (assuming 45 weeks per year at 32 hours per week – 0.8 FTE).

3.7 References

- Ara R, Tumur I, Pandor A, Duenas A, Williams R, Wilkinson A, Paisley S, Chilcott J. Ezetimibe for the treatment of hypercholesterolaemia: a systematic review and economic evaluation. Health Technol Assess. 2008 May;12(21):iii, xi-xiii, 1-212. doi: 10.3310/hta12210. PMID: 18485273.
- Ara R, Pandor A, Stevens J, Rees A, Rafia R. Early high-dose lipid-lowering therapy to avoid cardiac events: a systematic review and economic evaluation. Health Technol Assess. 2009 Jul;13(34):1-74, 75-118. doi: 10.3310/hta13340. PMID: 19604457.
- Ara R, Brazier JE. Populating an economic model with health state utility values: moving toward better practice. Value Health. 2010 Aug;13(5):509-18. doi: 10.1111/j.1524-4733.2010.00700.x. Epub 2010 Mar 10. PMID: 20230546.
- Brayne C, Gao L, Dewey M, Matthews FE; Medical Research Council Cognitive Function and Ageing Study Investigators. Dementia before death in ageing societies--the promise of prevention and the reality. PLoS Med. 2006 Oct;3(10):e397. doi: 10.1371/journal.pmed.0030397. PMID: 17076551; PMCID: PMC1626550.
- Brayne C, Matthews F. THE IMPACT OF INCIDENCE PRIOR TO DEATH ON THE INCIDENCE OF DEMENTIA IN INDIVIDUALS AGED 65 YEARS AND OLDER Carol Brayne, Fiona Matthews, Cognitive Function and Ageing Study First published: 01 July 2017 https://doi.org/10.1016/j.jalz.2017.07.133
- Campbell F, Holmes M, Everson-Hock E, Davis S, Buckley Woods H, Anokye N, Tappenden P, Kaltenthaler E. A systematic review and economic evaluation of exercise referral schemes in primary care: a short report. Health Technol Assess. 2015 Jul;19(60):1-110. doi: 10.3310/hta19600. PMID: 26222987; PMCID: PMC4781341.
- Chang J, Deng Q, Guo M, Ezzati M, Baumgartner J, Bixby H, Chan Q, Zhao D, Lu F, Hu P, Su Y, Sun J, Long Y, Liu J. Trends and Inequalities in the Incidence of Acute Myocardial Infarction among Beijing Townships, 2007-2018. Int J Environ Res Public Health. 2021 Nov 23;18(23):12276. doi: 10.3390/ijerph182312276. PMID: 34886003; PMCID: PMC8656834.
- China Population and Employment Statistics Yearbook Committee. Section 2-44. National Death Population Situation by Age and Sex (November 1, 2018, to October 31, 2019), in China Population and Employment Statistics Yearbook 2020, September 2020, Beijing: China Statistics Press. Available at: https://www.zgtjnj.org/navipage-n3020013208000178.html (In Chinese)
- Chen T, Yu D, Cornelius V, Qin R, Cai Y, Jiang Z, Zhao Z. Potential health impact and cost-effectiveness of drug therapy for prehypertension. Int J Cardiol. 2017 Aug 1;240:403-408. doi: 10.1016/j.ijcard.2017.05.003. Epub 2017 May 5. PMID: 28501349.
- Fleurence RL, Hollenbeak CS. Rates and probabilities in economic modelling: transformation, translation and appropriate application. Pharmacoeconomics. 2007;25(1):3-6. doi: 10.2165/00019053-200725010-00002. PMID: 17192114.
- Gidwani R, Russell LB. Estimating Transition Probabilities from Published Evidence: A Tutorial for Decision Modelers. Pharmacoeconomics. 2020 Nov;38(11):1153-1164. doi: 10.1007/s40273-020-00937-z. Erratum in: Pharmacoeconomics. 2020 Sep 8;: PMID: 32797380; PMCID: PMC7426391.

- Landeiro F, Wace H, Ghinai I, Nye E, Mughal S, Walsh K, Roberts N, Lecomte P, Wittenberg R, Wolstenholme J, Handels R, Roncancio-Diaz E, Potashman MH, Tockhorn-Heidenreich A, Gray AM; ROADMAP Group. Resource utilisation and costs in predementia and dementia: a systematic review protocol. BMJ Open. 2018 Jan 23;8(1):e019060. doi: 10.1136/bmjopen-2017-019060. PMID: 29362261; PMCID: PMC5988053.
- Landeiro F, Mughal S, Walsh K, Nye E, Morton J, Williams H, Ghinai I, Castro Y, Leal J, Roberts N, Wace H, Handels R, Lecomte P, Gustavsson A, Roncancio-Diaz E, Belger M, Jhuti GS, Bouvy JC, Potashman MH, Tockhorn-Heidenreich A, Gray AM; ROADMAP consortium. Health-related quality of life in people with predementia Alzheimer's disease, mild cognitive impairment or dementia measured with preference-based instruments: a systematic literature review. Alzheimers Res Ther. 2020 Nov 18;12(1):154. doi: 10.1186/s13195-020-00723-1. PMID: 33208190; PMCID: PMC7677851.
- Matthews FE, Arthur A, Barnes LE, Bond J, Jagger C, Robinson L, Brayne C; Medical Research Council Cognitive Function and Ageing Collaboration. A two-decade comparison of prevalence of dementia in individuals aged 65 years and older from three geographical areas of England: results of the Cognitive Function and Ageing Study I and II. Lancet. 2013 Oct 26;382(9902):1405-12. doi: 10.1016/S0140-6736(13)61570-6. Epub 2013 Jul 17. PMID: 23871492; PMCID: PMC3906607.
- Moll van Charante EP, Hoevenaar-Blom MP, Song M, Andrieu S, Barnes L, Birck C, Brooks R, Coley N, Eggink E, Georges J, Hafdi M, van Gool WA, Handels R, Hou H, Lyu J, Niu Y, Song L, Wang W, Wang Y, Wimo A, Yu Y, Zhang J, Zhang W, Brayne C, Wang W, Richard E; PRODEMOS study group. Prevention of dementia using mobile phone applications (PRODEMOS): a multinational, randomised, controlled effectiveness-implementation trial. Lancet Healthy Longev. 2024 Jun;5(6):e431-e442. doi: 10.1016/S2666-7568(24)00068-0. Epub 2024 May 16. PMID: 38763155.
- Palmer et al. (2006) A cost-effectiveness model comparing alternative management strategies for the use of glycoprotein IIb/IIIa antagonists in non-ST-elevation acute coronary syndrome. Report to the National Institute for Clinical Excellence.
- Prince M, Acosta D, Ferri CP, Guerra M, Huang Y, Llibre Rodriguez JJ, Salas A, Sosa AL, Williams JD, Dewey ME, Acosta I, Jotheeswaran AT, Liu Z. Dementia incidence and mortality in middle-income countries, and associations with indicators of cognitive reserve: a 10/66 Dementia Research Group population-based cohort study. Lancet. 2012 Jul 7;380(9836):50-8. doi: 10.1016/S0140-6736(12)60399-7. Epub 2012 May 23. PMID: 22626851; PMCID: PMC3525981.
- Prince M, Knapp M, Guerchet M, McCrone P, Prina M, Comas-Herrera A, Wittenberg R, Adelaja B, Hu B, King D, Rehill A, Salimkumar D. 2014. Dementia UK: Update. Alzheimer’s Society 2014.
- Saka O, McGuire A, Wolfe C. Cost of stroke in the United Kingdom. Age Ageing. 2009 Jan;38(1):27-32. doi: 10.1093/ageing/afn281. PMID: 19141506.
- Strilciuc S, Grad DA, Radu C, Chira D, Stan A, Ungureanu M, Gheorghe A, Muresanu FD. The economic burden of stroke: a systematic review of cost of illness studies. J Med Life. 2021 Sep-Oct;14(5):606-619. doi: 10.25122/jml-2021-0361. PMID: 35027963; PMCID: PMC8742896.

- Velandia PP, Miller-Petrie MK, Chen C, Chakrabarti S, Chapin A, Hay S, Tsakalos G, Wimo A, Dieleman JL. Global and regional spending on dementia care from 2000-2019 and expected future health spending scenarios from 2020-2050: An economic modelling exercise. EClinicalMedicine. 2022 Mar 13;45:101337. doi: 10.1016/j.eclinm.2022.101337. PMID: 35299657; PMCID: PMC8921543.
- Wang W, Jiang B, Sun H, Ru X, Sun D, Wang L, Wang L, Jiang Y, Li Y, Wang Y, Chen Z, Wu S, Zhang Y, Wang D, Wang Y, Feigin VL; NESS-China Investigators. Prevalence, Incidence, and Mortality of Stroke in China: Results from a Nationwide Population-Based Survey of 480 687 Adults. Circulation. 2017 Feb 21;135(8):759-771. doi: 10.1161/CIRCULATIONAHA.116.025250. Epub 2017 Jan 4. PMID: 28052979.
- Ward et al. (2005) Statins for the Prevention of Coronary Events. Technology assessment report commissioned by the HTA Programme on behalf of The National Institute for Clinical Excellence. Obtained from http://www.pillole.org/public/aspnuke/downloads/documenti/statineridotto.pdf on 25-04-2022.
- Ward S, Lloyd Jones M, Pandor A, Holmes M, Ara R, Ryan A, Yeo W, Payne N. A systematic review and economic evaluation of statins for the prevention of coronary events. Health Technol Assess. 2007 Apr;11(14):1-160, iii-iv. doi: 10.3310/hta11140. PMID: 17408535.
- Wimo A, Reed CC, Dodel R, Belger M, Jones RW, Happich M, Argimon JM, Bruno G, Novick D, Vellas B, Haro JM. The GERAS Study: a prospective observational study of costs and resource use in community dwellers with Alzheimer's disease in three European countries--study design and baseline findings. J Alzheimers Dis. 2013;36(2):385-99. doi: 10.3233/JAD-122392. PMID: 23629588.
- Wübker A, Zwakhalen SM, Challis D, Suhonen R, Karlsson S, Zabalegui A, Soto M, Saks K, Sauerland D. Costs of care for people with dementia just before and after nursing home placement: primary data from eight European countries. Eur J Health Econ. 2015 Sep;16(7):689-707. doi: 10.1007/s10198-014-0620-6. Epub 2014 Jul 29. PMID: 25069577.
- Xie S, Wu J, Xie F. Population Norms for SF-6Dv2 and EQ-5D-5L in China. Appl Health Econ Health Policy. 2022 Jul;20(4):573-585. doi: 10.1007/s40258-022-00715-2. Epub 2022 Feb 8. PMID: 35132573.
- Yan X, Li F, Chen S, Jia J. Associated Factors of Total Costs of Alzheimer's Disease: A Cluster-Randomized Observational Study in China. J Alzheimers Dis. 2019;69(3):795-806. doi: 10.3233/JAD-190166. PMID: 31156170.
- Youman P, Wilson K, Harraf F, Kalra L. The economic burden of stroke in the United Kingdom. Pharmacoeconomics. 2003;21 Suppl 1:43-50. doi: 10.2165/00019053-200321001-00005. PMID: 12648034.
- Yuan J, Zhang Z, Wen H, Hong X, Hong Z, Qu Q, Tang M, Wu J, Xu Q, Li H, Cummings JL. Incidence of dementia and subtypes: A cohort study in four regions in China. Alzheimers Dement. 2016 Mar;12(3):262-71. doi: 10.1016/j.jalz.2015.02.011. Epub 2015 Jun 15. PMID: 26086181.

- Yuan, N. Li, Y. Zhang, Q. Gu, Y. Liu, J. Ma, J. Effect of different health coverage on hospital expenditure of patients with acute myocardial infarction. Shanghai Jiaotong Univ. Med. Sci. 33 (2013) 214–219.
- Yuanmei P, Yansheng L, Yan L, Ruifang W, Min S, Xuan H, et al., Study on drug cost burdens of standard secondary prevention for ischemic st roke patients in Shanghai, Shanghai Med. J. 33 (2010) 808–813. Obtained from https://www.semanticscholar.org/paper/Study-on-drug-cost-burdens-of-standard-secondary-in-Huijua/9f0d18e0885f5ce7f78d57578640199f15d247e3
- Zhang ZX, Zahner GE, Román GC, Liu J, Hong Z, Qu QM, Liu XH, Zhang XJ, Zhou B, Wu CB, Tang MN, Hong X, Li H. Dementia subtypes in China: prevalence in Beijing, Xian, Shanghai, and Chengdu. Arch Neurol. 2005 Mar;62(3):447-53. doi: 10.1001/archneur.62.3.447. PMID: 15767510.
- Zhou YF, Liu N, Wang P, Jeong Yang J, Song XY, Pan XF, Zhang X, He M, Li H, Gao YT, Xiang YB, Wu T, Yu D, Pan A. Cost-Effectiveness of Drug Treatment for Chinese Patients With Stage I Hypertension According to the 2017 Hypertension Clinical Practice Guidelines. Hypertension. 2020 Sep;76(3):750-758. doi: 10.1161/HYPERTENSIONAHA.119.14533. Epub 2020 Jul 27. PMID: 32713271; PMCID: PMC7429361.

4 Supplementary material 4: Evidence of long-term effectiveness from lifestyle prevention interventions

4.1 Evidence from literature

A systematic review indicated 2 previous dementia multidomain studies that reported on adherence [Hafdi, 2021], which were MAPT [Andrieu, 2017] and preDIVA [Moll van Charante, 2016]. MAPT intervention arm showed no significant change from baseline in adherence to physical activity; a decrease (not specified) in attendance to group sessions; a significant change from baseline in blood concentration reflective of adherence to anti-inflammatory medication (3.5%). Furthermore, it showed 90.1%, 71.9%, and 62.3% attendance to cardiovascular consultations at baseline, 1 and 2-year follow-up respectively [Coley, 2019]. preDIVA reported the intervention arm pattern of blood pressure over the trial period, with a decrease in the first 2 years and stable pattern from year 2 to year 6+ for grade II/III in grade I hypertension, and stable for 2-4 year and slightly increasing for 4-6+ year in grade I hypertension (based on visual assessment of 95% confidence intervals). In both situations the intervention was continued during the 3-6 year follow-up. However, 37% actively dropped out and 22% were non-adherent [Beishuizen, 2017].

Other reviews of dementia prevention intervention [Coley, 2022] and adherence to multidomain dementia prevention interventions [Coley, 2019] and corresponding references did not specify patterns of adherence over time, except for adherence to a physical activity intervention showing no change in adherence since baseline [Cox, 2019], and SBP change from baseline -7.5, -8.2 and -8.3 in the exercise arm and -8.1, -9.0 and -9.0 in the Omega-3s supplement arm for year 1, 2 and 3 respectively [Bischoff-Ferrari, 2020].

Outside the dementia field, a review on web-based CVD risk factor management [Beishuizen, 2016] indicated 2 studies [Shea, 2009; Cho, 2006] with more than 18-month follow-up on diabetes telemedicine interventions. Shea et al. [2009] reported a sustained effect on cholesterol and blood pressure over a 5-year period, although by visual assessment a diminishing effect for cholesterol. Cho et al. [2006] reported cholesterol at 15 and at 30 months follow-up, without a clear pattern or trend. Furthermore, in this review [Beishuizen, 2016] they reported that the effect size significantly decreased over time and that several studies in their meta-analysis reported a decreasing adherence over time.

Further supportive evidence was obtained by a specific literature search on “(adhere* OR sustain*) AND (lifestyle) AND (exercise OR physical activity) AND (prevention) AND (intervention OR randomized trial) AND (long-term)” selecting review and RCT studies with >=24 month follow-up on general or cardiovascular- and diabetes-specific lifestyle interventions (any type) not in early-life published in the past 10 years (2013-2023) from the 258 records retrieved at 226-01-2023. The >=2013 was set to obtain studies after a review of reviews by Kohl et al. [2013] who discussed “behavior change appears to be unsustainable at follow-up measures” but also referred to a “large meta-analysis [that] found that extensive use of theory and the use of multiple behavior change techniques predict effectiveness in Internet-delivered interventions”. From our review we selected the following evidence.

Uusitupa et al. [2019] concluded from a review that a lifestyle “preventive effect has been demonstrated to sustain for many years after active intervention”.

Baumann et al. [2015] concluded from a RCT in persons at high risk for cardiovascular disease “sustained effects on diet and physical activity five years after its discontinuation”.

Afshin et al. [2016] discussed in a review on mainly short-term studies that “the majority of studies consistently showed a significant decline in adherence over time” and a “need for greater evaluation of long-term […] sustainability”.

Kerrison et al. [2017] discussed from a review on diabetes prevention that “Physical exercise capacity at the end of the intense intervention period compared with the end of the trial deteriorated” and “short-term reductions are replaced by long-term weight and BMI increases”.

Lindstrom et al. [2014] plotted from a RCT a decreasing change in body weight over a 10-year period to about 50% as compared to the 1-year result.

Davis et al. [2013] concluded from an RCT that “people assigned to the […] intensive lifestyle intervention can achieve and sustain dietary change over a decade of follow-up”.

Lin et al. [2014] summarized from their review “limited information about longer-term benefits. Benefits on blood pressure, measures of weight, and glucose reduction appear to persist after 24 months, but are based on only a small subset of trials”.

An ad-hoc identified conference abstract by Lehtisalo et al. [2023] reported the lifestyle index (representing diet, physical activity, cognitive activity, and cardiovascular risk factors) was significantly higher in the intervention arm up to 7 year follow-up but no longer at 11-year follow-up in the FINGER study. The authors concluded that “a multidomain lifestyle intervention among at-risk older adults can have an impact on multidomain healthy lifestyle up to 5 years after the active intervention”.

4.2 Implementation in model

Due to the limited evidence and variation in the reported magnitude of adherence, for the base case we assumed an ad-hoc arbitrary estimate of 10% nonadherence per year and a intervention duration (and associated effect) of 10 years. This was reflected by raising the relative risk reflecting the intervention effect (RR) to the power of the adherence rate to the power of the time since model start. For example, in year 3 the intervention effect relative risk was RR^0.90^3. We assumed the effect of the intervention was presence only when exposed to the intervention, which implied nonadherence corresponded to quitting the intervention and its associated costs.

4.3 References

- Afshin A, Babalola D, Mclean M, Yu Z, Ma W, Chen CY, Arabi M, Mozaffarian D. Information Technology and Lifestyle: A Systematic Evaluation of Internet and Mobile Interventions for Improving Diet, Physical Activity, Obesity, Tobacco, and Alcohol Use. J Am Heart Assoc. 2016 Aug 31;5(9):e003058. doi: 10.1161/JAHA.115.003058. PMID: 27581172; PMCID: PMC5079005.
- Andrieu S, Guyonnet S, Coley N, Cantet C, Bonnefoy M, Bordes S, Bories L, Cufi MN, Dantoine T, Dartigues JF, Desclaux F, Gabelle A, Gasnier Y, Pesce A, Sudres K, Touchon J, Robert P, Rouaud O, Legrand P, Payoux P, Caubere JP, Weiner M, Carrié I, Ousset PJ, Vellas B; MAPT Study Group. Effect of long-term omega 3 polyunsaturated fatty acid supplementation with or without multidomain intervention on cognitive function in elderly adults with memory complaints (MAPT): a randomised, placebo-controlled trial. Lancet Neurol. 2017 May;16(5):377-389. doi: 10.1016/S1474-4422(17)30040-6. Epub 2017 Mar 27. PMID: 28359749.
- Baumann S, Toft U, Aadahl M, Jørgensen T, Pisinger C. The long-term effect of screening and lifestyle counseling on changes in physical activity and diet: the Inter99 Study - a randomized controlled trial. Int J Behav Nutr Phys Act. 2015 Mar 6;12:33. doi: 10.1186/s12966-015-0195-3. PMID: 25886540; PMCID: PMC4352560.
- Beishuizen CR, Stephan BC, van Gool WA, Brayne C, Peters RJ, Andrieu S, Kivipelto M, Soininen H, Busschers WB, Moll van Charante EP, Richard E. Web-Based Interventions Targeting Cardiovascular Risk Factors in Middle-Aged and Older People: A Systematic Review and Meta-Analysis. J Med Internet Res. 2016 Mar 11;18(3):e55. doi: 10.2196/jmir.5218. PMID: 26968879; PMCID: PMC4808240.
- Beishuizen CRL, Coley N, Moll van Charante EP, van Gool WA, Richard E, Andrieu S. Determinants of Dropout and Nonadherence in a Dementia Prevention Randomized Controlled Trial: The Prevention of Dementia by Intensive Vascular Care Trial. J Am Geriatr Soc. 2017 Jul;65(7):1505-1513. doi: 10.1111/jgs.14834. Epub 2017 Mar 6. PMID: 28263374.
- Bischoff-Ferrari HA, Vellas B, Rizzoli R, Kressig RW, da Silva JAP, Blauth M, Felson DT, McCloskey EV, Watzl B, Hofbauer LC, Felsenberg D, Willett WC, Dawson-Hughes B, Manson JE, Siebert U, Theiler R, Staehelin HB, de Godoi Rezende Costa Molino C, Chocano-Bedoya PO, Abderhalden LA, Egli A, Kanis JA, Orav EJ; DO-HEALTH Research Group. Effect of Vitamin D Supplementation, Omega-3 Fatty Acid Supplementation, or a Strength-Training Exercise Program on Clinical Outcomes in Older Adults: The DO-HEALTH Randomized Clinical Trial. JAMA. 2020 Nov 10;324(18):1855-1868. doi: 10.1001/jama.2020.16909. PMID: 33170239; PMCID: PMC7656284.
- Cho JH, Chang SA, Kwon HS, Choi YH, Ko SH, Moon SD, Yoo SJ, Song KH, Son HS, Kim HS, Lee WC, Cha BY, Son HY, Yoon KH. Long-term effect of the Internet-based glucose monitoring system on HbA1c reduction and glucose stability: a 30-month follow-up study for diabetes management with a ubiquitous medical care system. Diabetes Care. 2006 Dec;29(12):2625-31. doi: 10.2337/dc05-2371. PMID: 17130195.
- Coley N, Ngandu T, Lehtisalo J, Soininen H, Vellas B, Richard E, Kivipelto M, Andrieu S; HATICE, FINGER, and MAPT/DSA groups. Adherence to multidomain

interventions for dementia prevention: Data from the FINGER and MAPT trials. Alzheimers Dement. 2019 Jun;15(6):729-741. doi: 10.1016/j.jalz.2019.03.005. Epub 2019 Apr 30. PMID: 31047857.

- Coley N, Giulioli C, Aisen PS, Vellas B, Andrieu S. Randomised controlled trials for the prevention of cognitive decline or dementia: A systematic review. Ageing Res Rev. 2022 Dec;82:101777. doi: 10.1016/j.arr.2022.101777. Epub 2022 Nov 4. PMID: 36336171.
- Cox KL, Cyarto EV, Ellis KA, Ames D, Desmond P, Phal P, Sharman MJ, Szoeke C, Rowe CC, Masters CL, You E, Burrows S, Lai MMY, Lautenschlager NT. A Randomized Controlled Trial of Adherence to a 24-Month Home-Based Physical Activity Program and the Health Benefits for Older Adults at Risk of Alzheimer's Disease: The AIBL Active-Study. J Alzheimers Dis. 2019;70(s1):S187-S205. doi: 10.3233/JAD-180521. PMID: 30372680; PMCID: PMC6700652.
- Davis NJ, Ma Y, Delahanty LM, Hoffman HJ, Mayer-Davis E, Franks PW, Brown-Friday J, Isonaga M, Kriska AM, Venditti EM, Wylie-Rosett J; Diabetes Prevention Program Research Group. Predictors of sustained reduction in energy and fat intake in the Diabetes Prevention Program Outcomes Study intensive lifestyle intervention. J Acad Nutr Diet. 2013 Nov;113(11):1455-1464. doi: 10.1016/j.jand.2013.07.003. PMID: 24144073; PMCID: PMC3962017.
- Hafdi M, Hoevenaar-Blom MP, Richard E. Multi-domain interventions for the prevention of dementia and cognitive decline. Cochrane Database Syst Rev. 2021 Nov 8;11(11):CD013572. doi: 10.1002/14651858.CD013572.pub2. PMID: 34748207; PMCID: PMC8574768.
- Kerrison G, Gillis RB, Jiwani SI, Alzahrani Q, Kok S, Harding SE, Shaw I, Adams GG. The Effectiveness of Lifestyle Adaptation for the Prevention of Prediabetes in Adults: A Systematic Review. J Diabetes Res. 2017;2017:8493145. doi: 10.1155/2017/8493145. Epub 2017 Apr 16. PMID: 28567425; PMCID: PMC5439262.
- Kohl LF, Crutzen R, de Vries NK. Online prevention aimed at lifestyle behaviors: a systematic review of reviews. J Med Internet Res. 2013 Jul 16;15(7):e146. doi: 10.2196/jmir.2665. PMID: 23859884; PMCID: PMC3714003.
- Lehtisalo J, Ngandu T, Laatikainen T, Strandberg T, Antikainen R, Tuomilehto J, Soininen H, Kivipelto M. Adherence to healthy lifestyles during a multidomain lifestyle intervention and extended follow-up among at-risk population - the Finnish Geriatric Intervention Study to Prevent Cognitive Impairment and Disability (FINGER). Alzheimer’s Dement. 2023;19(Suppl. 23):e074576. DOI: 10.1002/alz.074576
- Lin JS, O'Connor EA, Evans CV, Senger CA, Rowland MG, Groom HC. Behavioral Counseling to Promote a Healthy Lifestyle for Cardiovascular Disease Prevention in Persons With Cardiovascular Risk Factors: An Updated Systematic Evidence Review for the U.S. Preventive Services Task Force [Internet]. Rockville (MD): Agency for Healthcare Research and Quality (US); 2014 Aug. Report No.: 13-05179-EF-1. PMID: 25232633.
- Lindström J, Peltonen M, Eriksson JG, Ilanne-Parikka P, Aunola S, Keinänen-Kiukaanniemi S, Uusitupa M, Tuomilehto J; Finnish Diabetes Prevention Study

(DPS). Improved lifestyle and decreased diabetes risk over 13 years: long-term follow-up of the randomised Finnish Diabetes Prevention Study (DPS). Diabetologia. 2013 Feb;56(2):284-93. doi: 10.1007/s00125-012-2752-5. Epub 2012 Oct 24. PMID: 23093136.

- Moll van Charante EP, Richard E, Eurelings LS, van Dalen JW, Ligthart SA, van Bussel EF, Hoevenaar-Blom MP, Vermeulen M, van Gool WA. Effectiveness of a 6-year multidomain vascular care intervention to prevent dementia (preDIVA): a cluster-randomised controlled trial. Lancet. 2016 Aug 20;388(10046):797-805. doi: 10.1016/S0140-6736(16)30950-3. Epub 2016 Jul 26. PMID: 27474376.
- Shea S, Weinstock RS, Teresi JA, Palmas W, Starren J, Cimino JJ, Lai AM, Field L, Morin PC, Goland R, Izquierdo RE, Ebner S, Silver S, Petkova E, Kong J, Eimicke JP; IDEATel Consortium. A randomized trial comparing telemedicine case management with usual care in older, ethnically diverse, medically underserved patients with diabetes mellitus: 5 year results of the IDEATel study. J Am Med Inform Assoc. 2009 Jul-Aug;16(4):446-56. doi: 10.1197/jamia.M3157. Epub 2009 Apr 23. PMID: 19390093; PMCID: PMC2705246.
- Uusitupa M, Khan TA, Viguiliouk E, Kahleova H, Rivellese AA, Hermansen K, Pfeiffer A, Thanopoulou A, Salas-Salvadó J, Schwab U, Sievenpiper JL. Prevention of Type 2 Diabetes by Lifestyle Changes: A Systematic Review and Meta-Analysis. Nutrients. 2019 Nov 1;11(11):2611. doi: 10.3390/nu11112611. PMID: 31683759; PMCID: PMC6893436.

5 Supplementary material 5: Validation and verification

The UK model external validity in terms of disease prevalence was assessed using partially dependent data. This was done by comparing the control strategy predicted age- and sex-specific prevalence of dementia, myocardial infarction and stroke with the CFASII individual patient-level data observed age- and sex-specific prevalence. For the starting population an age of 65 was chosen and the observed prevalence of MI and stroke similar to CFASII at age band 65-69. The target population reflected the general population rather than the PRODEMOS target population at increased risk. In general, a similarity between simulated and observed dementia prevalence can be seen as an indication that the combination of baseline risk factor prevalence, age- and sex-specific dementia incidence, and mortality are in balance.

Figure S5-1 shows the model-predicted prevalence and observed prevalence from CFASII data (observed prevalence by age band and sex and 95% confidence interval). Visual assessment of the graph indicates a relatively small overestimation of dementia prevalence in early age and a moderate, a relatively moderate underestimation of myocardial infarction in male and a relatively small overestimation of stroke in female. We judge this deviation is acceptable for the purpose of our model.

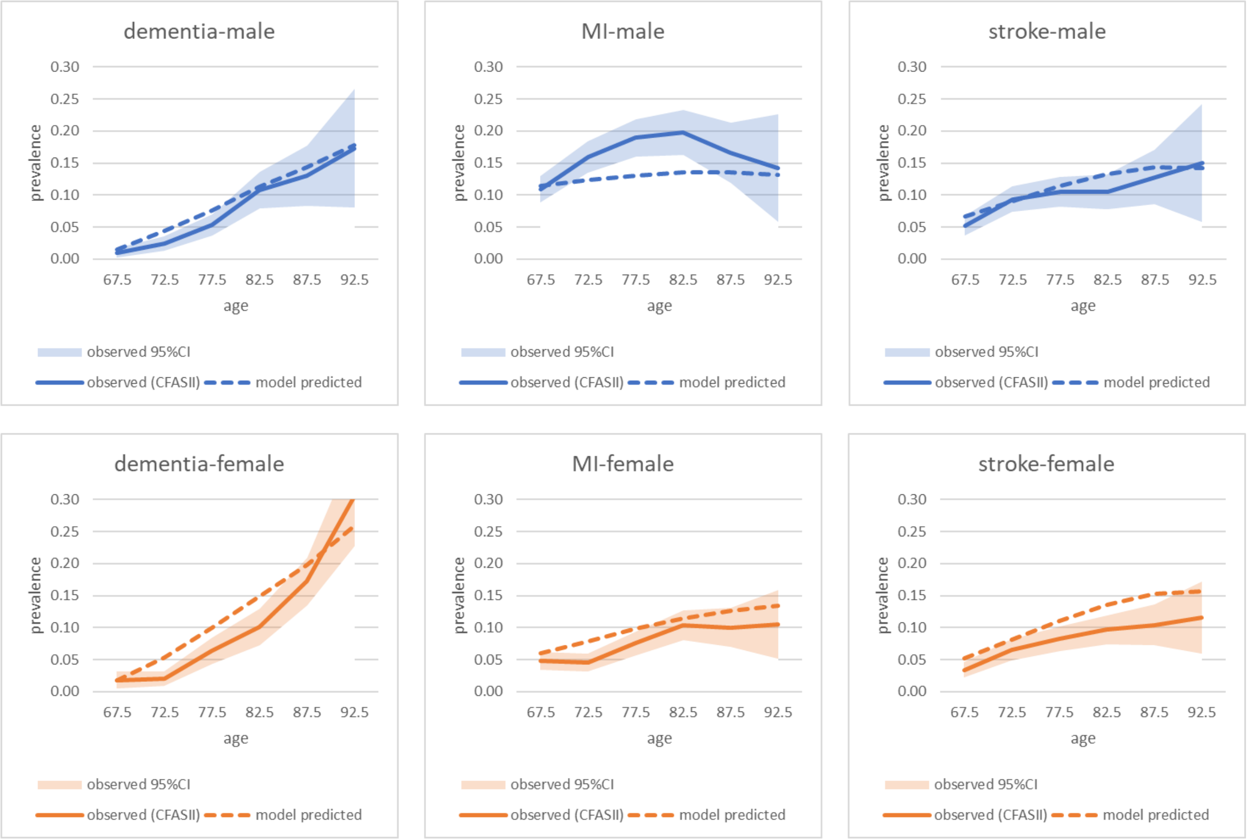

FIGURE S5-1: OBSERVED (CFASII) AND MODEL-PREDICTED PREVALENCE OF DEMENTIA, MI AND STROKE AS PART OF MODEL VALIDATION.

The lifetime risk of dementia in the Netherlands in terms of death with dementia from registry data estimated from birth was 24% [Klijs, 2021]. Our model simulated a lifetime risk of 32% up to age of 90, 40% up to age of 95 and 45% up to age of 100 from age 65 onwards. This higher estimate could be due to 1) using data from the CFASII general population observational study potentially including underdiagnosed dementia as compared to the medical setting registry data from Klijs et al. [2021], 2) overestimation by our simulation model, as is also reflected by the higher simulated prevalence in Figure S5-1.

The model internal validity was assessed by running no intervention effect in the intervention strategy (i.e., a relative risk of 1) and no intervention costs, which resulted in identical life years, quality-adjusted life years (QALY) and costs between control and intervention strategy.

In addition, the internal validity of the model was assessed by replicating the Excel model in R using the 'heemod' package (open-source available via https://github.com/ronhandels/prodemos). For this an adjusted version of the UK base case scenario was used by omitting tracking incidence (first and recurrent) and related to that applying a disutility and cost related to this (i.e., 'event in past 12 months' or 'year 1' for CVD and dementia; see supplementary material 3.6) as implementing those estimates would require manual programming outside the regular Markov model features or tunnel states complicating the model structure. The incremental net health benefit of this adjusted UK base case scenario was replicated up to 10 decimal points, also when varying some of the input estimates (i.e., ad-hoc using other input estimates and check if the ICER between the Excel and R version are identical). See Table 5-1 for the results of the Excel model when running the adjusted UK base case scenario used to for testing the replication in R. This result gave the additional insight that the omitted feature (i.e., recurrent events) did not seem to have a large impact on the health-economic results. The R version does not have the feature to estimate the cumulative incidence

Table 5-1: Results of the Excel model when running the adjusted UK base case scenario used to for testing the replication in R.

	Excel		R	
	standard of care	Inter-vention	standard of care	Inter-vention
**Cumulative incidence (per 100,000)**				
dementia onset events	44,623	44,417	n/a	n/a
MI events (including recurrent)	15,905	15,850	n/a	n/a
stroke events (including recurrent)	20,401	20,330	n/a	n/a
**Person-years (months for difference)**				
with dementia	1.8	1.8	1.8	1.8
with MI history	2.0	2.0	2.0	2.0
with stroke history	2.3	2.3	2.3	2.3
without dementia	18.8	18.9	18.8	18.9
without MI history	18.7	18.7	18.7	18.7
without stroke history	18.4	18.4	18.4	18.4
Alive	20.7	20.7	20.7	20.7
QALYs	10.51	10.52	10.51	10.52
**Costs**				
health care	14,093	13,932	14,093	13,932
informal care	19,802	19,595	19,802	19,595
platform	-	28	-	28
coaching	-	4,477	-	4,477
total	33,894	38,032	33,894	38,032
net health benefit	8.813	8.623	8.813	8.623

n/a, not replicated due to feature not replicated in R version.

5.1 References

- Klijs B, Mitratza M, Harteloh PP, Moll van Charante EP, Richard E, Nielen MM, Kunst AE. Estimating the lifetime risk of dementia using nationwide individually linked cause-of-death and health register data. Int J Epidemiol. 2021 Jul 9;50(3):809-816. doi: 10.1093/ije/dyaa219. PMID: 33354723.

6 Supplementary material 6: Heterogeneity and uncertainty

6.1 Heterogeneity

Heterogeneity was assessed by 4 scenarios reflecting a specific starting age of 60 and 70, each specifically for male and female. These were performed for their difference in observed prevalence of history of MI and stroke related to their age specific for UK and China, as well as their difference in life expectancy. The intervention effects were assumed the same although the PRODEMOS intervention study showed no significant effect in older and in male people.

In addition, we ad-hoc assessed a scenario with a starting age of 50 to reflect a scenario of early intervention providing a wider window for prevention. We note age 50 was outside the trial target population and therefore relying on the assumption that the trial effectiveness estimates observed in a population aged 55-75 can be generalized to age 50. We ad-hoc assumed the starting prevalence of myocardial infarction and stroke to be half of the prevalence at age 60. Results of this additional heterogeneity analysis are shown in Table S6-2. The results indicate a lower net benefit, which is a result of higher costs and lower QALYs, which is driven by the intervention effect waning over 10 years in combination with disease incidence increasing over time. If no waning is assumed (i.e., fully sustained intervention effect over lifetime) benefits increase with intervening at younger age.

TABLE S6-1: RESULTS OF ADDITIONAL HETEROGENEITY ANALYSES (MEAN DIFFERENCE: INTERVENTION STRATEGY MINUS STANDARD OF CARE STRATEGY).

	Cumulative incidence (per 100,000)			Person-time with (months per person)				Person-time without (months per person)			Quality-adjusted life years (per person)	Cost (per person)	Net health benefit (per person)
	Dementia	Coronary heart disease	Stroke	Dementia	Coronary heart disease	Stroke	Alive	Dementia	Coronary heart disease	Stroke			
UK													
Base case	-206	-55	-71	-0.3	0.0	0.0	0.4	0.7	0.5	0.5	0.02	4,127	-0.19
Age 50 male	-42	-39	-64	-0.1	-0.1	-0.1	0.1	0.3	0.2	0.2	0.00	4,575	-0.22
Age 50 female	-37	-69	-90	-0.2	-0.1	-0.2	0.2	0.3	0.3	0.3	0.01	4,528	-0.22
China													
Base Case	-140	-25	-38	-0.2	0.0	0.0	0.3	0.5	0.3	0.3	0.01	1,697	-0.01
Age 50 male	-20	-17	-22	-0.1	0.0	-0.1	0.1	0.2	0.1	0.2	0.00	2,696	-0.03
Age 50 female	-20	-13	-18	-0.1	0.0	-0.1	0.1	0.2	0.1	0.2	0.00	2,545	-0.03

6.2 Time horizon

The sensitivity to time horizon in terms of the maximum age up to which outcomes were simulated was assessed by taking an age of 90 and 80 (instead of 100 in the base case). This

reflects the assumption no effects are present after the age of 90 (i.e., after 90 the intervention strategy shows the same prevalence, mortality, QALYs and costs as the control strategy).

6.3 Disease and death risk methodology

First, relative risks were estimated by choosing the reported relative risks related to each risk factor as reported by the LIBRA [Deckers, 2015] and QRISK3 [Hippisley-Cox, 2017] risk scores.

The reason for this analysis was that the relative risk of CVD based on QRISK1 related to the lifestyle risk factors was relatively small compared to the relative risk for dementia mainly based on CAIDE. For dementia the RR related to obesity (BMI>30) was 2.17, for CVD the RR related to BMI (1-point change kg/m2) was 1.022 and 1.015 for men and women respectively. A change of 1 BMI point (from 30 to 29) with SD of 4.7 and assuming normal distribution corresponds to going from 50% to 42% above the threshold of 30. A 1-point change in BMI corresponds to 1.022^(29-30)=0.98 for CVD while the corresponding 8% change in obesity corresponds to 2.1^(0.42-0.50)=0.94 (2.9 times larger effect). Alternatively, to obtain the same risk change in CVD, BMI must drop with about 3 points. The same applies to SBP/hypertension, with a CVD RR of 0.96 for a 10-point mmHg change corresponding to a change in hypertension proportion of 0.22 resulting in a dementia RR of 0.85 (4.2 times larger effect). Alternatively, to obtain the same risk change in CVD, SBP must drop with about 42 points.

For this sensitivity analysis we used a different source for RR related to lifestyle factors. For dementia they were obtained by a systematic review [Deckers, 2015: table 4] and based on meta-analyses of large pooled data. For CVD they were obtained by QRISK3 [Hippisley-Cox, 2017: table 3 and 4], based on more detailed interactions for CVD making its implementation in the health-economic model more complicated. Using the results of the PRODEMOS trial and the same methods for estimating the relative risk for dementia, MI and stroke, the overall RR for dementia (using hypertension, obesity, hypercholesterolaemia, physical inactivity and smoking) was 0.945 and for CVD (using systolic blood pressure and smoking) was 0.975 (male) and 0.974 (female). This smaller (i.e., closer to 1) RR in CVD seemed mainly a result of not being able to include cholesterol (as it was the ratio and PRODEMOS trial only included total cholesterol).

Second, history of CVD was omitted to assess the sensitivity of the results in terms of person-years living with the disease due to persons living longer with a history of the disease they already had at baseline. This was operationalized by setting the history of MI and stroke to 0 at model start.

Third, a combination of the first and second was used to show the sensitivity of the results to this combination.

Fourth, as an alternative the relative risk for dementia was estimated based on the CAIDE risk score. First, by entering the difference in mean change since baseline on the 4 risk CAIDE modifiable factors, resulted in a change in the CAIDE score of -0.16 (which is the same as observed in the PRODEMOS trial [Moll van Charante, 2024]. Then, by estimating the risk in the control strategy (reflected by the CAIDE score at baseline, which is 10.9 corresponding to a 20-year dementia risk of 0.098) and by estimating the risk in the

intervention strategy (reflected by the CAIDE score at baseline + the change in CAIDE score, which is 10.8 corresponding to a 20-year dementia risk of 0.092). Dividing the 2 risks results in a relative risk of 0.945. The difference can partly be explained by the exclusion of smoking in the CAIDE risk score (which was reflected in the base case).

Fifth, a dementia-specific model was run. This was done by making the following changes to the model:

- for UK: use observed dementia age- and sex-specific estimates from [Matthews, 2016: table S1] using CFASII incident cases / 'free of dementia *2 – incident cases' for age bands; for China: same estimates as base case were used;
- for UK: 0 dementia incidence for age 50-65; for China: same as base case;
- for UK and for China: 0 CVD incidence;
- for UK and for China: mortality adjusted only for dementia (operationalized as setting the RR for death by CVD to 1).

One should be aware the observed UK dementia incidence rate was somewhat higher in mid-age, but much lower in age 85+ (as data were not provided specifically for high age bands such as 90-94 and 95-99).

Sixth, alternative sources for disease incidence rates were used. For dementia a variety of age-specific rates were available from Wolters et al. [2020: table 2]. The rate from these sources seemed higher than CFASII. For MI, an alternative incidence rate was obtained for UK from Campbell et al. [2015], which originated from a NICE technology appraisal of statins [Ward, 2005: table 4; Ward, 2007: table 49], which was based on analysing data from the Bromley Coronary Heart Disease Register in South-East London in 1996-1998 using ICD-9 code for coronary artery disease for those admitted to hospitals [Sutcliffe, 2003]. Data on acute myocardial infarction was selected from Ward et al. [2005: table 4]. The rate from this source seemed similar to CFASII but lower to CFASII for female. For stroke, an alternative incidence rate for first-ever stroke from Campbell et al. [2015], which originated from a NICE technology appraisal for statins [Ward, 2005: table 5; Ward, 2007 table 49], which originated from Bamford et al. [1988] presenting data on age- and sex-specific diagnosis of the pathological type of stroke by CT or necropsy registered by GP, hospital or death certificate. The rate from this source seemed higher than CFASII, both for males and females. The rate from this source seemed lower than Chinese rates, both for males and females. To reflect an approximate to alternative sources of incidence rates we multiplied the rates with a fixed factor. We multiplied the dementia incidence with a factor 1.5 for both sexes, both for UK and China. We multiplied the female MI rate with 0.5 for UK, and we multiplied the stroke rate with 1.5 for UK and with 0.5 for China, for both sexes.

Seventh, for the relative risk of CVD-specific mortality based on a history of CVD we used an alternative estimate from Campbell et al. [2015]. This RR estimate was 3.89 (as compared to 1.43-1.27 for UK and 4.28 for China).

Eight, the target population at increased risk as originally planned but deviated from was applied (see supplementary material 3.3).

UK-only, first, for dementia, MI and stroke incidence rate the regression GLM fitted to the CFASII data was applied to age range up to 95 instead of 90 to assess the sensitivity to uncertainty in disease incidence in high age groups.

UK-only, second, for MI and stroke the age was omitted from the CFASII GLM model as they were forced entry but not significant.

UK-only, third, a history of other disease was omitted as predictor in the CFASII GLM model (e.g., history of MI as predictor for dementia). This was done to test the sensitivity of the model for interdependencies between diseases.

UK-only, fourth, a history of other disease as well as a history of own disease was omitted as predictor in the CFASII GLM model (e.g., history of MI as predictor for dementia onset and history of MI as predictor for MI event, i.e., being recurrent event). This was done to test the sensitivity of the model for interdependencies between diseases and to own disease.

Results of UK only sensitivity analysis are shown in Table S6-2. The results of the scenario "CFASII exclude history of other disease" seemed relatively similar to the base case, implying incorporating correlations between diseases in the simulation has a relatively small impact on the results.

TABLE S6-2: RESULTS OF UK ONLY SENSITIVITY ANALYSIS (MEAN DIFFERENCE: INTERVENTION STRATEGY MINUS STANDARD OF CARE STRATEGY).

	Cumulative incidence (per 100,000)			Person-time with (months per person)				Person-time without (months per person)			Quality-adjusted life years (per person)	Cost (per person)	Net health benefit (per person)
	Dementia	Coronary heart disease	Stroke	Dementia	Coronary heart disease	Stroke	Alive	Dementia	Coronary heart disease	Stroke			
Base case	-206	-55	-71	-0.3	0.0	0.0	0.4	0.7	0.5	0.5	0.02	4,127	-0.19
CFASII fit to age 95 (UK only)	-217	-50	-76	-0.3	0.0	0.0	0.5	0.8	0.5	0.5	0.02	4,104	-0.19
CFASII MI and stroke age independent (UK only)	-204	-127	-70	-0.3	-0.1	0.0	0.4	0.7	0.5	0.5	0.02	4,127	-0.19
CFASII exclude history of other disease (UK only)	-198	-56	-75	-0.3	0.0	-0.1	0.4	0.7	0.5	0.5	0.02	4,125	-0.19
CFASII exclude history of other and own disease (UK only)	-199	-35	-48	-0.3	0.0	-0.1	0.4	0.7	0.5	0.5	0.02	4,122	-0.19

6.4 Intervention effect, cost and duration

First, a scenario was tested using the effect estimate in the PRODEMOS trial subgroup of those who were adherent to the intervention (mean difference in CAIDE score of -0.27).

Second, a scenario was tested using the effect estimate in the PRODEMOS trial subgroup of those who were planning to make a lifestyle change within 6 months (mean difference in CAIDE score of -0.33). This only affected dementia incidence using the CAIDE score mean change.

Third, a scenario was tested using the country-specific effect estimates for dementia, being mean difference in CAIDE score of -0.09 for UK and -0.20 for China.

Fourth, a scenario was tested in which the intervention was repeated without a coach while assuming the same 10% annual non-adherence rate as the base case (access to the phone application only).

Fifth and sixth, two scenarios were tested assuming lifetime full adherence (i.e., 0% no non-adherence rate) with and without coach support beyond 18 months.

Seventh and eighth, sensitivity to intervention costs were ad-hoc reflected by 1/3 of the costs (both platform and coaching) and 3 times the costs of them.

6.5 Discount rate

Discount rate was set at 1% and 7%. Sensitivity to willingness to pay was assessed by adopting 30,000 per QALY in UK and 3 times the GDP per QALY in China following guideline recommendations [NICE, 2022; Lui, 2020; Butt, 2019].

6.6 References

- Bamford J, Sandercock P, Dennis M, Warlow C, Jones L, McPherson K, Vessey M, Fowler G, Molyneux A, Hughes T, et al. A prospective study of acute cerebrovascular disease in the community: the Oxfordshire Community Stroke Project 1981-86. 1. Methodology, demography and incident cases of first-ever stroke. J Neurol Neurosurg Psychiatry. 1988 Nov;51(11):1373-80. doi: 10.1136/jnnp.51.11.1373. PMID: 3266234; PMCID: PMC1032805.
- Butt T, Liu GG, Kim DD, Neumann PJ. Taking stock of cost-effectiveness analysis of healthcare in China. BMJ Glob Health. 2019 May 14;4(3):e001418. doi: 10.1136/bmjgh-2019-001418. PMID: 31179038; PMCID: PMC6528776.
- Campbell F, Holmes M, Everson-Hock E, Davis S, Buckley Woods H, Anokye N, Tappenden P, Kaltenthaler E. A systematic review and economic evaluation of exercise referral schemes in primary care: a short report. Health Technol Assess. 2015 Jul;19(60):1-110. doi: 10.3310/hta19600. PMID: 26222987; PMCID: PMC4781341.
- Deckers K, van Boxtel MP, Schiepers OJ, de Vugt M, Muñoz Sánchez JL, Anstey KJ, Brayne C, Dartigues JF, Engedal K, Kivipelto M, Ritchie K, Starr JM, Yaffe K, Irving K, Verhey FR, Köhler S. Target risk factors for dementia prevention: a systematic review and Delphi consensus study on the evidence from observational studies. Int J Geriatr Psychiatry. 2015 Mar;30(3):234-46. doi: 10.1002/gps.4245. Epub 2014 Dec 12. PMID: 25504093.
- Hippisley-Cox J, Coupland C, Brindle P. Development and validation of QRISK3 risk prediction algorithms to estimate future risk of cardiovascular disease: prospective cohort study. BMJ. 2017 May 23;357:j2099. doi: 10.1136/bmj.j2099. PMID: 28536104; PMCID: PMC5441081.
- Lui (2020) China Guidelines for pharmacoeconomic evaluations. Obtained on 11-12-2023 from https://www.ispor.org/heor-resources/more-heor-resources/pharmacoeconomic-guidelines/pe-guideline-detail/china-mainland
- Matthews FE, Stephan BC, Robinson L, Jagger C, Barnes LE, Arthur A, Brayne C; Cognitive Function and Ageing Studies (CFAS) Collaboration. A two decade dementia incidence comparison from the Cognitive Function and Ageing Studies I and II. Nat Commun. 2016 Apr 19;7:11398. doi: 10.1038/ncomms11398.
- Moll van Charante EP, Hoevenaar-Blom MP, Song M, Andrieu S, Barnes L, Birck C, Brooks R, Coley N, Eggink E, Georges J, Hafdi M, van Gool WA, Handels R, Hou H, Lyu J, Niu Y, Song L, Wang W, Wang Y, Wimo A, Yu Y, Zhang J, Zhang W, Brayne C, Wang W, Richard E; PRODEMOS study group. Prevention of dementia using mobile phone applications (PRODEMOS): a multinational, randomised, controlled effectiveness-implementation trial. Lancet Healthy Longev. 2024 Jun;5(6):e431-e442. doi: 10.1016/S2666-7568(24)00068-0. Epub 2024 May 16. PMID: 38763155.
- NICE (2022) NICE health technology evaluations: the manual. Obtained on 11-12-2023 from http://www.nice.org.uk/process/pmg36
- Sutcliffe SJ, Fox KF, Wood DA, Sutcliffe A, Stock K, Wright M, Akhras F, Langford E. Incidence of coronary heart disease in a health authority in London: review of a community register. BMJ. 2003 Jan 4;326(7379):20. doi: 10.1136/bmj.326.7379.20. PMID: 12511455; PMCID: PMC139498.

- Ward et al. (2005) Statins for the Prevention of Coronary Events. Technology assessment report commissioned by the HTA Programme on behalf of The National Institute for Clinical Excellence. Obtained from http://www.pillole.org/public/aspnuke/downloads/documenti/statineridotto.pdf on 25-04-2022.
- Ward S, Lloyd Jones M, Pandor A, Holmes M, Ara R, Ryan A, Yeo W, Payne N. A systematic review and economic evaluation of statins for the prevention of coronary events. Health Technol Assess. 2007 Apr;11(14):1-160, iii-iv. doi: 10.3310/hta11140. PMID: 17408535.
- Wolters FJ, Chibnik LB, Waziry R, Anderson R, Berr C, Beiser A, Bis JC, Blacker D, Bos D, Brayne C, Dartigues JF, Darweesh SKL, Davis-Plourde KL, de Wolf F, Debette S, Dufouil C, Fornage M, Goudsmit J, Grasset L, Gudnason V, Hadjichrysanthou C, Helmer C, Ikram MA, Ikram MK, Joas E, Kern S, Kuller LH, Launer L, Lopez OL, Matthews FE, McRae-McKee K, Meirelles O, Mosley TH Jr, Pase MP, Psaty BM, Satizabal CL, Seshadri S, Skoog I, Stephan BCM, Wetterberg H, Wong MM, Zettergren A, Hofman A. Twenty-seven-year time trends in dementia incidence in Europe and the United States: The Alzheimer Cohorts Consortium. Neurology. 2020 Aug 4;95(5):e519-e531. doi: 10.1212/WNL.0000000000010022. Epub 2020 Jul 1. PMID: 32611641; PMCID: PMC7455342.
